# Extended phenothiazines: synthesis, photophysical and redox properties, and efficient photocatalytic oxidative coupling of amines†

**DOI:** 10.1039/d2sc01086k

**Published:** 2022-04-08

**Authors:** Jun Zhou, Lijun Mao, Meng-Xiang Wu, Zhiyong Peng, Yiming Yang, Manfei Zhou, Xiao-Li Zhao, Xueliang Shi, Hai-Bo Yang

**Affiliations:** School of Chemistry and Molecular Engineering, Shanghai Key Laboratory of Green Chemistry and Chemical Processes, East China Normal University 3663 N, Zhongshan Road Shanghai 200062 P. R. China xlzhao@chem.ecnu.edu.cn xlshi@chem.ecnu.edu.cn

## Abstract

Herein we successfully developed a ring-fusion approach to extend the conjugation length of phenothiazines and synthesized a series of novel extended phenothiazines 1–5. The intriguing π-conjugation length-dependent photophysical and redox properties of 1–5, and their photocatalytic performance towards visible-light-driven oxidative coupling reactions of amines were systematically investigated. The results indicated that this series of extended phenothiazines exhibited continuous red shifts of light absorption with increasing numbers of fused rings. As compared with the conventional phenothiazine (PTZ), all the extended phenothiazines displayed reversible redox behavior and maintained a strong excited-state reduction potential as well. Consequently, 3, 4 and 5 with longer effective conjugation lengths could efficiently catalyze the oxidative coupling of amines to imines under visible-light irradiation; by comparison, the shorter 1, 2 and PTZ could only catalyze such reactions in the presence of UV light. Moreover, 3 showed superior catalytic performance which can result in better yields within a shorter reaction time, and in a broad substrate scope. Finally, a direct and efficient conversion of amines to imines under sunlight in an air atmosphere was successfully realized. We believe that our study including the new phenothiazine modification methodology and the newly developed extended phenothiazine-based photocatalysts will open up a new way to develop novel phenothiazine-based materials for optoelectronic and catalytic applications.

## Abstract 2

Herein, we successfully developed a ring-fusion approach to extend the conjugation length of phenothiazines that were demonstrated to be efficient photocatalysts for visible-light-driven oxidative coupling reactions of amines under an air atmosphere.

## Graphical abstract

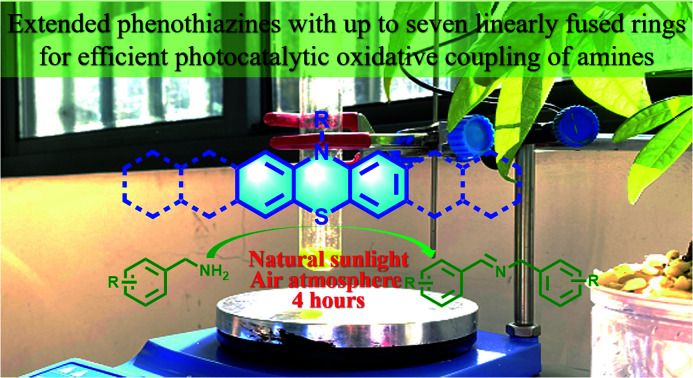

## Introduction

**Fig. 1:**
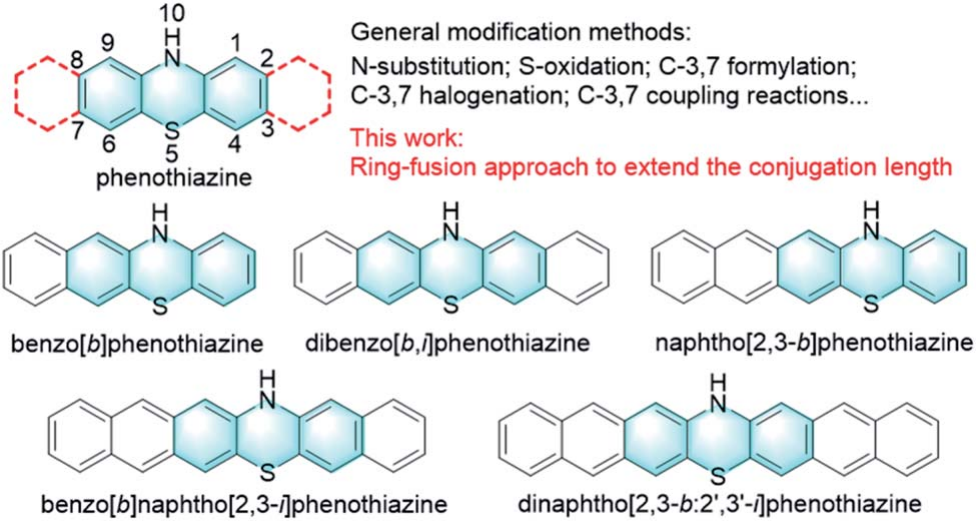
Target extended phenothiazine cores and design principle of this work.

Phenothiazine, an S, N heterocyclic molecule, is a well-known and highly versatile building block for a broad range of applications in different research areas due to its easy chemical functionalization and intriguing chemical and physical properties (Fig. 1).^1,2^ For example, phenothiazine and its derivatives have been widely utilized as donor units in the molecular engineering of highly conjugated donor–acceptor materials for optoelectronics, which is largely attributed to the electron-rich nature of sulphur and nitrogen heteroatoms.^3–5^ Also, because phenothiazine derivatives exhibit low oxidation potentials with a fully reversible one-electron redox process and good hole mobility, they have been widely employed as efficient hole transport materials (HTMs) for perovskite solar cells (PSCs).^6–8^ In addition, phenothiazine is a very unique luminophore that can display conventional fluorescence and thermally activated delayed fluorescence (TADF) as well as room-temperature phosphorescence (RTP), mainly because of its distinct boat conformation that can induce different charge transfer characteristics and molecular packing motifs.^9–15^ Moreover, phenothiazine derivatives have also been proven to be effective photoredox catalysts for various synthetic transformations ranging from metal-free atom transfer radical polymerization (ATRP) for controlled polymer synthesis^16–23^ to small molecule transformations such as radical dehalogenations,^24^ nucleophilic alkoxylations of alkyl olefins,^25^ photoredox catalyzed C–N and C–H/C–H cross-couplings,^26,27^ and so on.^28,29^ Despite these diverse electronic and optical properties and wide applications, the development of phenothiazine-based materials is limited primarily to the chemical modification of the phenothiazine core at N, S sides and 3, 7 positions (Fig. 1).^30^ Specifically, the functional positions of the phenothiazine core, N-10, S-5, and C-3,7, are typically involved in chemical reactions like *N*-substitution, oxidation, formylation, and electrophilic substitutions (halogenation) and subsequent coupling reactions. Compared to these conventional modification strategies, Matyjaszewski and co-workers reported a phenyl benzo[*b*]phenothiazine with extended conjugation, which demonstrated stronger absorption in the visible light region and better catalytic performance than those of 10-phenylphenothiazine.^31^ On this basis, we speculate that the synthesis of extended phenothiazines would be an alternative strategy to tune the electronic and optical properties of phenothiazine-based materials tailored to specific applications, especially for their optoelectronic and catalytic applications (Fig. 1).

Imines refer to a class of organic molecules containing a carbon–nitrogen double bond which have been widely employed as important building blocks for the synthesis of organic fine chemicals and pharmaceutical compounds.^32^ Imines can be prepared by many methods such as the condensation of aldehydes and ketones with amines, the reaction of nitriles with organometallic compounds, dehydrogenation of amines, reduction of nitro compounds, and so on.^33^ Alternatively, imines can also be obtained by the direct oxidative coupling of amines, which shows the significant advantages of high atom economy and environment friendliness.^34^ However, the oxidative coupling of amines to imines is usually carried out using expensive noble metal catalysts at a high reaction temperature.^35^ Recently, the photocatalytic oxidative coupling of amines has been emerging as a promising approach for imine synthesis due to the advantages of low energy consumption and high efficiency.^36–38^ For example, some representative photocatalytic systems, such as transition metal catalysts,^39,40^ metal oxides,^41^ metal/semiconductor hybrid nanostructures,^42,43^ metalloporphyrins,^44,45^ and metal–organic frameworks (MOFs),^46,47^ have proven to be very efficient for oxidative coupling of a variety of amines. However, most of the above are still metal-containing photocatalysts, while metal-free, pure organic and visible light-active photocatalysts for efficient oxidative coupling of amines are relatively rarely reported.^48,49^ Besides, many reported photocatalytic systems for oxidative coupling of amines are basically polymer-based hybrid materials; however, there is little research on the potential of organic small molecules as photocatalysts for imine preparation. In addition, organic small molecules could offer more design space to fine tune their optoelectronic and redox properties and are particularly convenient for the understanding of the structure–property relationships at the molecular level, owing to their molecularly precise backbone. In this scenario, the development of novel small-molecule photoredox catalysts for efficient imine synthesis is of great importance.

In this work, we report the design and synthesis of a series of extended phenothiazines 1–5 with up to seven linearly fused rings, including benzo[*b*]phenothiazine, dibenzo[*b*,*i*]phenothiazine, naphtho[2,3-*b*]phenothiazine, benzo[*b*]naphtho[2,3-*i*]phenothiazine and dinaphtho[2,3-*b*:2′,3′-*i*]phenothiazine, through a simple and versatile synthetic strategy (Fig. 1). As expected, extension of the conjugation of phenothiazines obviously shifted their absorption spectra toward the longer wavelength region, along with the significant increase in the molar extinction coefficient. Moreover, all the extended phenothiazines displayed reversible redox behavior and can be oxidized to form the corresponding radical cation species with considerable stability. Subsequently, the catalytic performances of 1–5 and PTZ were surveyed in the ultraviolet (UV) and visible-light-driven oxidative coupling reactions of amines. The results indicated that 3 showed better catalytic performance that not only resulted in a broad substrate scope, but also succeeded in the efficient conversion of amines to imines under sunlight in an air atmosphere. Therefore, for the first time this work provided a new strategy to modify phenothiazines and successfully developed a very efficient pure organic and visible light-active photocatalyst for efficient oxidative coupling of amines.

## Results and discussion

### Molecular design and synthesis

**Scheme 1:**
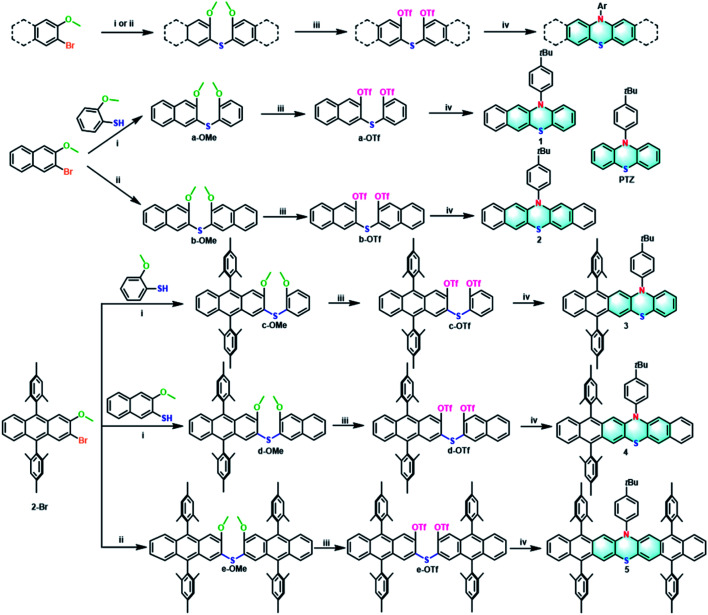
General synthetic procedures of 1–5: (i) Pd_2_(dba)_3_, XPhos, *t*-BuONa, toluene, 130 °C, 24 h, yields: 56% (a-OMe), 60% (c-OMe), 49% (d-OMe); (ii) thiourea, Pd_2_(dba)_3_, TriPhos, Cs_2_CO_3_, 1,4-dioxane, 110 °C, 24 h, yields: 56% (b-OMe), 59% (e-OMe); (iii) (a) BBr_3_, CH_2_Cl_2_, 0 °C to room temperature, 1 h; (b) triflic anhydride, pyridine, CH_2_Cl_2_, 0 °C to room temperature, 2 h, yields: 77% (a-OTf), 83% (b-OTf), 76% (c-OTf), 61% (d-OTf), 70% (e-OTf); (iv) 4-(*tert*-butyl)aniline, Pd_2_(dba)_3_, DPEPhos, Cs_2_CO_3_, toluene, 130 °C, 24 h, yields: 49% (1), 36% (2), 60% (3), 49% (4), 48% (5).

For comparison, all the target extended phenothiazines 1–5 and the reference PTZ were designed with the same substituent of 4-*tert*-butylphenyl group on the nitrogen atom. Considering the solubility issue of the longer extended phenothiazines 3–5, two bulky mesityl groups are used to suppress their π–π stacking. As shown in Scheme 1, a simple and versatile synthetic strategy involving the synthesis of the methoxy-substituted diaryl sulfides, the conversion of methoxy groups to triflates, and a final palladium-catalyzed double *N*-arylation reaction^50^ was employed to prepare this series of extended phenothiazines (see the ESI†). Specifically, the methoxy-substituted diaryl sulfides could be obtained by the normal palladium-catalyzed cross-coupling reaction of aryl halides with aryl thiols, or by the direct palladium-catalyzed C–S coupling of aryl halides with thiourea. For example, a-OMe, c-OMe, and d-OMe could be obtained by the cross-coupling reaction of 2-bromo-3-methoxynaphthalene or 2-bromo-9,10-dimesityl-3-methoxyanthracene with corresponding 2-methoxybenzenethiol or 3-methoxynaphthalene-2-thiol in good yields. By comparison, the symmetric b-OMe and e-OMe could be directly prepared by the C–S coupling of the corresponding 2-bromo-3-methoxynaphthalene and 2-bromo-9,10-dimesityl-3-methoxyanthracene with thiourea in 56% and 59% yields, respectively. Subsequently, the obtained methoxy-substituted diaryl sulfides were demethylated using boron tribromide and were then converted into triflates a-OTf–e-OTf in a reaction with triflic anhydride in the presence of pyridine in moderate yields. Finally, phenothiazines 1–5 were synthesized in moderate to good yields from triflates a-OTf–e-OTf and 4-*tert*-butylaniline by the palladium-catalyzed double *N*-arylation reaction. Phenothiazines 1–5 and other newly synthesized compounds were well-characterized by ^1^H NMR, ^13^C NMR, and mass spectrometry (see the ESI†). Particularly, the structures of 1–5 were all unambiguously confirmed by X-ray crystallographic analysis (*vide infra*).

### Photophysical and electrochemical properties

**Fig. 2:**
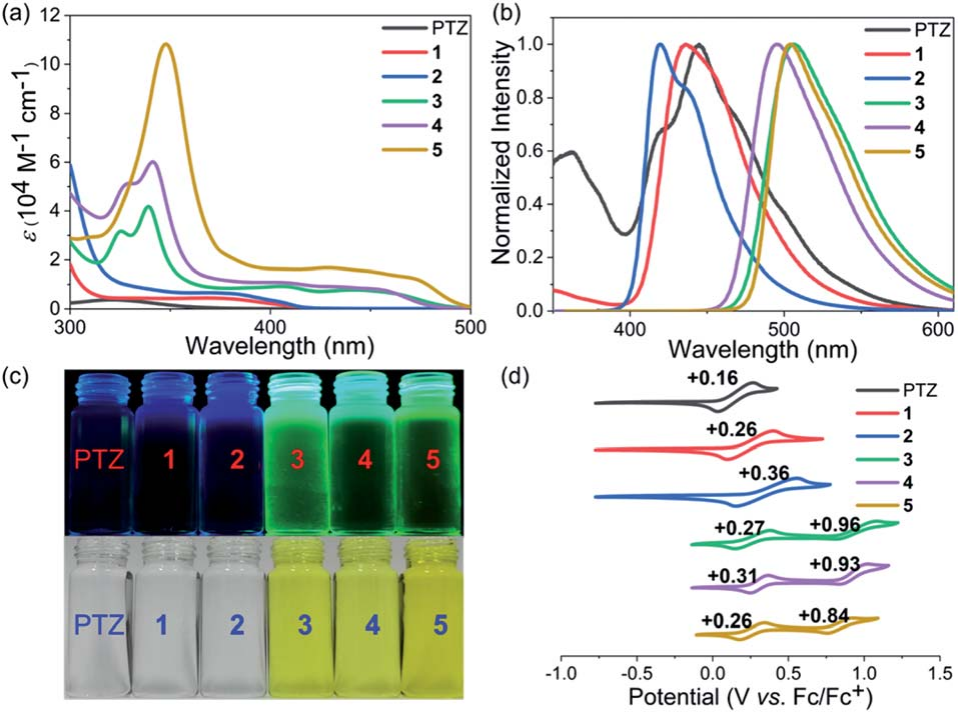
UV-vis absorption spectra (a) and normalized emission spectra (b) of PTZ and 1–5. The excitation wavelength of PTZ and 1–5 was 321, 368, 371, 339, 341, and 348 nm, respectively. (c) Photos of the solutions under white light (bottom) and upon irradiation (top). (d) Cyclic voltammograms of PTZ and 1–5 in CH_2_Cl_2_ with 0.1 M Bu_4_NPF_6_ as the supporting electrolyte, Ag/AgCl as the reference electrode, and a Pt wire as the counter electrode and a scan rate at 20 mV s^−1^.

Phenothiazines 1–5 showed good solubility in common organic solvents, and their UV-vis absorption and emission spectra were recorded in *N*,*N*-dimethylformamide (Fig. 2a–c). As expected, due to the more extended conjugation, phenothiazines 1–5 showed significant red-shift absorption spectra with respect to the reference PTZ. Another noticeable feature of their absorption profiles was that increasing the number of fused rings also enhanced the absorption intensity and shifted wavelengths to low energies (Table 1). In particular, for the solutions of 3–5, obvious absorption bands between 400 nm and 500 nm were observed, resulting in their yellow color. In contrast, 1 and 2, like PTZ, were almost colorless. Regarding their emission spectra, 1, 2 and PTZ emitted very weak blue fluorescence (400–500 nm) while 3–5 displayed a green emission band between 450 nm and 600 nm. Their fluorescence quantum yield (*Φ*_F_) values were then estimated by the absolute integrating sphere method and the results indicated that the extended phenothiazines generally exhibited higher *Φ*_F_ values, especially for 3–5 (Table 1).

**Table 1:** Summary of the photophysical and electrochemical data^a^

Photocatalyst	*λ* ^abs^_max_ [nm]	*ε* [M^−1^ cm^−1^]	*λ* ^em^_max_ [nm]	*Φ*_F_ [%]	*E*_1/2_ *vs.* SCE [V]	*E*_1/2_ (PC˙^+^/PC*) *vs.* SCE [V]	*E* ^opt^_g_ [eV]	*E*_ox1_ [V]	*E*_ox2_ [V]	*E*_HOMO_ [eV]	*E*_LUMO_ [eV]
PTZ	321	3700	445	<1	+0.62	−2.17	3.66	0.24	—	−5.04	−1.38
1	368	4467	436	<1	+0.72	−2.12	3.16	0.34	—	−5.14	−1.98
2	371	6634	418	7.22	+0.82	−2.15	3.04	0.44	—	−5.24	−2.20
3	339	41 867	506	19.54	+0.73	−1.72	2.61	0.38	0.99	−5.18	−2.57
4	341	60 167	494	11.59	+0.77	−1.74	2.66	0.33	0.97	−5.13	−2.47
5	348	108 333	504	12.23	+0.72	−1.74	2.56	0.36	0.91	−5.16	−2.60

a *λ* ^abs^_max_: absorption maxima. *ε*: molar extinction coefficient for the corresponding absorption maximum. *λ*^em^_max_: emission maxima. *Φ*_F_: fluorescence quantum yield. *E*_1/2_: half-wave potential. *E*_1/2_ (PC˙^+^/PC*): excited state reduction potential. *E*^opt^_g_: optical gaps. *E*_ox_: first oxidation potentials *versus* ferrocene/ferricenium (Fc/Fc^+^). *E*_HOMO_: HOMO energies. *E*_LUMO_: LUMO energies.

The electrochemical properties of phenothiazines 1–5 were investigated by performing cyclic voltammetry (CV) in a dry CH_2_Cl_2_ solution and compared with those of PTZ (Fig. 2d). Interestingly, PTZ, 1 and 2 were found to exhibit one reversible oxidation wave with half-wave potentials (*E*_1/2_) of 0.16, 0.26, and 0.36 V (*vs.* Fc/Fc^+^), respectively. In contrast, phenothiazines 3–5 showed two reversible oxidation waves with half-wave potentials of 0.27/0.96, 0.31/0.93, and 0.26/0.84 V, respectively. The HOMO energy levels of PTZ and 1–5 were determined to be −5.04, −5.14, −5.24, −5.18, −5.13, and −5.16 eV, respectively, based on the onset potential of the first oxidation wave. Notably, the results revealed that the extension of conjugation length exerted less impact on the HOMO orbital of phenothiazines, but the change in their LUMO energy levels was more obvious based on the calculation results (Fig. S1†). Thus, the variation of the HOMO–LUMO band gap of PTZ and 1–5 was mainly attributed to the more pronounced decrease of the LUMO level with the increase of conjugation length (Table 1 and Fig. S1†). Combining their electrochemical results with optical spectroscopy, the excited-state reduction potentials of PTZ and 1–5 were then determined to be approximately −2.17, −2.12, −2.15, −1.72, −1.74 and −1.74 V (*vs.* SCE), respectively. Though the reducing ability of 1–5 tended to be weaker than that of PTZ, this series of extended phenothiazines still maintained a strong excited-state reduction potential when compared with the classical metal–ligand complex molecules such as Ru(bpy)_3_^2+^ and Ir(ppy)_3_^2+^,^51,52^ indicating their potential as good photocatalysts. Therefore, the above photophysical and electrochemical studies disclosed that the ring-fusion approach could efficiently tune the photophysical and redox properties of phenothiazines, especially of 3–5, and consequently would have a significant effect on their catalytic performance (*vide infra*).

### The properties of radical cation species

**Fig. 3:**
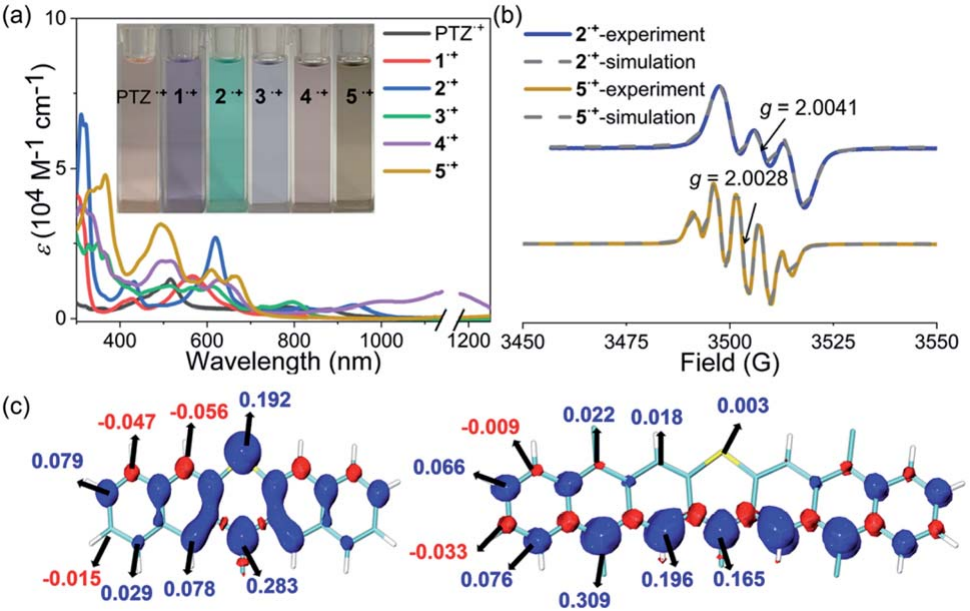
(a) UV-vis-NIR absorption spectra of the radical cation species of PTZ˙^+^ and 1˙^+^–5˙^+^. Inserted are the photos of the solutions. (b) EPR spectra of 2˙^+^ and 5˙^+^, and their simulations. (c) Calculated spin density distributions of 2˙^+^ (left) and 5˙^+^ (right).

The stability of the radical cation or anion intermediates of photocatalysts is very important to their practical synthetic transformations because only robust photocatalysts are able to perform many catalytic cycles. Therefore, the properties of the radical cation species of PTZ and 1–5 are of great interest and were then systematically investigated. The observed highly reversible oxidation waves of PTZ and 1–5 based on their CV spectra implied that their corresponding radical cation species were theoretically stable and accessible. Chemical oxidation of PTZ and 1–5 with AgSbF_6_ oxidant was explored, and the oxidation species were investigated by UV-vis-NIR absorption spectroscopy (Fig. 3a and S10†). The oxidation processes were monitored by UV-vis-NIR titrations, and the significant color change and the emergence of the distinct long-wavelength absorptions together with the observed isosbestic points all indicated the successful formation of radical cation species 1˙^+^–5˙^+^. Notably, the dication species of 3^2+^–5^2+^ were not accessible regardless of the fact that 3–5 displayed two reversible oxidation waves, mainly due to the modest oxidation ability of AgSbF_6_. Interestingly, the UV-vis-NIR absorption profiles of 1˙^+^ and 2˙^+^ were actually quite similar to that of PTZ˙^+^ which all displayed a prominent band in the ultraviolet region and a broad high intensity absorption in the visible region. In contrast, the features of the UV-vis-NIR absorption of 3˙^+^–5˙^+^ were significantly distinct from those of PTZ˙^+^, 1˙^+^ and 2˙^+^, indicating that these radical cation species may feature diverse electronic structures. During the chemical oxidation processes it was also found that radical cation species of 3˙^+^–5˙^+^ were more reactive than PTZ˙^+^, 1˙^+^ and 2˙^+^, *e.g.*, 3˙^+^–5˙^+^ were prone to being restored to their neutral state when kept under ambient conditions.

Electron paramagnetic resonance (EPR) spectroscopy was then employed to investigate the electronic structures of 1˙^+^–5˙^+^ (Fig. 3b and Table S2†). The observed intensive EPR signals further provided direct evidence of the successful formation of the radical species 1˙^+^–5˙^+^. Interestingly, 1˙^+^ and 2˙^+^, like PTZ˙^+^, displayed three characteristic peaks due to the nitrogen hyperfine splitting (*m*_I_ = 0, ±1) with a *g* tensor around 2.004–2.005.^53^ By comparison, the EPR spectra of 3˙^+^–5˙^+^ displayed strikingly different isotropic hyperfine splitting patterns compared with those of 1˙^+^, 2˙^+^ and PTZ˙^+^. Moreover, the *g* values of 3˙^+^–5˙^+^ also decreased continuously and were obviously smaller than those of PTZ˙^+^, 1˙^+^ and 2˙^+^. In order to gain more insights into their electronic structures, density functional theory (DFT) calculations at the M06-2X/def2-TZVP level were further conducted to elucidate the spin density distributions in PTZ˙^+^ and 1˙^+^–5˙^+^ (Fig. 3c and S2–S7†). The calculated results revealed that 1˙^+^ and 2˙^+^, like PTZ˙^+^, featured the same spin density distribution map as their spin density was mainly located on the heteroatoms of both nitrogen and sulphur. In comparation, the spin density distribution maps of 3˙^+^–5˙^+^ were very distinct from those of 1˙^+^ and 2˙^+^, wherein negligible spin density was observed in the sulphur atoms while the spin was delocalized over the outermost aromatic rings. This well explained the relatively large *g* values of PTZ˙^+^, 1˙^+^ and 2˙^+^ and the smaller *g* values of 3˙^+^–5˙^+^, *i.e.*, the latter featured more carbon-centered radical character and the *g* values approached a *g*_e_ value of 2.003. Also, because 3˙^+^–5˙^+^ behaved more like the carbon-centered radical, they were more reactive than PTZ˙^+^, 1˙^+^ and 2˙^+^. Based on the calculation results, their EPR spectra were also roughly simulated using the Bruker SpinFit software (Fig. 3b and S11–S16†). Taking 2˙^+^ and 5˙^+^ as examples, both simulated spectra satisfactorily agreed with the experimentally observed spectrum, wherein the three-line splitting of 2˙^+^ was due to the isotropic ^14^N hyperfine coupling (*A*_N_ = 7.289 G) while the five-line splitting pattern of 5˙^+^ was caused by the ^14^N hyperfine coupling (*A*_N_ = 5.478 G) and proton superhyperfine splitting (*A*_H_ = 4.597 G). Therefore, these results revealed that the extension of conjugation in this series of phenothiazines produced a significant impact on the properties of their radical cation species, especially their electronic structures and stability.

### X-ray crystallographic analysis

**Fig. 4:**
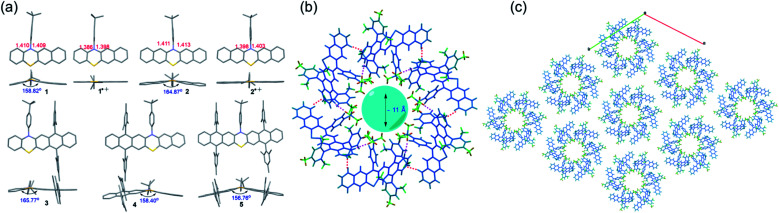
(a) Single crystal structures including the top view and side view of 1–5, 1˙^+^ and 2˙^+^. (b) Hexamer organization of 4. (c) Crystal packing of 4, viewed along the *c* axis (one layer) showing the stacking of the molecules along the *a* and *b* directions.

Single crystals of 1–5 and radical cation species of 1˙^+^ and 2˙^+^, suitable for X-ray crystallography analysis, were successfully grown and analyzed (Fig. 4 and S17–S23†). Notably, all attempts to obtain the single crystals of 3˙^+^–5˙^+^ were unsuccessful because during the chemical oxidation of 3–5 and crystal growth process, single crystals of neutral 3–5 rather than 3˙^+^–5˙^+^ always formed when the crystals precipitated out, which might indicate that the weakly coordinating anion of SbF_6_^−^ was not sufficient to stabilize 3˙^+^–5˙^+^. Like the conventional phenothiazine derivatives, 1–5 all exhibited a bent structure along the S–N axis with a small bending angle of around 155°–165° (Fig. 4a). The substituents of the 4-*tert*-butylphenyl group and the bulky mesityl groups oriented almost perpendicularly to the backbone, which was conducive to their good solubility. Upon oxidation, the phenothiazine skeletons of the formed radical cation species 1˙^+^ and 2˙^+^ tended to be nearly planar. In addition, the length of the C–N bond and C–S in the phenothiazine skeleton of 1 and 2 became significantly shorter after oxidation. These tendencies of planarization of the PTZ skeleton and decreased bond lengths are commonly observed in the π-electronic system of the PTZ radical cation because the radical cation is delocalized over the planarized π-system and the N and S atoms, which could significantly increase its stability. In contrast, these tendencies were not observed in the DFT-optimized geometries of 3˙^+^–5˙^+^ (Fig. S8 and Table S1†), indicating that the electronic structures, especially their spin density distributions, were markedly different from those of conventional PTZ radical cations as well as 1˙^+^ and 2˙^+^. Regarding their 3D packing structures, common π–π stacking or herringbone arrangements in conventional acene/heteroacene derivatives were not observed in 1–5, mainly because of their bent conformation and the bulky substituents. In contrast, face to face π–π stacking was observed in 1˙^+^ and 2˙^+^, which could mostly be attributed to their highly planar structures. It is worth mentioning that the crystals of 4 self-assembled into a hexamer with a very interesting 3D packing motif (Fig. 4b). Specifically, six molecules of 4 formed a hexamer with a ∼11 Å cavity. The hexamer structure was mainly stabilized by multiple C–H⋯H–C (pink dotted line) and C–H⋯π (red dotted line) interactions between the six molecules. The hexamers propagated along the *a* and *b* axes and then self-assembled into a 3D porous supramolecular network (Fig. 4c). The intrinsic cavity and intriguing 3D packing structures of 4 indicated that it may have some potential applications in gas storage and separation.

### Catalytic performance studies

**Fig. 5:**
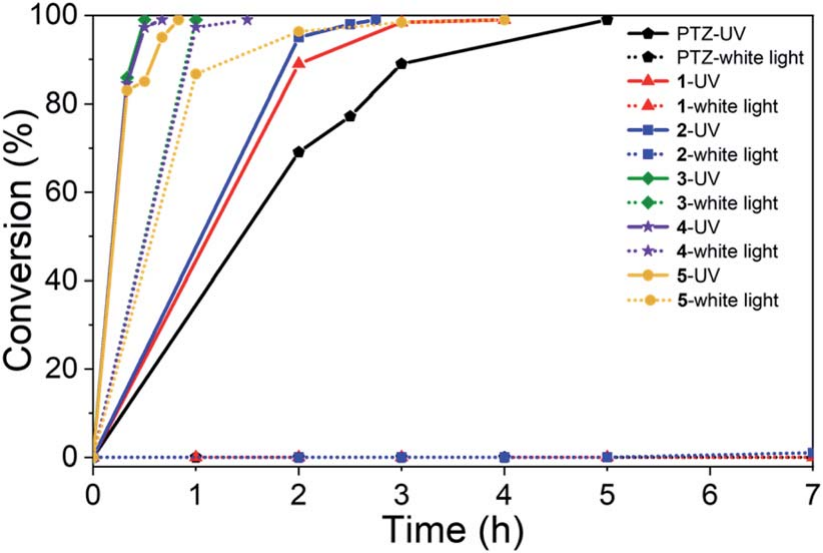
Catalyst activity results of PTZ and 1–5 for the oxidative coupling reaction of benzylamine to imine under 6 W UV light and 6 W white LED light. Reaction conditions: benzylamine (0.5 mmol), catalyst (0.25 mol%), toluene (1.5 mL), O_2_ balloon, 6 W UV or white LED light, room temperature. Conversion was determined by GC using dodecane as an internal standard and confirmed by GC-MS.

To survey the effectiveness of 1–5 as catalysts in the oxidative coupling of amines to imines, a model reaction based on the substrate of benzylamine and some controlled reactions were carried out under different irradiation conditions. The results indicated that all the extended phenothiazines, like the reference PTZ, were capable of catalyzing the oxidative coupling of benzylamine to the imine efficiently at room temperature with UV light and 1 atm of oxygen as the oxidant (Fig. 5). Interestingly, the extended phenothiazines, especially 3–5, were demonstrated to be more efficient than PTZ because they took comparatively little time to achieve a >99% conversion. Moreover, the difference in catalytic activity became more obvious when the reaction was conducted under white LED irradiation (the LED light intensity is 225 mW cm^−2^, and the wavelength range is between 400 nm and 830 nm). Specifically, the oxidative coupling reaction catalyzed by PTZ, 1, or 2 hardly occurred at all, while the reaction could still proceed efficiently in the presence of 3–5. The underlying cause of their distinct catalytic activity, especially under white light, was their different absorption characteristics where PTZ, 1, and 2 were almost colorless and thus unable to catalyze the oxidative coupling reaction. Notably, 3 showed slightly superior catalytic performance than 4 and 5, which was likely to be due primarily to its relatively better photostability among them (Fig. S24 and S25†). Therefore, 3 was chosen as a representative of this series of extended phenothiazines to demonstrate their potential photocatalytic applications. To further demonstrate the excellent catalytic performance of 3, the reaction scope was then explored with a range of amine substrates bearing different substituted groups (Table 2). As expected, all simple amine substrates were converted into the corresponding imine products with low catalyst loadings in good to excellent conversion and yield within 1 h (Table 2, entries 1–6). The conversion and the yield of the heteroaryl amines and amines with electron-withdrawing groups were slightly decreased (Table 2, entries 7–9), mainly due to the electronic effects. In addition, 1,2,3,4-tetrahydroisoquinoline and dibenzylamine could also be converted to corresponding imines (Table 2, entries 10 and 11).^54^

**Table 2:** Visible-light-driven oxidative coupling of various amines to imines catalyzed by 3^a^

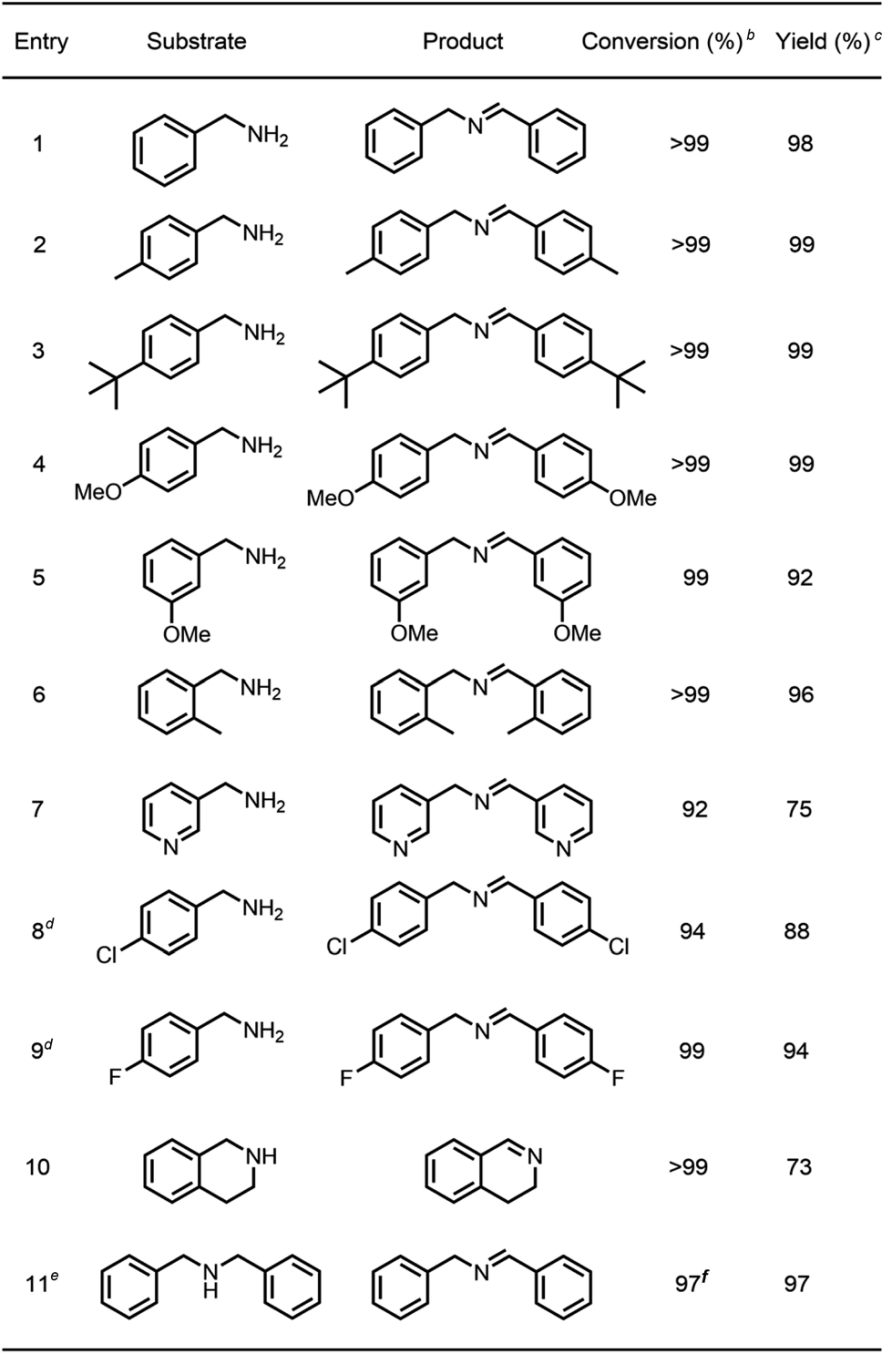

a Reaction conditions: amine (0.5 mmol), catalyst 3 (0.25 mol%), C_6_D_6_ (1.5 mL), O_2_ balloon, 6 W white LED light, room temperature, 1 h.

b Conversion was determined by GC using dodecane as an internal standard and confirmed by GC-MS.

c Yield was determined by ^1^H NMR using 1,3,5-trimethoxybenzene as an internal standard.

d Catalyst 3 (0.5 mol%), 1.5 h.

e Catalyst 3 (0.5 mol%), 1 h.

f Conversion was determined by ^1^H NMR using 1,3,5-trimethoxybenzene as an internal standard.

Generally, most of the reported photocatalytic oxidative couplings of amines to imines required high catalyst loading or extremely long reaction times, and were usually carried out under an artificial light source (*e.g.*, LED light).^48,54–56^ In comparison, phenothiazine 3 as a photocatalyst for oxidative coupling of amines demonstrated several merits such as low catalyst loadings, short reaction times and mild conditions (Fig. S26†). Encouraged by the results described above, natural-sunlight-driven oxidative coupling reactions of amines to imines catalysed by 3 were then demonstrated. To one's delight, a series of simple substituted benzylamines were successfully converted to the corresponding imines in good to excellent conversion and yields in the presence of low loading amount of 3 when the reactions were conducted under sunlight in an air atmosphere (Table 3 and Fig. S27†). To the best of our knowledge, these preliminary results indicated that 3 represents one of the most efficient photocatalysts for the visible-light-driven oxidative coupling reactions of amines at room temperature so far.

**Table 3:** Sunlight-driven oxidative coupling of various amines to imines catalyzed by 3^a^

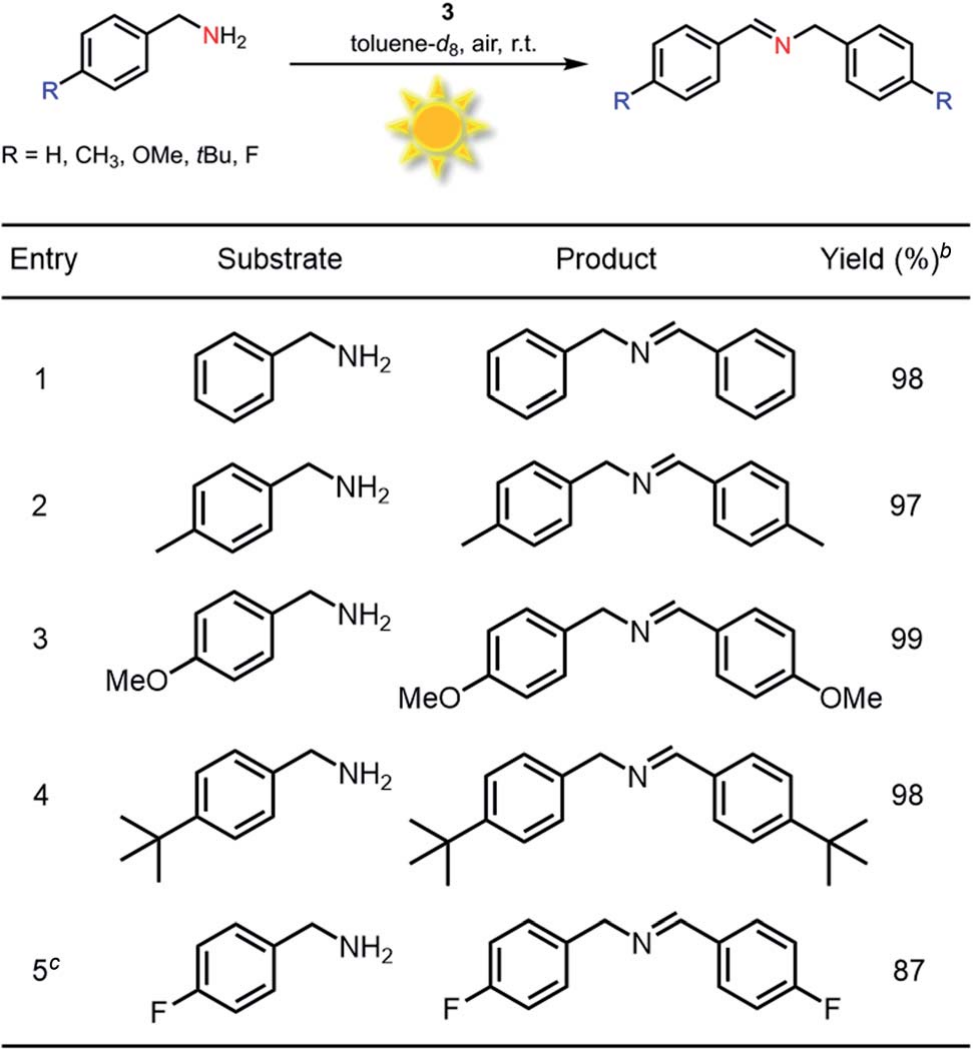

a Reaction conditions: amine (0.5 mmol), catalyst 3 (0.25 mol%), toluene-*d*_8_ (1.5 mL), air, natural sunlight, room temperature, 4 h.

b Yield was determined by ^1^H-NMR using 1,3,5-trimethoxybenzene as an internal standard.

c Catalyst 3 (0.5 mol%).

**Fig. 6:**
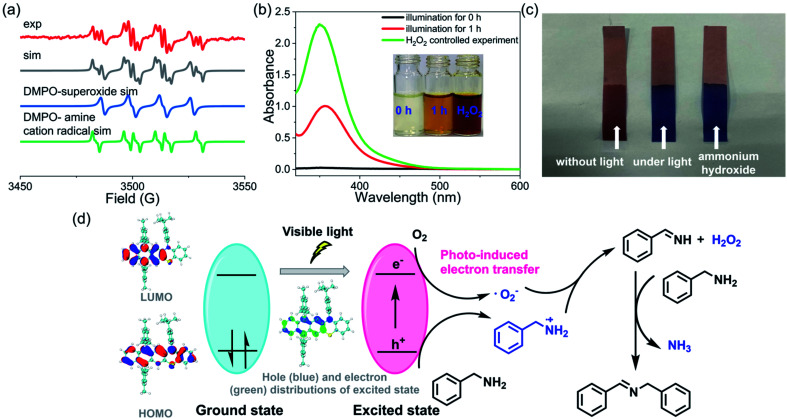
Reaction mechanism studies and analysis. (a) DMPO spin-trapping EPR spectra recorded with 3 and benzylamine under visible-light irradiation, and their simulations. (b) Test for the production of hydrogen peroxide by adding KI. (c) Test for the production of ammonia using litmus paper. (d) Proposed pathway for oxidative coupling reactions of amines.

To further understand the details of the oxidative coupling reaction, the reaction mechanism was then systematically investigated. Generally, in these photoredox catalysis mechanisms, the photocatalytic cycle involved a photo-induced electron transfer process including the photoexcitation of the catalyst (*i.e.*, 3) and the subsequent electron transfer to oxygen (Fig. 6d). Firstly, 3 was excited from its ground state to an excited state to generate electron–hole pairs whose electronic structure was optimized by DFT calculations. Subsequently, the electron was transferred to oxygen to generate a superoxide ion (˙O_2_^−^) and amine radical cations. Finally, the hole was transferred to the amine substrate (or we can say that the amine transferred one electron to the radical cation 3˙^+^), thus completing the photocatalytic redox cycle. To confirm this photocatalytic cycle, spin-trapping EPR spectra were recorded with 3 and benzylamine under visible-light irradiation. Based on the simulation results, the spin-trapping EPR experiment confirmed the formation of both ˙O_2_^−^ and amine radical cation using 5,5-dimethyl-1-pyrroline *N*-oxide (DMPO) as the trapping agent (Fig. 6a). It should be noted that the absence of singlet oxygen (^1^O_2_) based on the EPR spin-trapping analysis might exclude another potential reaction mechanism that the amine reacted with ^1^O_2_.^57^ Hydrogen peroxide was also conformed to be generated during this reaction process (Fig. 6b). In addition, a characteristic odor of NH_3_ was noticed during this oxidative coupling reaction, and the formation of NH_3_ was further confirmed by litmus paper (Fig. 6c). The controlled experiments also indicated that these intermediate species could only be detected when the reaction was performed under light irradiation. In the literature, several mechanisms of oxidative coupling of primary amines were reported, including the photoinduced generation of aldehydes and the successive condensation reaction that resulted in the coupled imines.^54,58–63^ However, the *in situ* NMR did not probe the formation of aldehyde species, thus excluding this reaction pathway (Fig. S71–S85†). Based on these results, a plausible reaction pathway for oxidative coupling reactions of amines in the presence of 3 is proposed in Fig. 6d. Notably, though several mechanisms of oxidative coupling of primary amines were reported in the literature, comprehensive studies on such reaction mechanisms are very rare.

## Conclusions

In summary, a series of *N*-aryl substituted extended phenothiazines 1–5 with up to seven linearly fused rings were successfully synthesized through a simple and versatile synthetic strategy. We demonstrated that the increased conjugation of phenothiazines not only resulted in their red-shifted absorption spectra but also a remarkable increase in the molar extinction coefficient, which is very beneficial for efficient visible-light photocatalytic processes when they are used as photocatalysts. Similar to conventional phenothiazines, the designed extended phenothiazines of 1–5 showed reversible redox behavior and maintained a strong excited-state reduction potential. Their radical cation species were studied by EPR spectroscopy and the results suggested that the properties of 3˙^+^, 4˙^+^ and 5˙^+^ were different from those of 1˙^+^ and 2˙^+^ with respect to their EPR profiles, spin density distributions and stability. A comparative study on the catalytic performance indicated that 3, 4 and 5 serving as photocatalysts could succeed in the visible-light-driven oxidative coupling reactions of amines, while 1 and 2, like the reference PTZ, can only catalyze such reactions in the presence of ultraviolet light. Notably, 3 displayed superior catalytic performance, especially in terms of stability and reaction time. Moreover, a direct and efficient conversion of amines to imines under sunlight in an air atmosphere was successfully realized, which further confirmed the exceptional catalytic performance of 3. Therefore, this work successfully demonstrated that the ring-fusion approach could efficiently tune the photophysical and redox properties of phenothiazines, and eventually significantly affect their catalytic performance. It is expected that our studies would not only provide a new synthetic strategy for chemical modification of phenothiazine-based materials but also shed some light on the understanding of the structure–property relationships of photocatalytic materials at the molecular level.

## Data availability

All experimental and characterization data as well as computational data are provided in the ESI.† Crystallographic data for all new compounds have been deposited at the CCDC under accession numbers CCDC 2145133 (1), 2145124 (1˙^+^), 2143328 (2), 2143327 (2˙^+^), 2143332 (3), 2143331 (4), 2143509 (5), 2143510 (a-OMe), 2150682 (a-OTf) and 2150683 (3-methoxynaphthalene-2-thiol).

## Author contributions

J. Z. synthesized the new compounds, and conducted the characterization, catalysis and most of the measurements. L. M. performed the theoretical calculations. M. W. aided with analysing and interpreting the data. X. Z. conducted the single crystal analysis. X. S. and H. Y. conceived the idea, directed this study and prepared the manuscript. Y. Y. and M. Z. helped to conduct the EPR measurements.

## Conflicts of interest

There are no conflicts to declare.

## Supplementary Material

SC-013-D2SC01086K-s001

SC-013-D2SC01086K-s002
