# Supplementary material for: Extended phenothiazines: synthesis, photophysical and redox properties, and efficient photocatalytic oxidative coupling of amines
Source: Chem Sci. 2022 Apr 8;13(18):5252–60. doi: 10.1039/d2sc01086k (PMC9093200; doi:10.1039/d2sc01086k)
Supplement: SC-013-D2SC01086K-s001 [file SC-013-D2SC01086K-s001.pdf]

Electronic Supplementary Material (ESI) for Chemical Science.
This journal is © The Royal Society of Chemistry 2022

Supporting Information

Extended Phenothiazines: Synthesis, Photophysical and Redox Properties, and Efficient Photocatalytic Oxidative Coupling of Amines

Jun Zhou, Lijun Mao, Meng-Xiang Wu, Zhiyong Peng, Yiming Yang, Manfei Zhou, Xiao-Li Zhao,* Xueliang Shi,* Hai-Bo Yang

Shanghai Key Laboratory of Green Chemistry and Chemical Processes, School of Chemistry and Molecular Engineering, Chang-Kung Chuang Institute, East China Normal University 3663 N. Zhongshan Road, Shanghai 200062, P. R. China

Email: xlzhao@chem.ecnu.edu.cn, xlshi@chem.ecnu.edu.cn

Contents

1 General 2
2 Synthesis and characterization 3
3 Theoretical calculation 11
 3.1 HOMO and LUMO energies 11
 3.2 Spin density and spin population 12
 3.3 The dihedral angle and bond length analysis 14
 3.4 Real space representation of hole and electron distributions 15
4 UV-vis-NIR titrations 16
5 EPR studies 16
6 Single crystal data 19
 6.1 Single crystals growth of 1-5 19
 6.2 Single crystals data 22
7 Photostability study 26
8 Comparison of catalytic performance of different catalysts 27
9 Sunlight-driven oxidative coupling of amines to imines 27
10 NMR spectra 28
 10.1 ^{1}H and ^{13}C NMR spectra of new compounds 28
 10.2 2D NMR spectra of new compounds 45
 10.3 In situ ^{1}H NMR spectra of imines 48
  10.3.1 In situ ^{1}H NMR spectra in Table 2 48
  10.3.2 In situ ^{1}H NMR spectra in Table 3 55
11 GC-MS data of imines 59
12 Mass spectra of new compounds 64
13 Reference 68

1 General

All solvents were dried according to the standard procedures and all of them were degassed under N_2 for 30 minutes before use. All air-sensitive reactions were carried out under inert N_2 atmosphere. The ^{1}H NMR, ^{13}C NMR, 2D NMR (NOESY, COSY) spectra were recorded on Bruker 300MHz, 400 MHz, 500 MHz spectrometer. The ^{1}H and ^{13}C NMR chemical shifts are reported relative to the residual solvent signals. Coupling constants (*J*) are denoted in Hz and chemical shifts (δ) are denoted in ppm. Multiplicities are denoted as follows: s = singlet, d = doublet and m = multiplet. UV-vis-NIR spectra were recorded in a quartz cell (light path 10 mm) on a Shimadzu UV2700 UV-visible spectrophotometer. Fluorescence spectra and photoluminescence quantum yields (Φ_F) were recorded on HORIBA Duetta. Cyclic voltammetry was recorded on a Bio-Logic SAS SP-150 spectrometer in anhydrous CH_2Cl_2 containing *n*-BuNPF_6 (0.1 M) as supporting electrolyte at a scan rate of 20 mV/s at room temperature. The CV cell has a glassy carbon electrode, a Pt wire counter electrode, and an Ag/Ag^+ reference electrode. The potential was calibrated against the ferrocene/ferrocenium (Fc/Fc^+) couple. $E^{\theta}_{Fc^+/Fc}$ = 0.46 V vs. SCE in CH_2Cl_2. $E_{1/2}$ ($PC^{\cdot+}$/PC^*) = $E_{1/2}$ ($PC^{\cdot+}$/PC) - E_{hv} where $E_{hv} = hc/\lambda_{max}$ = 1240 nm/λ_{max}. $E_{1/2}$ ($PC^{\cdot+}$/PC) was obtained from CV as the half sum of anodic (E_{pa}) and cathodic (E_{pc}) peak potentials, $E_{1/2}$ ($PC^{\cdot+}$/PC) = (E_{pa}+E_{pc})/2. E_g^{opt} was calculated from the onset of the absorption band. E_{ox} is the first oxidation potential, versus ferrocene/ferricenium (Fc/Fc^+). E_{HOMO} was calculated from the onset of the first oxidation wave and referenced against Fc/Fc^+ at -4.8 eV vs. vacuum. E_{LUMO} was calculated with E_{HOMO} and E_g^{opt}. EPR spectra for radicals were obtained on Bruker EMX instrument EMXPLUS-10/12. EPR spectra simulation was conducted on the Bruker SpinFit software. The HR-ESI mass spectra were performed on Q Exactive Focus (Thermo Scientific, USA). The single crystals were measured on Bruker Apex duo equipment with Cu radiation (λ = 1.54184 Å). DFT computations were performed by Gaussian 16 program. During the optimization, all atoms were described by RM06-2X/def2-SVP and UM06-2X/def2-SVP computational level for neutral compounds and radical cations, respectively. Frequency calculations were conducted to ensure that these structures were indeed local minima without imaginary frequencies. The single point energy was performed at the level of M06-2X/def2-TZVP. Time-dependent density functional theory (TD-DFT) calculations at the CAM-B3LYP/def2-SVP level. Electronic structure analyses were performed with the Multiwfn 3.8 (dev) code. The isosurface maps of various molecular orbitals, spin density and hole-electron distributions were rendered by Visual Molecular Dynamics (VMD 1.9.3) software based on the files exported from Multiwfn.[1-4] The photocatalytic experiments were carried out under photoreactor which was purchased from Shanghai 3S Technology Co., Ltd. (item no. S-Light-AL1). Yields of imines were determined by in situ ^{1}H NMR using 1,3,5-trimethoxybenzene as an internal standard. In order to increase the solubility of products, adding some DMSO to the mixture after reaction. Conversion was determined by gas chromatograph (GC-2014, SHIMADZU, equipped with an FID detector). Products were confirmed by ^{1}H NMR and GC-MS (GCMSQP2020, SHIMADZU).

2 Synthesis and characterization

Compound a-OMe

A round-bottom flask was charged with 2-bromo-3-methoxynaphthalene (3.54 g, 15.0 mmol), 2-methoxybenzenethiol (2.20 mL, 18.0 mmol), $Pd_2(dba)_3$ (686 mg, 0.75 mmol), XPhos (715 mg, 1.50 mmol), *t*-BuONa (4.32 g, 45.0 mmol) and toluene (100 mL). The mixture was stirred at 130 °C for 24 h under N_2 protection. After reaction, the resulting mixture was diluted with H_2O and extracted with AcOEt. The organic layer was dried over anhydrous Na_2SO_4, filtrated and evaporated under reduced pressure. The crude product was purified by column chromatography on silica gel (PE:EA = 20:1) to obtain the compound **a-OMe** (2.49 g) as a white solid with yield of 56%. ^{1}H NMR (500 MHz, CD_2Cl_2) δ (ppm) 7.78 (d, *J* = 8.2 Hz, 1H), 7.61 (d, *J* = 8.2 Hz, 1H), 7.47-7.42 (m, 2H), 7.39 (td, *J* = 8.3, 1.7 Hz, 1H), 7.36-7.32 (m, 1H), 7.31 (dd, *J* = 7.6, 1.6 Hz, 1H), 7.24 (s, 1H), 7.05-6.95 (m, 2H), 4.00 (s, 3H), 3.87 (s, 3H). ^{13}C NMR (125 MHz, CD_2Cl_2) δ (ppm) 158.98, 155.65, 133.98, 133.83, 129.80, 129.49, 129.20, 127.12, 126.80, 126.54, 126.41, 124.37, 121.70, 121.64, 111.63, 105.83, 56.22, 56.19.

Compound a-OTf

Compound **a-OMe** (1.00 g, 3.40 mmol) was dissolved in DCM (20 mL). BBr_3 (2.30 mL, 23.60 mmol) was added at 0 °C, and then the resulting mixture was stirred at room temperature for 1 h. The reaction mixture was quenched with MeOH (5 mL) dropwise at 0 °C. The resulting mixture was evaporated under reduced pressure to obtain crude product without purification. Then the crude product was dissolved in DCM (20 mL), pyridine (655 μL, 8.10 mmol) was added. Then triflic anhydride (1.70 mL, 10.1 mmol) was added at 0 °C, and the resulting mixture was stirred at room temperature for 2 h. After the reaction, the mixture was quenched with saturated $NaHCO_3$ aqueous solution and extracted with DCM (20 mL). The organic layer was dried over anhydrous Na_2SO_4, filtrated and evaporated under reduced pressure. The crude product was purified by column chromatography on silica gel (PE:DCM = 5:1) to give the compound **a-OTf** (1.39 g) as a white solid in 77% yield. ^{1}H NMR (500 MHz, $CDCl_3$) δ (ppm) 7.96 (s, 1H), 7.87 (d, *J* = 7.8 Hz, 1H), 7.85 (s, 1H), 7.79 (d, *J* = 7.7 Hz, 1H), 7.63-7.55 (m, 2H), 7.39-7.37 (m, 2H), 7.30-7.27 (m, 2H). ^{13}C NMR (125 MHz, $CDCl_3$) δ (ppm) 148.49, 146.50, 135.72, 133.54, 133.28, 132.70, 129.67, 129.18, 128.97, 128.47, 128.11, 128.06, 127.81, 124.76, 122.38, 120.48.

Compound 1

A round-bottom flask was charged with **a-OTf** (532 mg, 1.00 mmol), Cs_2CO_3 (977 mg, 3.00 mmol), DPEPhos (53.8 mg, 0.10 mmol), $Pd_2(dba)_3$ (45.8 mg, 0.05 mmol) and toluene (10 mL). 4-*tert*-butylaniline (175 μL, 1.10 mmol) was added to the mixture. The mixture was stirred at 130 °C for 24 h under N_2 protection. After reaction, the resulting mixture was diluted with H_2O and extracted with AcOEt. The organic layer was dried over anhydrous Na_2SO_4, filtrated and evaporated under reduced pressure. The crude product was purified by column chromatography on silica gel (PE:EA = 100:1) to obtain the compound **1** (186 mg) as a pale yellow solid with the yield of 49%. ^1H NMR (500 MHz, CD_2Cl_2) δ (ppm) 7.69 (d, *J* = 8.3 Hz, 2H), 7.54 (d, *J* = 7.5 Hz, 1H), 7.46 (s, 1H), 7.36-7.32 (m, 3H), 7.23-7.17 (m, 2H), 7.05 (d, *J* = 7.5 Hz, 1H), 6.86 (t, *J* = 7.4 Hz, 1H), 6.80 (t, *J* = 7.4 Hz, 1H), 6.44 (s, 1H), 6.21 (d, *J* = 8.2 Hz, 1H), 1.45 (s, 9H). ^{13}C NMR (125 MHz, CD_2Cl_2) δ (ppm) 155.64, 137.71, 137.53, 137.49, 137.29, 135.12, 135.09, 134.65, 133.38, 130.69, 130.57, 130.53, 129.07, 128.75, 128.44, 126.65, 126.57, 126.33, 126.14, 125.89, 124.93, 102.41, 55.70, 21.41, 21.20, 20.14, 19.90. HR-ESI-MS: *m/z* calculated for $C_{26}H_{24}NS$ $[M+H]^+$: 382.1551, found: 382.1616.

Compound b-OMe

A round-bottom flask was charged with 2-bromo-3-methoxynaphthalene (1.19g, 5.00 mmol), thiourea (247 mg, 3.25 mmol), Cs_2CO_3 (2.28 g, 7.00 mmol), $Pd_2(dba)_3$ (91.6 mg, 0.10 mmol), TriPhos (187 mg, 0.30 mmol) and 1,4-dioxane (50 mL). The mixture was stirred at 110 °C for 24 h under N_2 protection. After reaction, the resulting mixture was diluted with H_2O and extracted with AcOEt. The organic layer was dried over anhydrous Na_2SO_4, filtrated and evaporated under reduced pressure. The crude product was purified by column chromatography on silica gel (PE:EA = 10:1) to give the compound **b-OMe** (485 mg) as a white solid in 56% yield. ^1H NMR (500 MHz, CD_2Cl_2) δ (ppm) 7.78 (d, *J* = 8.2 Hz, 2H), 7.59 (d, *J* = 8.1 Hz, 2H), 7.56 (s, 2H), 7.44 (t, *J* = 7.6 Hz, 2H), 7.31 (t, *J* = 7.5 Hz, 2H), 7.25 (s, 2H), 3.96 (s, 6H). ^{13}C NMR (125 MHz, CD_2Cl_2) δ (ppm) 156.05, 134.38, 131.43, 131.39, 129.51, 127.37, 126.81, 125.15, 124.43, 106.12, 56.40. HR-ESI-MS: *m/z* calculated for $C_{22}H_{19}O_2S$ $[M+H]^+$: 347.1028, found: 347.1091.

Compound b-OTf

Compound **b-OMe** (346 mg, 1.00 mmol) was dissolved in DCM (5 mL). BBr_3 (674 µL, 7.00 mmol) was added at 0 °C, and then the resulting mixture was stirred at room temperature for 1 h. The reaction mixture was quenched with MeOH (5 mL) dropwise at 0 °C. The resulting mixture was evaporated under reduced pressure to obtain crude product without purification. The crude product was dissolved in DCM (5 mL). Then pyridine (194 µL, 2.40 mmol) was added. Then triflic anhydride (505 µL, 3.00 mmol) was added at 0 °C. and the resulting mixture was stirred at room temperature for 2 h. After the reaction, the mixture was quenched with saturated $NaHCO_3$ aqueous solution and extracted with DCM (10 mL). The organic layer was dried over anhydrous Na_2SO_4, filtrated and evaporated under reduced pressure. The crude product was purified by column chromatography on silica gel (PE:DCM = 5:1) to give the compound **b-OTf** (483 mg) as a white solid with yield of 83%. ^1H NMR (500 MHz, DMSO-d_6) δ (ppm) 8.27 (s, 2H), 8.15 (d, J = 8.0 Hz, 2H), 8.07 (s, 2H), 7.97 (d, J = 8.0 Hz, 2H), 7.66 (dt, J = 14.8, 7.0 Hz, 4H). ^{13}C NMR (125 MHz, DMSO-d_6) δ (ppm) 144.93, 133.96, 132.67, 132.40, 128.48, 128.36, 128.19, 127.72, 123.84, 120.83. HR-ESI-MS: *m/z* calculated for $C_{22}H_{13}O_6F_6S_3$ $[M+H]^+$: 582.9700, found: 582.9761.

Compound 2

A round-bottom flask was charged with **b-OTf** (583 mg, 1.00 mmol), Cs_2CO_3 (977 mg, 3.00 mmol), DPEPhos (53.8 mg, 0.10 mmol), $Pd_2(dba)_3$ (45.8 mg, 0.0500 mmol) and toluene (10 mL). 4-*tert*-butylaniline (175 µL, 1.10 mmol) was added to the mixture. The mixture was stirred at 130 °C for 24 h under N_2 protection. After reaction, the resulting mixture was diluted with H_2O and extracted with AcOEt. The organic layer was dried over anhydrous Na_2SO_4, filtrated and evaporated under reduced pressure. The crude product was purified by column chromatography on silica gel (PE:EA = 100:1) to give the compound **2** (155 mg) as a white solid in 36% yield. ^1H NMR (500 MHz, CD_2Cl_2) δ (ppm) 7.75 (d, J = 8.3 Hz, 2H), 7.60-7.54 (m, 4H), 7.42 (d, J = 8.3 Hz, 2H), 7.35 (d, J = 7.4 Hz, 2H), 7.27-7.16 (m, 4H), 6.50 (s, 2H), 1.48 (s, 9H). ^{13}C NMR (125 MHz, CD_2Cl_2) δ (ppm) 152.35, 141.60, 138.57, 133.50, 130.52, 130.05, 128.53, 127.12, 126.50, 126.36, 124.59, 124.57, 122.03, 111.82, 35.24, 31.62. HR-ESI-MS: *m/z* calculated for $C_{30}H_{26}NS$ $[M+H]^+$: 432.1708, found: 432.1775.

Compound 2-Br

9,10-dimesityl-2-methoxyanthracene was synthesized by previous reported procedures.[4] 9,10-Dimesityl-2-methoxyanthracene (889 mg, 2.00 mmol) was dissolved in THF 20 mL. Then ethyl *n*-BuLi (0.96 mL, 2.40 mmol) was added at -78 °C under N_2 protection and the resulting mixture was stirred at room temperature for 1 h. After that, 1,2-dibromoethane (190 μL, 2.20 mmol) was added to the mixture at -78 °C. The reaction mixture was stirred at room temperature overnight. After that, the mixture was quenched with saturated NH_4Cl aqueous solution and extracted with AcOEt. The organic layer dried over anhydrous Na_2SO_4, filtrated and evaporated under reduced pressure. The crude product was purified by column chromatography on silica gel (PE) to obtain the compound **2-Br** (846 mg) as a yellow solid in 81% yield. ^{1}H NMR (500 MHz, $CDCl_3$) δ (ppm) 7.77 (s, 1H), 7.46 (dd, *J* = 13.4, 8.5 Hz, 2H), 7.33-7.24 (m, 2H), 7.12 (s, 4H), 6.75 (s, 1H), 3.74 (s, 3H), 2.48 (s, 3H), 2.47 (s, 3H), 1.79 (s, 6H), 1.78 (s, 6H). ^{13}C NMR (125 MHz, $CDCl_3$) δ(ppm) 153.09, 137.61, 137.56, 137.45, 137.30, 134.84, 134.66, 134.36, 133.60, 130.93, 130.46, 129.81, 129.08, 128.57, 126.92, 126.62, 126.10, 125.92, 125.07, 115.22, 103.60, 56.18, 21.43, 21.41, 20.11, 19.98. HR-ESI-MS: *m/z* calculated for $C_{33}H_{32}OBr$ $[M+H]^+$: 523.1558, found: 523.1634.

Compound c-OMe

A round-bottom flask was charged with **2-Br** (1.00 g, 2.00 mmol), 2-methoxybenzenethiol (292 μL, 2.40 mmol), $Pd_2(dba)_3$ (91.57 mg, 0.10 mmol), XPhos (95.34 mg, 0.2 mmol), *t*-BuONa (577 mg, 6.00 mmol) and toluene (20 mL). The mixture was stirred at 130 °C for 24 h under N_2 protection. After reaction, the resulting mixture was diluted with H_2O and extracted with AcOEt. The organic layer was dried over anhydrous Na_2SO_4, filtrated and evaporated under reduced pressure. The crude product was purified by column chromatography on silica gel (PE:EA = 100:1) to give the compound **c-OMe** (699 mg) as a yellow solid with yield of 60%.^{1}H NMR (500 MHz, DMSO-d_6) δ (ppm) 7.41 (t, *J* = 7.9 Hz, 1H), 7.34-7.22 (m, 5H), 7.16 (s, 2H), 6.95-6.85 (m, 4H), 6.58 (s, 1H), 6.32 (s, 1H), 3.66 (s, 3H), 3.63 (s, 3H), 2.42 (s, 3H), 2.40 (s, 3H), 1.70 (s, 6H), 1.48 (s, 6H). ^{13}C NMR (125 MHz, DMSO-d_6) δ (ppm) 159.62, 153.75, 136.66, 136.61, 136.49, 136.24, 136.01, 134.14, 133.51, 132.98, 132.23, 131.42, 129.64, 128.84, 128.39, 127.95, 125.59, 125.54, 125.43, 125.20, 124.88, 121.97, 121.33, 116.46, 111.72, 100.77, 55.47, 55.31, 20.91, 20.89, 19.44, 19.25. HR-ESI-MS: *m/z* calculated for $C_{40}H_{39}O_2S$ $[M+H]^+$: 583.2593, found: 583.2658.

Compound c-OTf

Compound **c-OMe** (582 mg, 1.00 mmol) was dissolved in DCM (10 mL). BBr_3 (674 μL, 7.00 mmol) was added at 0 °C, and the resulting mixture was stirred at room temperature for 1 h. The reaction mixture was quenched with MeOH (3 mL) dropwise at 0 °C. The resulting mixture was evaporated under reduced pressure to obtain crude product without purification. The crude product was dissolved in DCM (10 mL). Then pyridine (194 μL, 2.40 mmol) was added. Then triflic anhydride (505 μL, 3.00 mmol) was added at 0 °C. and the resulting mixture was stirred at room temperature for 2 h. After that, the mixture was quenched with saturated $NaHCO_3$ aqueous solution and extracted with DCM (10 mL). The organic layer was dried over anhydrous Na_2SO_4, filtrated and evaporated under reduced pressure. The crude product was purified by column chromatography on silica gel (PE:EA = 60:1) to give the compound **c-OTf** (622 mg) as a yellow solid with yield of 76%. ^1H NMR (500 MHz, CD_2Cl_2) δ (ppm) 7.56-7.51 (m, 2H), 7.47 (s, 1H), 7.45-7.34 (m, 4H), 7.31-7.28 (m, 2H), 7.20 (s, 1H), 7.15 (s, 2H), 7.00 (s, 2H), 2.47 (s, 3H), 2.43 (s, 3H), 1.76 (s, 6H), 1.64 (s, 6H). ^{13}C NMR (125 MHz, CD_2Cl_2) δ (ppm) 149.65, 145.66, 138.44, 137.94, 137.70, 137.51, 136.93, 136.17, 135.47, 133.56, 133.51, 131.62, 131.27, 131.19, 130.80, 129.67, 128.84, 128.71, 128.66, 127.28, 127.09, 127.07, 126.95, 126.84, 125.92, 122.67, 118.93, 21.39, 21.35, 19.96, 19.93. HR-ESI-MS: *m/z* calculated for $C_{40}H_{33}O_6F_6S_3$ $[M+H]^+$: 819.1265, found: 819.1315.

Compound 3

A round-bottom flask was charged with **c-OTf** (982 mg, 1.20 mmol), $Pd_2(dba)_3$ (55.0 mg, 0.06 mmol), DPEPhos (64.6 mg, 0.12 mmol), Cs_2CO_3 (1.20 g, 3.60 mmol) and toluene (20 mL). 4-*tert*-butylaniline (214 μL, 1.30 mmol) was added to the mixture. The mixture was stirred at 130 °C for 24 h under N_2 protection. After that, the resulting mixture was diluted with H_2O and extracted with AcOEt. The organic layer was dried over anhydrous Na_2SO_4, filtrated and evaporated under reduced pressure. The crude product was purified by column chromatography on silica gel (PE:DCM = 10:1) to give the compound **3** (480 mg) as a yellow solid with yield of 60%. ^1H NMR (400 MHz, CD_2Cl_2) δ (ppm) 7.46 (d, J = 8.4 Hz, 2H), 7.36-7.27 (m, 1H), 7.21-7.07 (m, 8H), 6.99 (d, J = 7.5 Hz, 1H), 6.87 (s, 2H), 6.81 (t, J = 7.7 Hz, 1H), 6.74 (t, J = 7.1 Hz, 1H), 6.15 (d, J = 8.1 Hz, 1H), 6.11 (s, 1H), 2.46 (s, 3H), 2.34 (s, 3H), 1.77 (s, 6H), 1.60 (s, 6H), 1.39 (s, 9H). ^{13}C NMR (100 MHz, CD_2Cl_2) δ (ppm) 155.64, 137.71, 137.53, 137.49, 137.29, 135.12, 135.09, 134.65, 133.38, 130.69, 130.57, 130.53, 129.07, 128.75, 128.44, 126.65, 126.57, 126.33, 126.14, 125.89, 124.93, 102.41, 55.70, 21.41, 21.20, 20.14, 19.90. HR-ESI-MS: *m/z* calculated for $C_{48}H_{46}NS$ $[M+H]^+$: 668.3273, found: 668.3343.

Compound 3-methoxynaphthalene-2-thiol

2-Methoxynaphthalene (5.00 g, 31.6 mmol) was dissolved in THF (100 mL). Then ethyl *n*-BuLi (18.2 mL, 45.5 mmol) was added at -78 ^oC and the resulting mixture was stirred at room temperature for 1 h. After that, elemental sulfur (1.21 g, 37.9 mmol) was added to the mixture at -78 ^oC. Then the reaction mixture stirred at room temperature overnight. After the reaction, the mixture was quenched with saturated NH_4Cl aqueous solution and extracted with AcOEt. The organic layer dried over anhydrous Na_2SO_4, filtrated and evaporated under reduced pressure. The crude product was purified by column chromatography on silica gel (PE) to give the compound **3-methoxynaphthalene-2-thiol** (2.90 g) as a white solid in 49% yield. ^1H NMR (500 MHz, DMSO-d_6) δ (ppm) 7.86 (s, 1H), 7.77 (d, J = 8.1 Hz, 1H), 7.68 (d, J = 8.0 Hz, 1H), 7.41-7.27 (m, 3H), 5.11 (s, 1H), 3.94 (s, 3H). ^{13}C NMR (125 MHz, DMSO-d_6) δ (ppm) 153.31, 132.19, 128.61, 126.56, 126.52, 125.98, 125.40, 124.14, 123.50, 105.58, 55.95.

Compound d-OMe

A round-bottom flask was charged with **2-Br** (2.1 g, 4.0 mmol), **3-methoxynaphthalene-2-thiol** (912 mg, 4.80 mmol), $Pd_2(dba)_3$ (183 mg, 0.20 mmol), XPhos (191 mg, 0.40 mmol), *t*-BuONa (1.15 g, 12.0 mmol) and toluene (30 mL). The mixture was stirred at 130 ^oC for 24 h under N_2 protection. After reaction, the resulting mixture was diluted with H_2O and extracted with AcOEt. The organic layer was dried over anhydrous Na_2SO_4, filtrated and evaporated under reduced pressure. The crude product was purified by column chromatography on silica gel (PE:EA = 100 : 1) to obtain the compound **d-OMe** (1.24 g) as a yellow solid with yield of 49%. ^1H NMR (500 MHz, DMSO-d_6) δ (ppm) 7.95 (s, 1H), 7.90 (d, J = 8.3 Hz, 1H), 7.76 (d, J = 8.1 Hz, 1H), 7.59 (t, J = 7.5 Hz, 1H), 7.41 (t, J = 7.5 Hz, 1H), 7.34-7.21 (m, 5H), 7.16 (s, 2H), 6.61 (s, 1H), 6.54 (s, 2H), 6.40 (s, 1H), 3.72 (s, 3H), 3.69 (s, 3H), 2.42 (s, 3H), 2.15 (s, 3H), 1.71 (s, 6H), 1.38 (s, 6H). ^{13}C NMR (125 MHz, DMSO-d_6) δ (ppm) 156.20, 153.75, 136.68, 136.60, 136.39, 136.34, 135.76, 135.07, 134.10, 133.26, 133.05, 132.25, 130.20, 128.89, 128.51, 128.40, 127.93, 127.54, 127.28, 127.25, 126.68, 125.56, 125.50, 125.46, 125.17, 124.87, 124.03, 122.66, 119.51, 106.61, 100.82, 55.53, 26.35, 20.90, 20.85, 19.43, 19.17. HR-ESI-MS: *m/z* calculated for $C_{44}H_{41}O_2S$ [M+H]$^+$: 633.2749, found: 633.2812.

Compound d-OTf

Compound **d-OMe** (1.07 g, 1.70 mmol) was dissolved in DCM (20 mL). BBr_3 (1.16 mL, 12.0 mmol) was added at 0 °C, and then the resulting mixture was stirred at room temperature for 1 h. The reaction mixture was quenched with MeOH (10 mL) dropwise at 0 °C and evaporated under reduced pressure to obtain crude product without purification. The crude product was dissolved in DCM (20 mL). Then pyridine (330 μL, 4.08 mmol) was added. Triflic anhydride (858 μL, 5.10 mmol) was added at 0 °C, and the resulting mixture was stirred at room temperature for 2 h. After that, the mixture was quenched with saturated $NaHCO_3$ aqueous solution and extracted with DCM. The organic layer was dried over anhydrous Na_2SO_4, filtrated and evaporated under reduced pressure. The crude product was purified by column chromatography on silica gel (PE:EA = 100:1) to give the compound **d-OTf** (900 mg) as a yellow solid with yield of 61%. ^1H NMR (500 MHz, CD_2Cl_2) δ (ppm) 8.02 (s, 1H), 7.94 (d, J = 8.2 Hz, 1H), 7.80-7.74 (m, 2H), 7.71 (t, J = 7.5 Hz, 1H), 7.65 (t, J = 7.5 Hz, 1H), 7.51 (dd, J = 7.4, 1.8 Hz, 1H), 7.47-7.43 (m, 2H), 7.34 (dqd, J = 7.8, 6.4, 1.5 Hz, 2H), 7.14 (s, 2H), 7.03 (s, 1H), 6.64 (s, 2H), 2.46 (s, 3H), 2.18 (s, 3H), 1.75 (s, 6H), 1.53 (s, 6H). ^{13}C NMR (125 MHz, CD_2Cl_2) δ (ppm) 155.64, 137.71, 137.53, 137.49, 137.29, 135.12, 135.09, 134.65, 133.38, 130.69, 130.57, 130.53, 129.07, 128.75, 128.44, 126.65, 126.57, 126.33, 126.14, 125.89, 124.93, 102.41, 55.70, 21.41, 21.20, 20.14, 19.90. HR-ESI-MS: *m/z* calculated for $C_{44}H_{35}F_6O_6S_3$ $[M+H]^+$: 869.1422, found: 869.1484.

Compound 4

A round-bottom flask was charged with **d-OTf** (521 mg, 0.60 mmol), $Pd_2(dba)_3$ (27.5 mg, 0.03 mmol), DPEPhos (32.3 mg, 0.06 mmol), Cs_2CO_3 (587 mg, 1.80 mmol) and toluene (10 mL). 4-*tert*-butylaniline (105 μL, 0.66 mmol) was added to the mixture. The mixture was stirred at 130 °C for 24 h under N_2 protection. After reaction, the resulting mixture was diluted with H_2O and extracted with AcOEt. The organic layer was dried over anhydrous Na_2SO_4, filtrated and evaporated under reduced pressure. The crude product was purified by column chromatography on silica gel (PE:DCM = 10:1) to give the compound **4** (211 mg) as a yellow solid with yield of 49%. ^1H NMR (400 MHz, CD_2Cl_2) δ (ppm) 7.57-7.48 (m, 4H), 7.36-7.29 (m, 2H), 7.24-7.10 (m, 10H), 6.89 (s, 2H), 6.46 (s, 1H), 6.22 (s, 1H), 2.48 (s, 3H), 2.36 (s, 3H), 1.78 (s, 6H), 1.61 (s, 6H), 1.43 (s, 9H). ^{13}C NMR (100 MHz, CD_2Cl_2) δ (ppm) 155.64, 137.71, 137.53, 137.49, 137.29, 135.12, 135.09, 134.65, 133.38, 130.69, 130.57, 130.53, 129.07, 128.75, 128.44, 126.65, 126.57, 126.33, 126.14, 125.89, 124.93, 102.41, 55.70, 21.41, 21.20, 20.14, 19.90. HR-ESI-MS: *m/z* calculated for $C_{52}H_{48}NS$ $[M+H]^+$: 718.3429, found: 718.3474.

Compound e-OMe

A round-bottom flask was charged with **2-Br** (2.60 g, 5.00 mmol), thiourea (251 mg, 3.30 mmol), $Pd_2(dba)_3$ (91.6 mg, 0.10 mmol), TriPhos (187 mg, 0.30 mmol) and Cs_2CO_3 (2.30 g, 7.00 mmol), 1,4-dioxane (40 mL). The mixture was stirred at 110 °C for 24 h under N_2 protection. After reaction, the resulting mixture was diluted with H_2O and extracted with AcOEt. The organic layer was dried over anhydrous Na_2SO_4, filtrated and evaporated under reduced pressure. The crude product was purified by column chromatography on silica gel (PE:EA = 80 : 1) to give the compound **e-OMe** (1.35 g) as a yellow solid with yield of 59%. ^{1}H NMR (500 MHz, CD_2Cl_2) δ (ppm) 7.40 (d, *J* = 8.6 Hz, 2H), 7.34-7.33 (m, 4H), 7.31-7.25 (m, 2H), 7.23-7.19 (m, 2H), 7.15 (s, 4H), 6.89 (s, 4H), 6.65 (s, 2H), 3.55 (s, 6H), 2.46 (s, 6H), 2.20 (s, 6H), 1.82 (s, 12H), 1.60 (s, 12H). ^{13}C NMR (125 MHz, CD_2Cl_2) δ (ppm) 155.23, 137.30, 137.12, 137.08, 136.88, 134.71, 134.68, 134.24, 132.96, 130.28, 130.16, 130.12, 128.66, 128.34, 128.03, 126.24, 126.15, 125.92, 125.73, 125.48, 124.52, 102.00, 55.28, 21.00, 20.79, 19.73, 19.49. HR-ESI-MS: *m/z* calculated for $C_{66}H_{63}O_2S$ $[M+H]^+$: 919.4471, found: 919.4523.

Compound e-OTf

Compound **e-OMe** (1.45 g, 1.58 mmol) was dissolved in DCM (20 mL). BBr_3 (1.07 mL, 11.0 mmol) was added at 0 °C, and then the resulting mixture was stirred at room temperature for 1 h. The reaction mixture was quenched with MeOH (5 mL) dropwise at 0 °C and evaporated under reduced pressure to obtain crude product without purification. The crude product was dissolved in DCM (20 mL). Then pyridine (307 μL, 3.80 mmol) was added. Triflic anhydride (791 μL, 4.70 mmol) was added at 0 °C, and the resulting mixture was stirred at room temperature for 2 h. After that, the mixture was quenched with saturated $NaHCO_3$ aqueous solution and extracted with DCM. The organic layer was dried over anhydrous Na_2SO_4, filtrated and evaporated under reduced pressure. The crude product was purified by column chromatography on silica gel (PE:EA = 100:1) to give the compound **d-OTf** (1.28 g) as a yellow solid with yield of 70%. ^{1}H NMR (500 MHz, CD_2Cl_2) δ (ppm) 7.65 (s, 2H), 7.53 (d, *J* = 7.9 Hz, 2H), 7.46 (d, *J* = 7.6 Hz, 2H), 7.44-7.32 (m, 6H), 7.15 (s, 4H), 6.93 (s, 4H), 2.46 (s, 6H), 2.22 (s, 6H), 1.77 (s, 12H), 1.59 (s, 12H). ^{13}C NMR (125 MHz, CD_2Cl_2) δ (ppm) 155.64, 137.71, 137.53, 137.49, 137.29, 135.12, 135.09, 134.65, 133.38, 130.69, 130.57, 130.53, 129.07, 128.75, 128.44, 126.65, 126.57, 126.33, 126.14, 125.89, 124.93, 102.41, 55.70, 21.41, 21.20, 20.14, 19.90. HR-ESI-MS: *m/z* calculated for $C_{66}H_{57}F_6O_6S_3$ $[M+H]^+$: 1155.3143, found: 1155.3214.

Compound 5

A round-bottom flask was charged with **e-OTf** (1.00 g, 0.87 mmol), $Pd_2(dba)_3$ (40.0 mg, 0.04 mmol), DPEPhos (48.5 mg, 0.09 mmol), Cs_2CO_3 (847 mg, 2.60 mmol), and toluene (20 mL). 4-*tert*-butylaniline (152 μL, 0.95 mmol) was added to the mixture. The mixture was stirred at 130 °C for 24 h under N_2 protection. After reaction, the resulting mixture was diluted with H_2O and extracted with AcOEt. The organic layer was dried over anhydrous Na_2SO_4, filtrated and evaporated under reduced pressure. The crude product was purified by column chromatography on silica gel (PE:DCM = 10:1) to give the compound **5** (210 mg) as a yellow solid with yield of 48%. ^{1}H NMR (500 MHz, CD_2Cl_2) δ (ppm) 7.34-7.27 (m, 4H), 7.22-7.18 (m, 4H), 7.17-7.13 (m, 4H), 7.10 (s, 4H), 6.97 (d, *J* = 8.3 Hz, 2H), 6.85 (s, 4H), 6.20 (s, 2H), 2.45 (s, 6H), 2.33 (s, 6H), 1.74 (s, 12H), 1.57 (s, 12H), 1.38 (s, 9H). ^{13}C NMR (125 MHz, CD_2Cl_2) δ (ppm) 140.30, 137.78, 137.64, 137.28, 136.76, 134.72, 134.45, 133.87, 133.69, 130.53, 129.87, 129.19, 129.13, 128.65, 128.23, 127.32, 126.92, 126.53, 126.15, 125.55, 124.90, 123.35, 122.55, 109.41, 34.84, 31.62, 21.52, 21.38, 20.03, 19.74. HR-ESI-MS: *m/z* calculated for $C_{74}H_{70}NS$ $[M+H]^+$: 1004.5151, found: 1004.5208.

3 Theoretical calculation

3.1 HOMO and LUMO energies

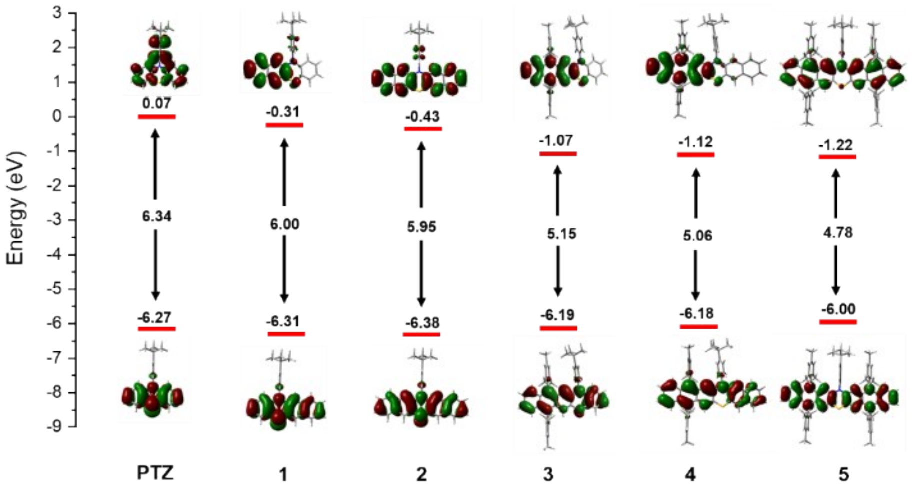

Figure S1. HOMO and LUMO energies of PTZ**, 1**, **2**, **3**, **4** and **5** at the M06-2X/def2-TZVP level of theory.

3.2 Spin density and spin population

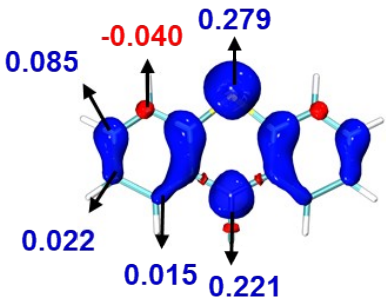

Figure S2. Spin density and Mulliken atomic spin density values of PTZ$^{•+}$ at the M06-2X/def2-TZVP level of theory.

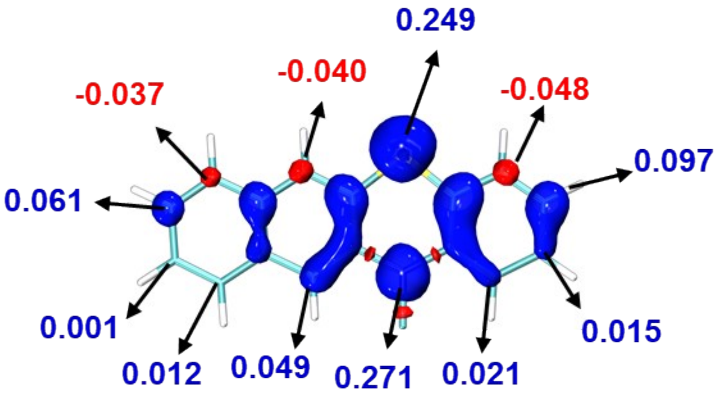

Figure S3. Spin density and Mulliken atomic spin density values of **1**$^{•+}$ at the M06-2X/def2-TZVP level of theory.

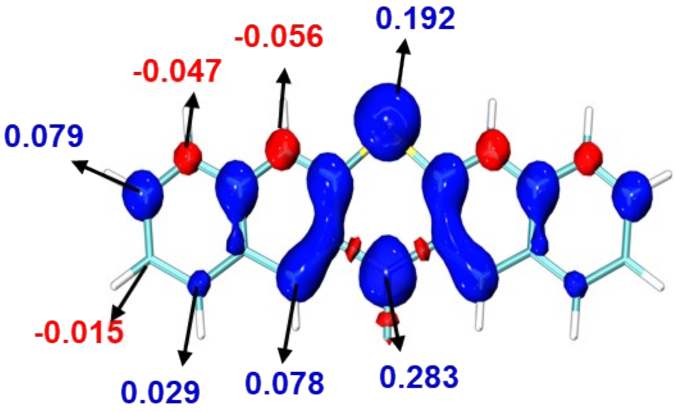

Figure S4. Spin density and Mulliken atomic spin density values of **2**$^{•+}$ at the M06-2X/def2-TZVP level of theory.

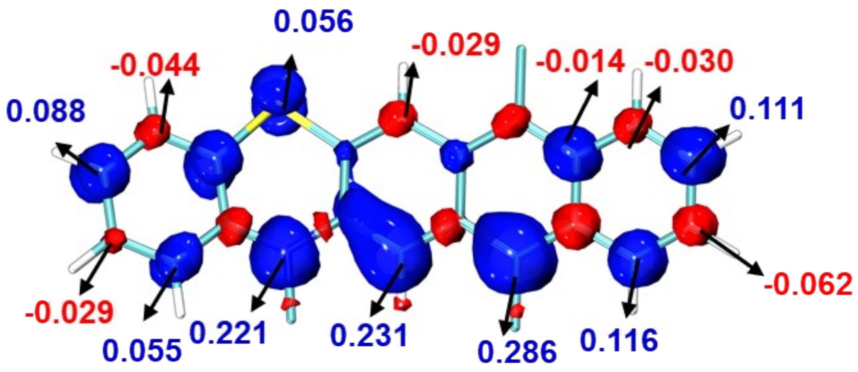

Figure S5. Spin density and Mulliken atomic spin density values of **3**$^{\bullet+}$ at the M06-2X/def2-TZVP level of theory.

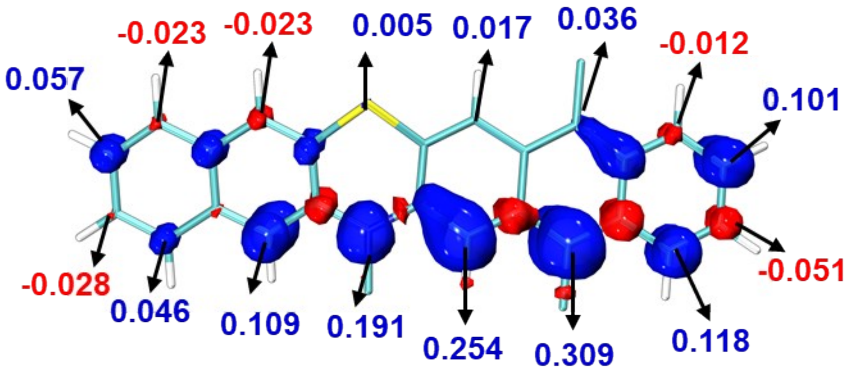

Figure S6. Spin density and Mulliken atomic spin density values of **4**$^{\bullet+}$ at the M06-2X/def2-TZVP level of theory.

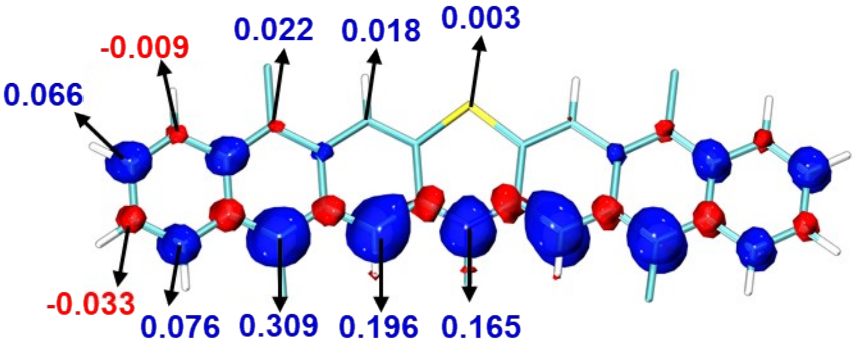

Figure S7. Spin density and Mulliken atomic spin density values of **5**$^{\bullet+}$ at the M06-2X/def2-TZVP level of theory.

3.3 The dihedral angle and bond length analysis

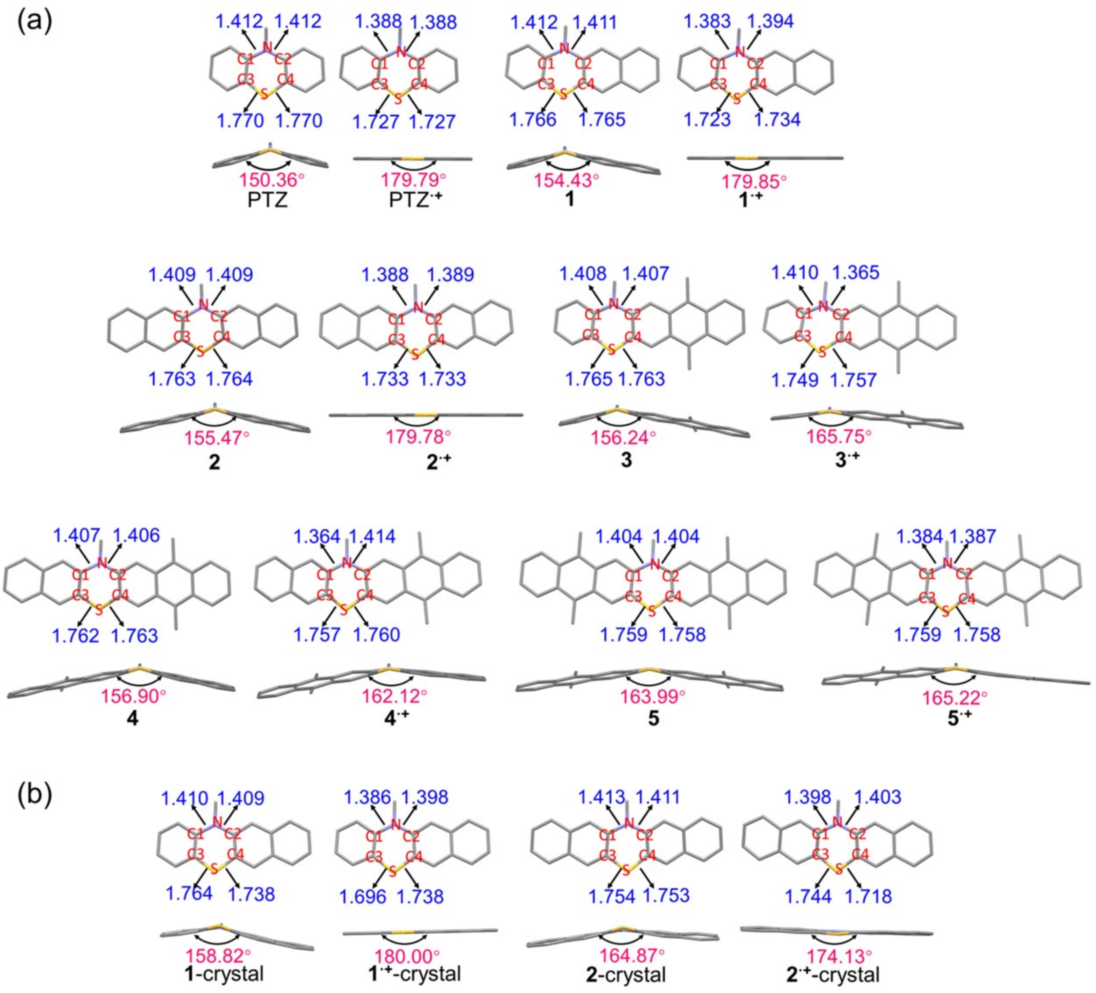

Figure S8. The dihedral angle and the bond length of DFT-optimized structure of PTZ, PTZ$^{•+}$, **1**, **1**$^{•+}$, **2**, **2**$^{•+}$, **3**, **3**$^{•+}$, **4**, **4**$^{•+}$, **5** and **5**$^{•+}$ at the M06-2X/def2-SVP level of theory (a) and the dihedral angle and the bond length of single crystal structure of **1**, **1**$^{•+}$, **2** and **2**$^{•+}$ (b).

Table S1. Dihedral angle and selected bond length of DFT-optimized structure of PTZ, PTZ$^{•+}$, **1**, **1**$^{•+}$, **2**, **2**$^{•+}$, **3**, **3**$^{•+}$, **4**, **4**$^{•+}$, **5** and **5**$^{•+}$ and dihedral angle and selected bond length of crystal structure of **1**, **1**$^{•+}$, **2** and **2**$^{•+}$.

	PTZ	PTZ$^{•+}$	1	1$^{•+}$	2	2$^{•+}$	3	3$^{•+}$	4	4$^{•+}$	5	5$^{•+}$	1 (crystal)	1$^{•+}$ (crystal)	2 (crystal)	2$^{•+}$ (crystal)
C(1)-N (Å)	1.412	1.388	1.412	1.383	1.409	1.388	1.408	1.410	1.407	1.364	1.404	1.384	1.410	1.386	1.413	1.398
C(2)-N (Å)	1.412	1.388	1.411	1.394	1.409	1.389	1.407	1.365	1.406	1.414	1.404	1.387	1.409	1.398	1.411	1.403
C(3)-S (Å)	1.770	1.727	1.766	1.723	1.763	1.733	1.765	1.749	1.762	1.757	1.759	1.759	1.764	1.696	1.754	1.744
C(4)-S (Å)	1.770	1.727	1.765	1.734	1.764	1.733	1.763	1.757	1.763	1.760	1.758	1.758	1.738	1.738	1.753	1.718
Dihedral angle (°)	150.36	179.79	154.43	179.85	155.47	179.78	156.24	165.75	156.90	162.12	163.99	165.22	158.82	180.00	164.87	174.13

3.4 Real space representation of hole and electron distributions

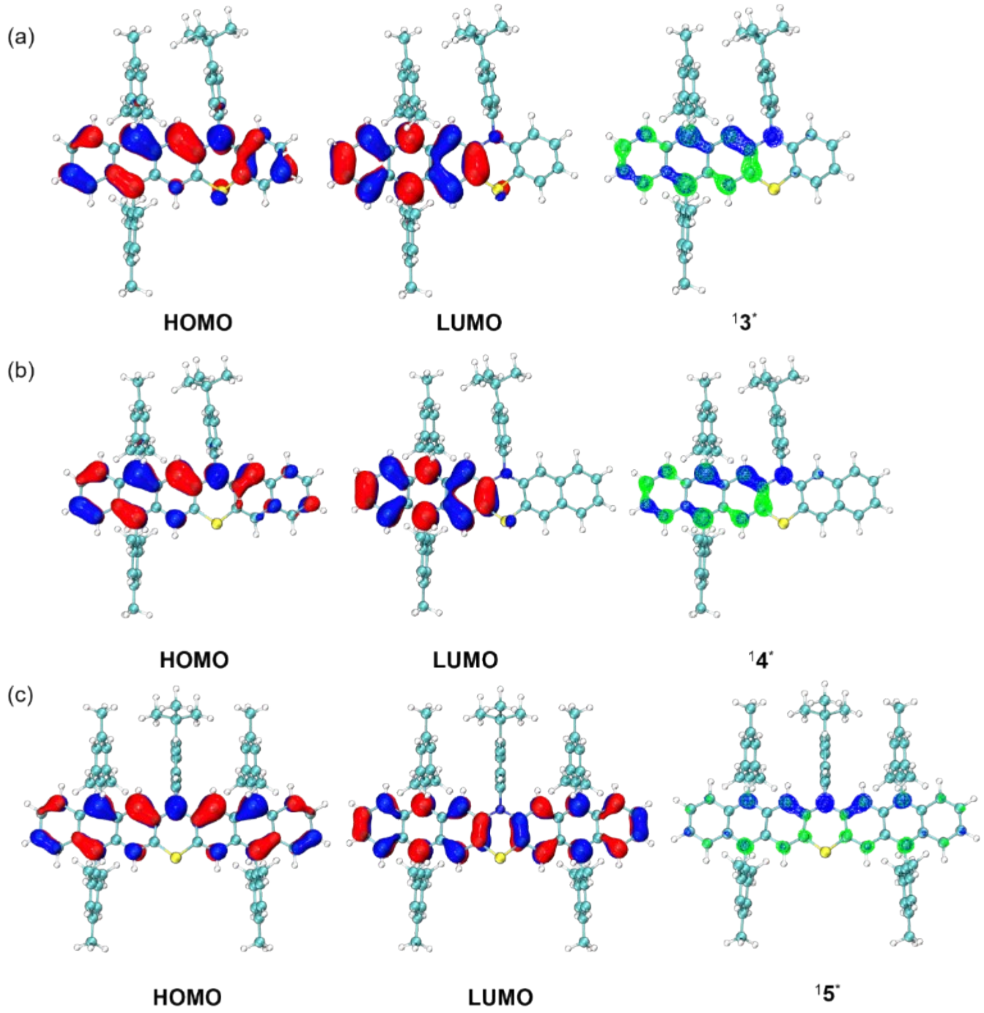

Figure S9. Calculated molecular orbitals of **3**, **4**, and **5** and their real space representations of hole and electron distributions for S0→S1 excitation. Blue and green regions denote the hole and electron distributions, respectively (isovalue = 0.003). S0→S1 was mainly attributed to electrons transition from HOMO→LUMO (isovalue = 0.03).

4 UV-vis-NIR titrations

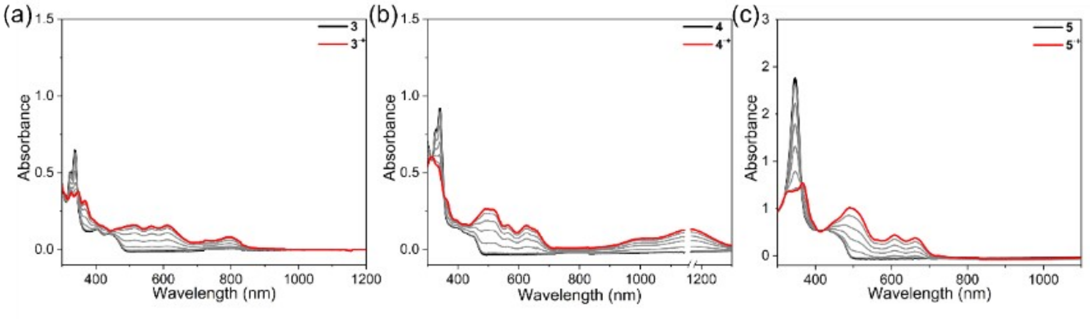

Figure S10. UV-vis-NIR titrations of **3** (a), **4** (b) and **5** (c) (15 μM in DCM) using $AgSbF_6$ as oxidant.

5 EPR studies

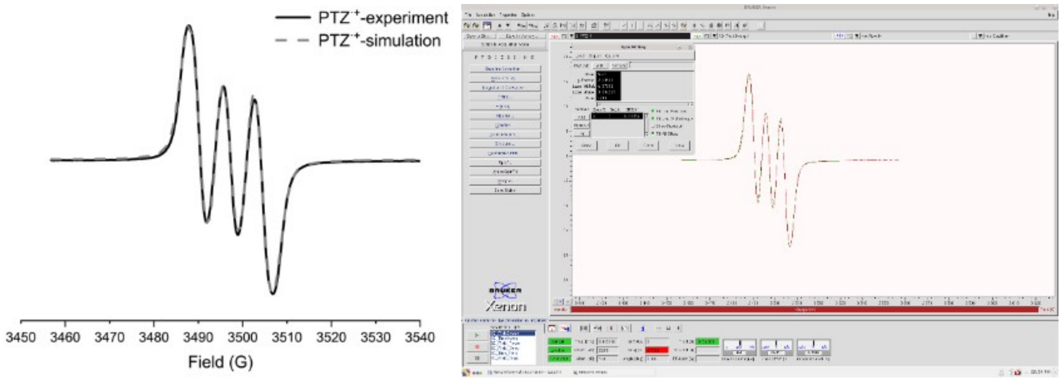

Figure S11. Experimental and simulated EPR spectra of $PTZ^{\bullet+}$ in CH_2Cl_2 (data processing (left) and screenshot (right) from the Bruker SpinFit software). The fitting parameters for the spectral simulation are: $g = 2.00529$, $A_N = 6.99874$ G.

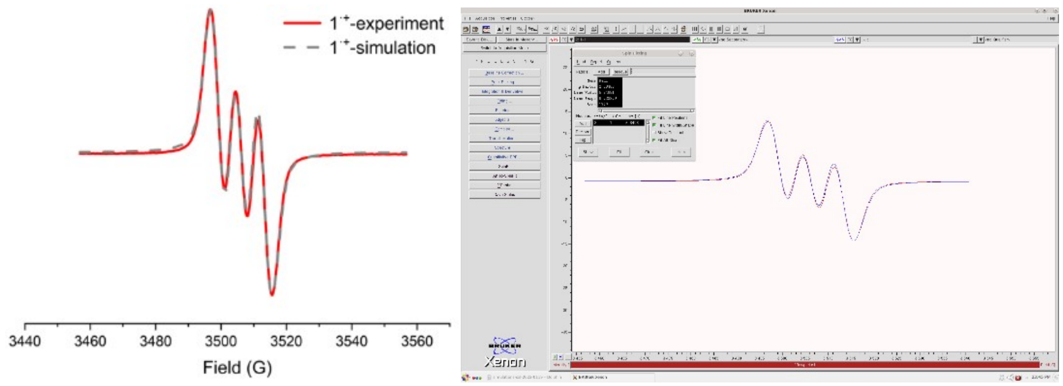

Figure S12. Experimental and simulated EPR spectra of **1**$^{\bullet+}$ in CH_2Cl_2 (data processing (left) and screenshot (right) from the Bruker SpinFit software). The fitting parameters for the spectral simulation are: g = 2.00463, A_N = 6.8498 G.

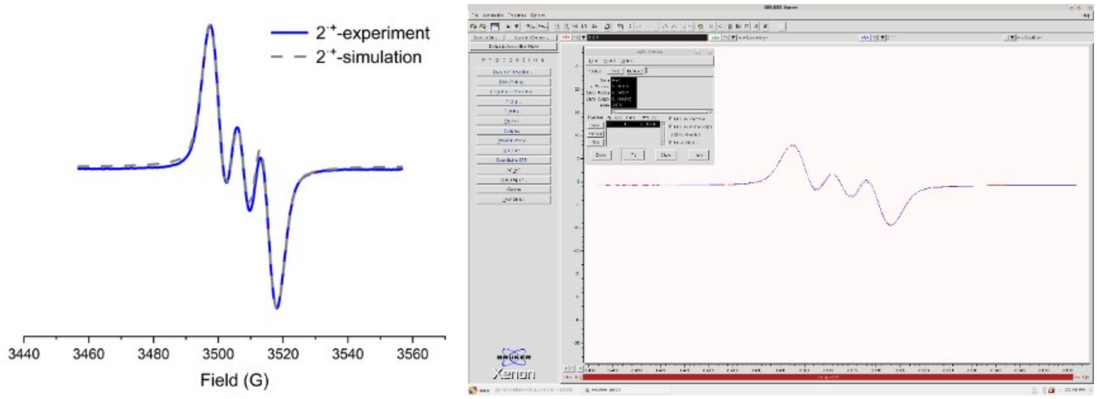

Figure S13. Experimental and simulated EPR spectra of **2**$^{\bullet+}$ in CH_2Cl_2 (data processing (left) and screenshot (right) from the Bruker SpinFit software). The fitting parameters for the spectral simulation are: g = 2.00407, A_N = 7.28938 G.

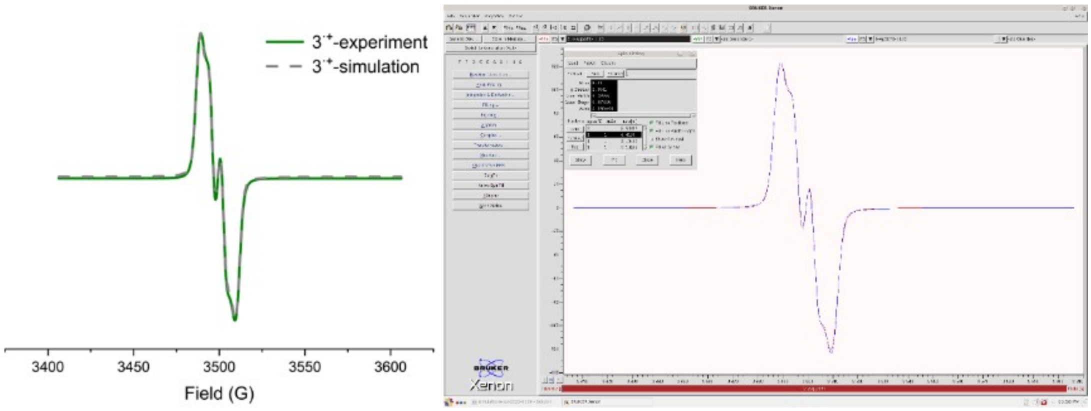

Figure S14. Experimental and simulated EPR spectra of **3**$^{\bullet+}$ in CH_2Cl_2 (data processing (left) and screenshot (right) from the Bruker SpinFit software). The fitting parameters for the spectral simulation are: g = 2.0041, A_N = 5.93397 G, A_{Ha} = 4.4158 G, A_{Hb} = 2.10443 G, A_{Hc} = 2.10402 G.

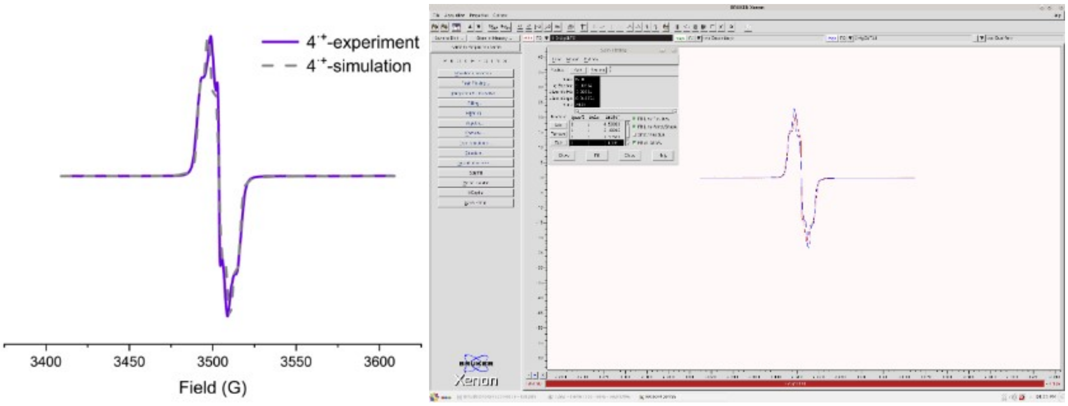

Figure S15. Experimental and simulated EPR spectra of $\mathbf{4}^{\bullet+}$ in CH_2Cl_2 (data processing (left) and screenshot (right) from the Bruker SpinFit software). The fitting parameters for the spectral simulation are: g = 2.0041, A_N = 4.50023 G, A_{Ha} = 5.49945 G, A_{Hb} = 3.61345 G, A_{Hc} = 2.17639 G, A_{Hd} = 1.0399 G.

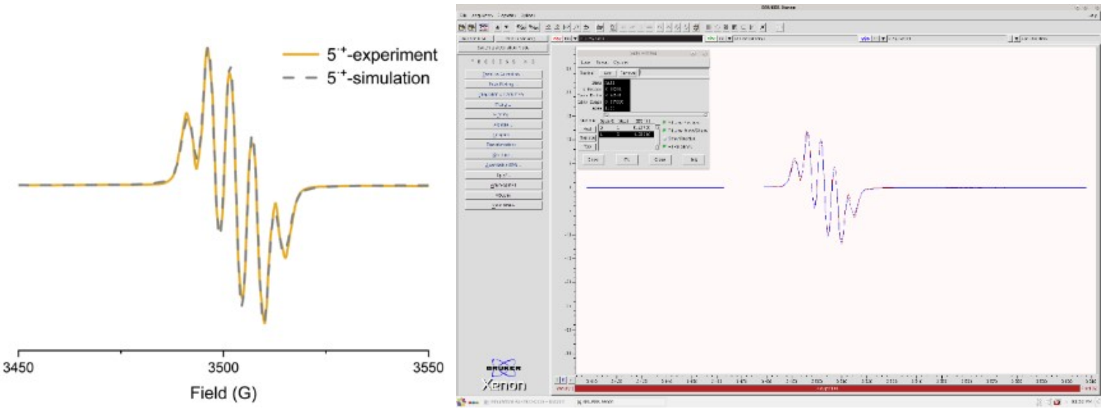

Figure S16. Experimental and simulated EPR spectra of $\mathbf{5}^{\bullet+}$ in CH_2Cl_2 (data processing (left) and screenshot (right) from the Bruker SpinFit software). The fitting parameters for the spectral simulation are: g = 2.00281, A_N = 5.47755 G, A_H= 4.59686 G.

Table S2. The g and A_N, A_H value analysis of $PTZ^{\bullet+}$, $\mathbf{1}^{\bullet+}$, $\mathbf{2}^{\bullet+}$, $\mathbf{3}^{\bullet+}$, $\mathbf{4}^{\bullet+}$ and $\mathbf{5}^{\bullet+}$.

	$PTZ^{\bullet+}$	$\mathbf{1}^{\bullet+}$	$\mathbf{2}^{\bullet+}$	$\mathbf{3}^{\bullet+}$	$\mathbf{4}^{\bullet+}$	$\mathbf{5}^{\bullet+}$
g	2.00529	2.00463	2.00407	2.0041	2.0041	2.00281
A_N (G)	6.99874	6.8498	7.28938	5.93397	4.50023	5.47755
A_H (G)	-	-	-	4.4158<br>2.10443<br>2.10402	5.49945<br>2.17639<br>1.0399<br>3.61345	4.59686

6 Single crystal data

6.1 Single crystals growth of 1-5

(1) Neutral crystals

Crystals of compounds **1-5** were grown via a vapor diffusion method: compounds **1-5** (5.0 μmol) were dissolved in dichloromethane (1.0 mL) solution and placed it in a 4.0 mL bottle. The small bottle was then placed in a 20 mL glass bottle containing *n*-hexane (5.0 mL) for several days, after that the transparently white or yellow crystals **1-5** were obtained.

(2) Radical crystals

The radical crystals of compounds **1**, **2** was grown via a vapor diffusion method: Compound **1** or **2** (5.0 μmol) was dissolved in dichloromethane, and oxidized by $AgSbF_6$ (5.0 μmol) and placed it in a 4.0 mL bottle. The liquid turns from light yellow to green. The small bottle was then placed in a 20 mL glass bottle containing *n*-hexane (5 mL) for several days, after that the transparently purple or green crystal $\mathbf{1}^{\bullet+}$ or $\mathbf{2}^{\bullet+}$ was obtained.

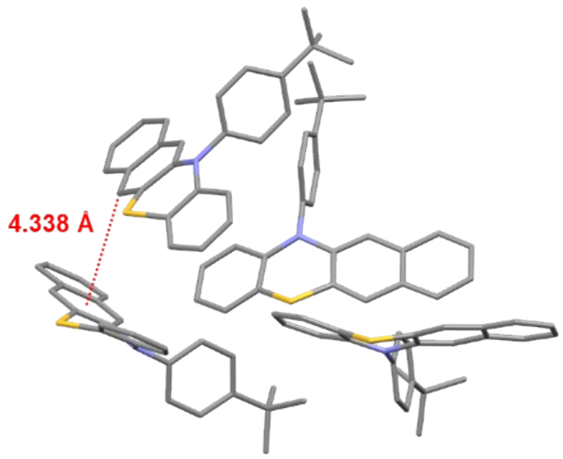

Figure S17. Packing structure of **1** at 201 K in the crystalline state. Other hydrogen atoms are omitted for clarity.

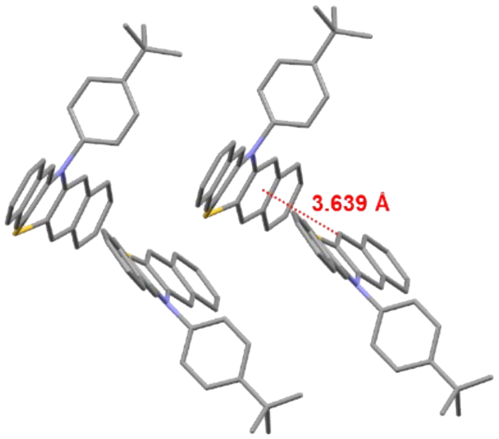

Figure S18. Packing structure of **2** at 172.99 K in the crystalline state. Other hydrogen atoms are omitted for clarity.

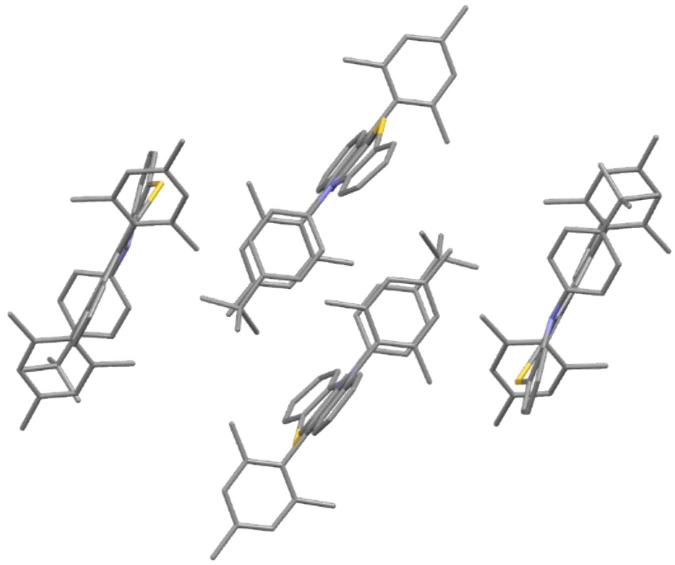

Figure S19. Packing structure of **3** at 170 K in the crystalline state. Other hydrogen atoms are omitted for clarity.

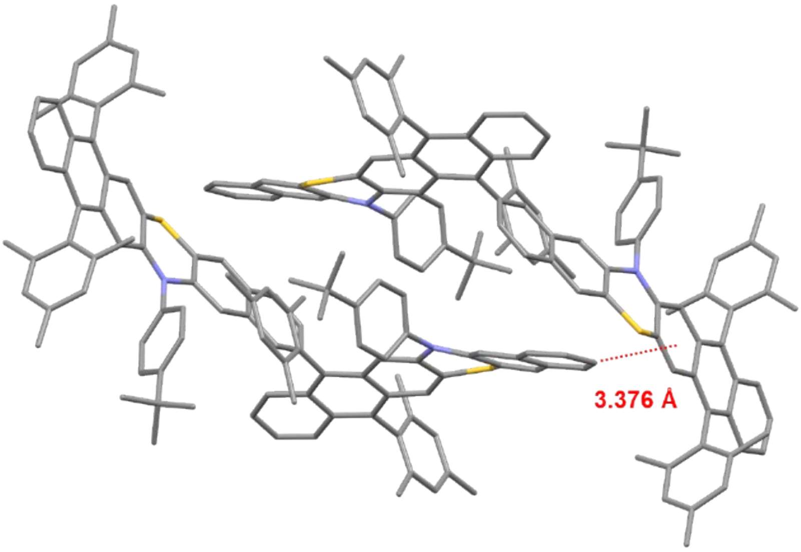

Figure S20. Packing structure of **4** at 170 K in the crystalline state. Other hydrogen atoms are omitted for clarity.

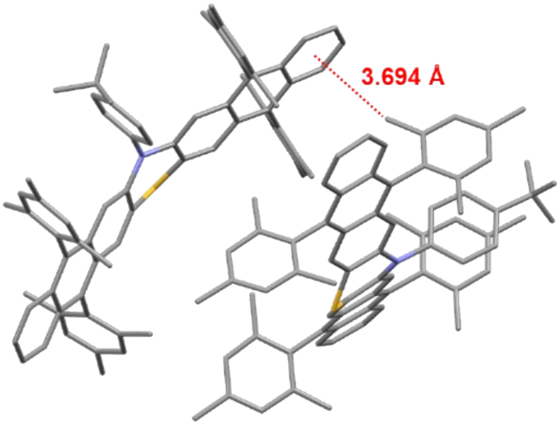

Figure S21. Packing structure of **5** at 173 K in the crystalline state. Other hydrogen atoms are omitted for clarity.

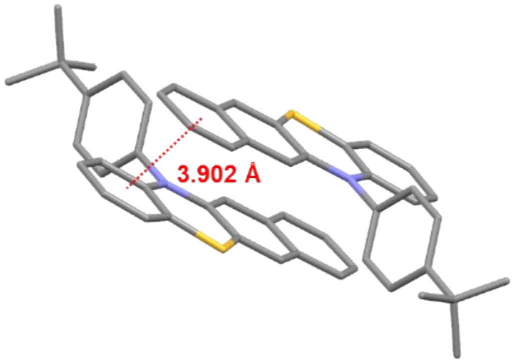

Figure S22. Packing structure of $\mathbf{1}^{\bullet+}$ at 296.15 K in the crystalline state. Other hydrogen atoms are omitted for clarity.

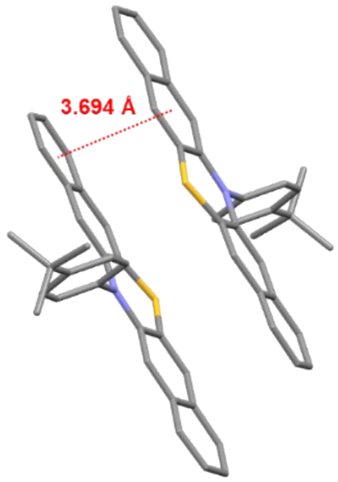

Figure S23. Packing structure of $\mathbf{2}^{\bullet+}$ at 169.99 K in the crystalline state. Other hydrogen atoms are omitted for clarity.

6.2 Single crystals data

Table S3. X-ray single crystal data of metallacycles **1** and $\mathbf{1}^{\bullet+}$.

**Compound**	**1**	$\mathbf{1}^{\bullet+}$
Empirical formula	$C_{26}H_{23}NS$	$C_{13}H_{11.5}F_3N_{0.5}S_{0.5}Sb_{0.5}$
Formula weight	381.51	308.63
Temperature/K	201(40)	296.15
Crystal system	orthorhombic	monoclinic
Space group	Pccn	C2/m
a / Å	23.8497(9)	27.184(5)
b / Å	21.8615(7)	6.8384(13)
c / Å	7.9142(3)	19.415(4)
α / °	90	90
β / °	90	133.036(3)
γ / °	90	90

Volume / Å^3	4126.4(3)	2638.1(9)
Z	8	8
ρ_{calc}g / cm^3	1.228	1.554
μ / mm^{-1}	1.453	1.182
F (000)	1616.0	1228.0
Crystal size / mm^3	0.36 × 0.14 × 0.12	0.06 × 0.06 × 0.05
Radiation	CuKα (λ = 1.54184)	MoKα (λ = 0.71073)
2θ range for data collection/ °	7.414 to 134.16	6.712 to 55.276
Index ranges	-28 ≤ h ≤ 28, -26 ≤ k ≤ 25, -9 ≤ l ≤ 6	-35≤ h ≤ 35, -8 ≤ k ≤ 8, -25 ≤ l ≤ 25
Reflections collected	16520	10582
Independent reflections	3670 R_{int} = 0.0673, R_{sigma} = 0.0620	3204 R_{int} = 0.0331, R_{sigma} = 0.0342
Data / restraints / parameters	3670/14/256	3204/52/222
Goodness-of-fit on F^2	1.068	1.015
Final R indexes [I >= 2σ (I)]	R_1 = 0.0834, wR_2 = 0.2097	R_1 = 0.0590, wR_2 = 0.1625
Final R indexes [all data]	R_1 = 0.0987, wR_2 = 0.2207	R_1 = 0.0864, wR_2 = 0.1914
Largest diff. peak / hole / e Å$^-$	1.77/-0.47	1.58/-0.63
CCDC	2145133	2145124

Table S4. X-ray single crystal data of metallacycles **2** and **2**$^{•+}$.

**Compound**	**2**	**2**$^{•+}$
Empirical formula	$C_{31}H_{27}Cl_2NS$	$C_{30}H_{25}F_6NSSb$
Formula weight	516.49	667.32
Temperature/K	172.99(10)	169.99(10)
Crystal system	monoclinic	monoclinic
Space group	P2$_1$/n	C2/c
a / Å	11.18030(10)	15.8665(18)
b / Å	7.73940(10)	24.950(2)
c / Å	30.7513(3)	16.2233(13)
α / °	90	90
β / °	99.9610(10)	99.104(9)
γ / °	90	90
Volume / Å^3	2620.76(5)	6341.4(10)
Z	4	8
ρ_{calc}g / cm^3	1.309	1.398
μ / mm^{-1}	3.118	7.991
F (000)	1080.0	2664.0
Crystal size / mm^3	0.36 × 0.34 × 0.28	0.28 × 0.13 × 0.08
Radiation	CuKα (λ = 1.54184)	CuKα (λ = 1.54184)
2θ range for data collection / °	8.054 to 134.15	6.662 to 134.156
Index ranges	-13 ≤ h ≤ 13, -9 ≤ k ≤ 9, -36 ≤ l ≤ 36	-18 ≤ h ≤ 18, -29 ≤ k ≤ 29, -19 ≤ l ≤ 13
Reflections collected	56983	30155
Independent reflections	4679 R_{int} = 0.0599, R_{sigma} = 0.0260	5659 R_{int} = 0.1462, R_{sigma} = 0.1091
Data / restraints / parameters	4679/48/349	5659/0/355
Goodness-of-fit on F^2	1.032	1.017
Final R indexes [I >= 2σ (I)]	R_1 = 0.0534, wR_2 = 0.1357	R_1 = 0.0653, wR_2 = 0.1592

Final R indexes [all data]	$R_1 = 0.0557$, $wR_2 = 0.1377$	$R_1 = 0.1228$, $wR_2 = 0.1864$
Largest diff. peak / hole / e Å^{-3}	0.49/-0.65	0.68/-1.72
CCDC	2143328	2143327

Table S5 X-ray single crystal data of **3** and **4**.

**Compound**	**3**	**4**
Empirical formula	$C_{48}H_{45}NS$	$C_{52}H_{47}NS$
Formula weight	667.91	717.96
Temperature/K	170.00(10)	170.00(10)
Crystal system	monoclinic	trigonal
Space group	$P2_1/n$	R-3
a / Å	16.8923(2)	36.5917(5)
b / Å	13.29240(10)	36.5917(5)
c / Å	17.9856(3)	17.7385(3)
α / °	90	90
β / °	113.172(2)	90
γ / °	90	120
Volume / Å^3	3712.68(10)	20569.0(7)
Z	4	18
ρ_{calc}g / cm^3	1.195	1.043
μ / mm^{-1}	1.022	0.862
F (000)	1424.0	6876.0
Crystal size / mm^3	0.26 × 0.22 × 0.18	0.32 × 0.24 × 0.12
Radiation	CuKα (λ = 1.54184)	CuKα (λ = 1.54184)
2θ range for data collection / °	6.084 to 134.142	5.71 to 134.152
Index ranges	$-20 \le h \le 12$, $-15 \le k \le 15$, $-21 \le l \le 21$	$-43 \le h \le 26$, $-43 \le k \le 29$, $-21 \le l \le 21$
Reflections collected	40600	67250
Independent reflections	6597 $R_{int} = 0.0396$, $R_{sigma} = 0.0275$	8171 $R_{int} = 0.0367$, $R_{sigma} = 0.0188$
Data / restraints / parameters	6597/0/460	8171/0/496
Goodness-of-fit on F^2	1.030	1.057
Final R indexes [I >= 2σ (I)]	$R_1 = 0.0410$, $wR_2 = 0.1094$	$R_1 = 0.0404$, $wR_2 = 0.1079$
Final R indexes [all data]	$R_1 = 0.0460$, $wR_2 = 0.1128$	$R_1 = 0.0443$, $wR_2 = 0.1099$
Largest diff. peak / hole / e Å^{-3}	0.51/-0.36	0.45/-0.29
CCDC	2143332	2143331

Table S6 X-ray single crystal data of **5** and **a-OMe**.

**Compound**	**5**	**a-OMe**
Empirical formula	$C_{74}H_{69}NS$	$C_{18}H_{16}O_2S$
Formula weight	1004.36	296.37
Temperature/K	173.00(10)	173.00(10)
Crystal system	triclinic	orthorhombic
Space group	P-1	Pbcn
a / Å	7.16900(10)	19.5758(2)
b / Å	17.2492(3)	8.45120(10)
c / Å	25.1684(5)	18.7056(2)
α / °	106.660(2)	90
β / °	90.552(2)	90
γ / °	96.116(2)	90

Volume / Å^3	2962.06(9)	3094.64(6)
Z	2	8
ρ_{calc}g / cm^3	1.126	1.272
μ / mm^{-1}	0.799	1.863
F (000)	1072.0	1248.0
Crystal size / mm^3	0.36 × 0.24 × 0.22	0.32 × 0.26 × 0.22
Radiation	CuKα (λ = 1.54184)	CuKα (λ = 1.54184)
2θ range for data collection / °	7.34 to 134.154	9.034 to 134.13
Index ranges	-8 ≤ h ≤ 8, -20 ≤ k ≤ 20, -30 ≤ l ≤ 30	-23 ≤ h ≤ 23, -10 ≤ k ≤ 10, -22 ≤ l ≤ 22
Reflections collected	74173	69070
Independent reflections	10555 R_{int} = 0.0607, R_{sigma} = 0.0388	2759 R_{int} = 0.0679, R_{sigma} = 0.0236
Data / restraints / parameters	10555/0/700	2759/0/192
Goodness-of-fit on F^2	1.010	1.087
Final R indexes [I >= 2σ (I)]	R_1 = 0.0547, wR_2 = 0.1406	R_1 = 0.0382, wR_2 = 0.0898
Final R indexes [all data]	R_1 = 0.0610, wR_2 = 0.1453	R_1 = 0.0416, wR_2 = 0.0919
Largest diff. peak / hole / e Å^{-3}	1.25/-0.89	0.17/-0.19
CCDC	2143509	2143510

Table S7 X-ray single crystal data of **a-OTf** and **3-methoxynaphthalene-2-thiol**.

**Compound**	**a-OTf**	**3-methoxynaphthalene-2-thiol**
Empirical formula	$C_{18}H_{10}F_6O_6S_3$	$C_{11}H_{10}OS$
Formula weight	532.44	190.25
Temperature/K	230(80)	229(80)
Crystal system	triclinic	triclinic
Space group	P-1	P1
a / Å	8.4229(2)	7.0221(4)
b / Å	10.6178(2)	7.9102(7)
c / Å	12.2594(2)	9.5017(5)
α / °	100.265(2)	106.659(7)
β / °	102.985(2)	105.234(5)
γ / °	94.205(2)	101.170(6)
Volume / Å^3	1043.88(4)	466.74(6)
Z	2	2
ρ_{calc}g / cm^3	1.694	1.354
μ / mm^{-1}	4.090	2.686
F (000)	536.0	200.0
Crystal size / mm^3	0.32 × 0.24 × 0.18	0.26 × 0.24 × 0.12
Radiation	CuKα (λ = 1.54184)	CuKα (λ = 1.54184)
2θ range for data collection/ °	7.554 to 134.15	10.328 to 134.16
Index ranges	-10 ≤ h ≤ 10, -12 ≤ k ≤ 12, -14 ≤ l ≤ 14	-8≤ h ≤ 8, -9 ≤ k ≤ 9, -11 ≤ l ≤ 11
Reflections collected	22837	6866
Independent reflections	3692 R_{int} = 0.0448, R_{sigma} = 0.0244	2954 R_{int} = 0.0618, R_{sigma} = 0.0652
Data / restraints / parameters	3692/0/298	2954/3/239
Goodness-of-fit on F^2	1.046	1.054

Final R indexes [I >= 2σ (I)]	R_1 = 0.0331, wR_2 = 0.0888	R_1 = 0.0822, wR_2 = 0.2480
Final R indexes [all data]	R_1 = 0.0355, wR_2 = 0.0905	R_1 = 0.0854, wR_2 = 0.2523
Largest diff. peak / hole / e Å$^-$	0.39/-0.27	1.04/-0.48
CCDC	2150682	2150683

7 Photostability study

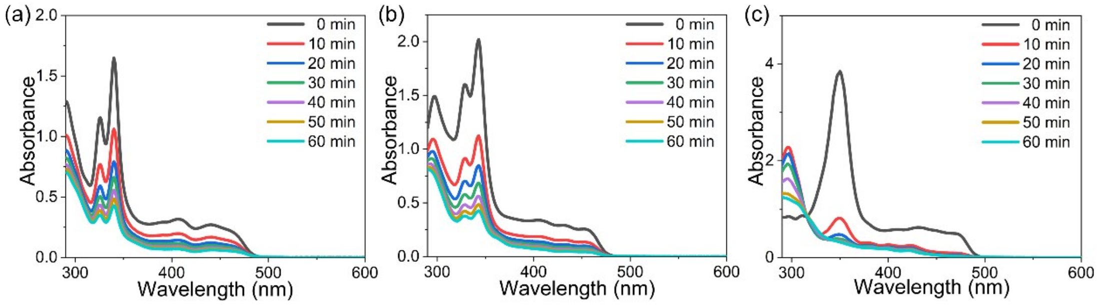

Figure S24. Changes of absorbance of **3** (a), **4** (b) and **5** (c) (30 μM in toluene) in absorption spectra upon irradiation by 6 W white LED light every ten minutes. Details of photostability test: A solution of extended phenothiazine (30 μM in toluene) in quartz cell was subjected to 6 W white LED irradiation in the photoreactor, during which the UV-Vis spectra of the solution were measured every ten minutes over a period of one hour to record the absorbance changes. The photostability experiments were repeated 3 times under the same testing condition.

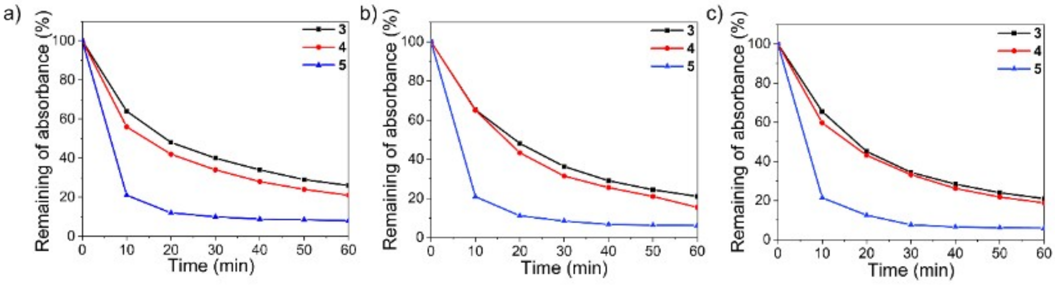

Figure S25. The photostability data of **3–5** from three repeated photostability experiments.

8 Comparison of catalytic performance of different catalysts

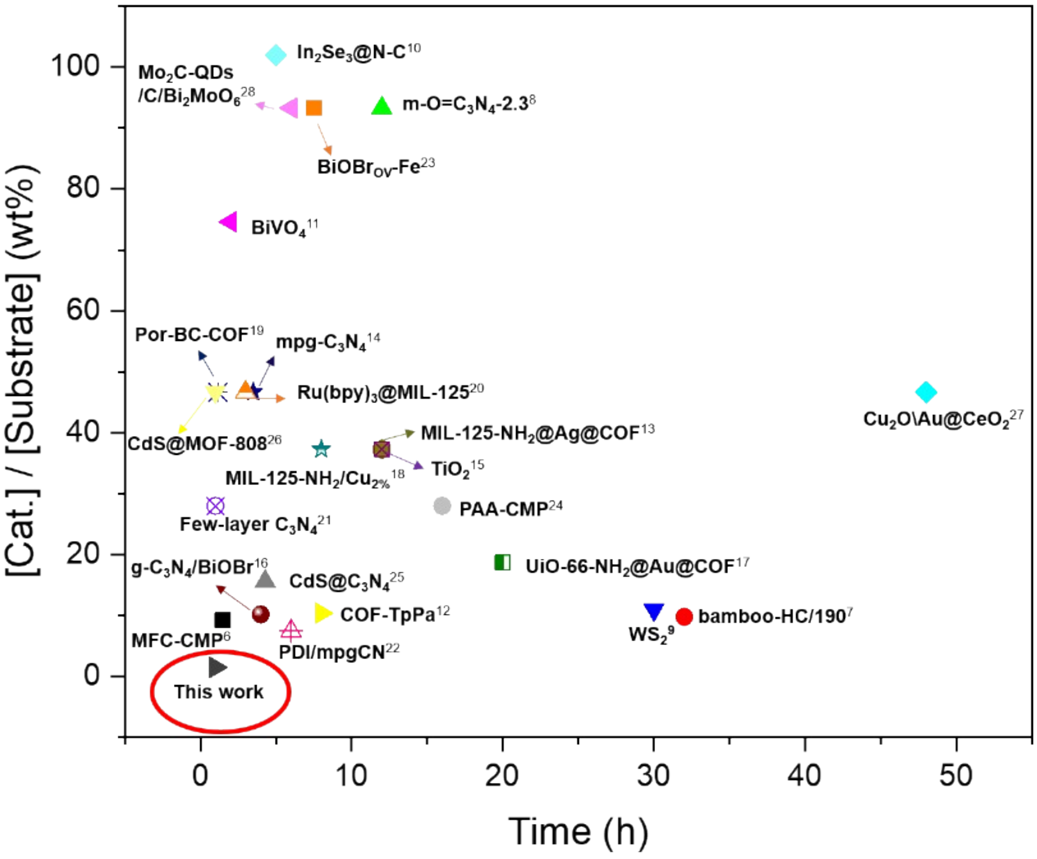

Figure S26. Comparison of catalytic performance of different catalysts for oxidative coupling of amines to imines.[5-28]

9 Sunlight-driven oxidative coupling of amines to imines

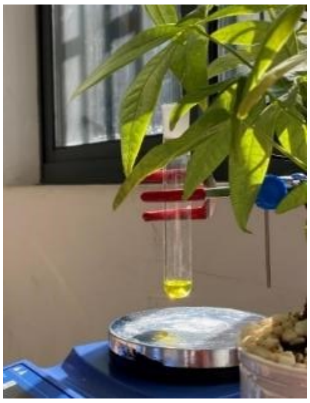

Figure S27. Efficient conversion of amines to imines under sunlight in an air atmosphere.

10 NMR spectra

10.1 ^{1}H and ^{13}C NMR spectra of new compounds

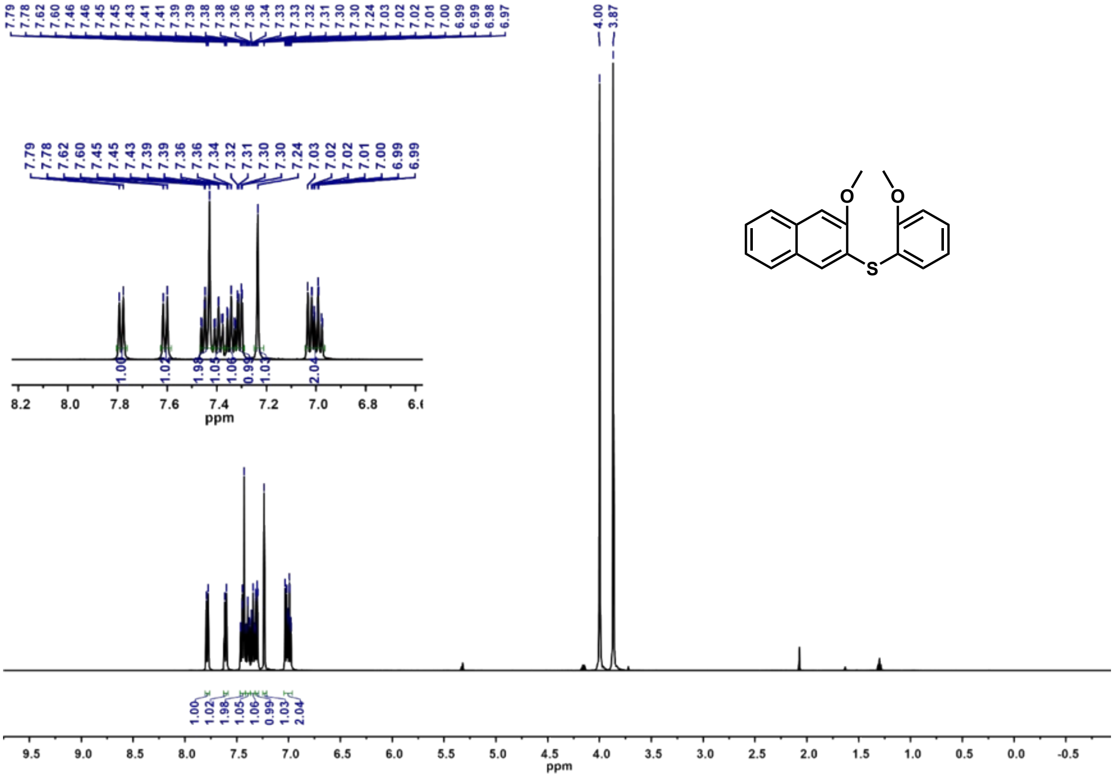

Figure S28. The ^{1}H NMR (500 MHz, 298 K) spectrum of compound **a-OMe** in CD_2Cl_2.

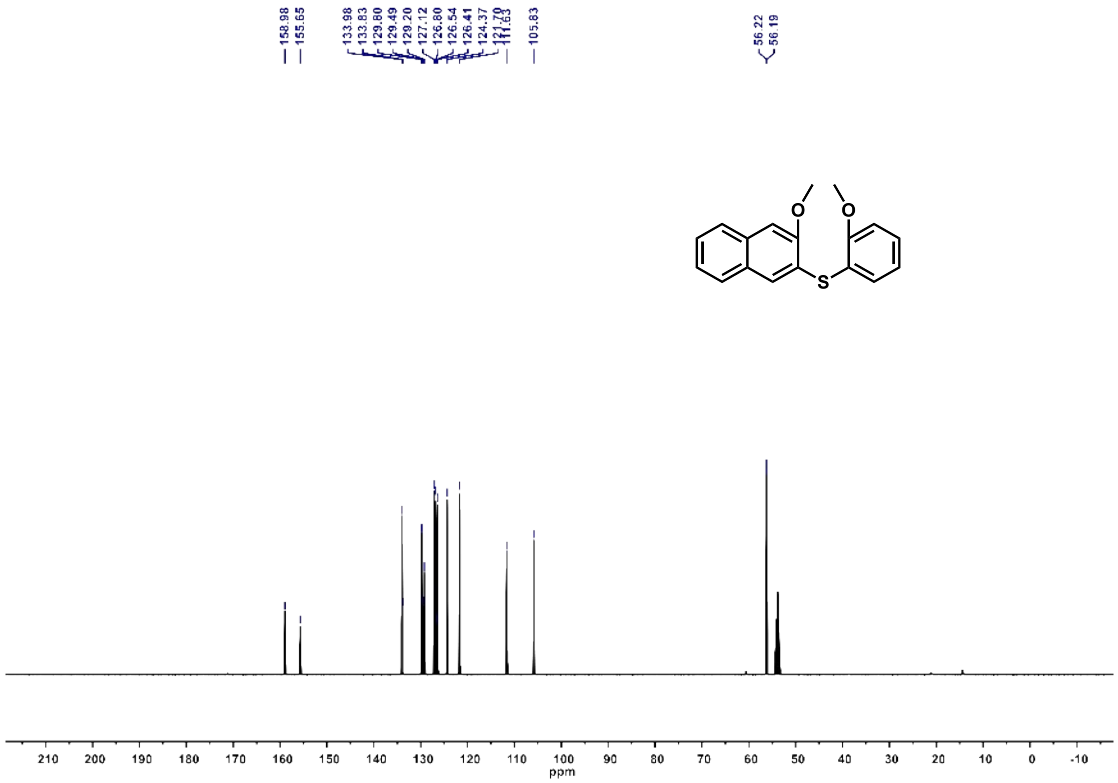

Figure S29. The ^{13}C NMR (125 MHz, 298 K) spectrum of compound **a-OMe** in CD_2Cl_2.

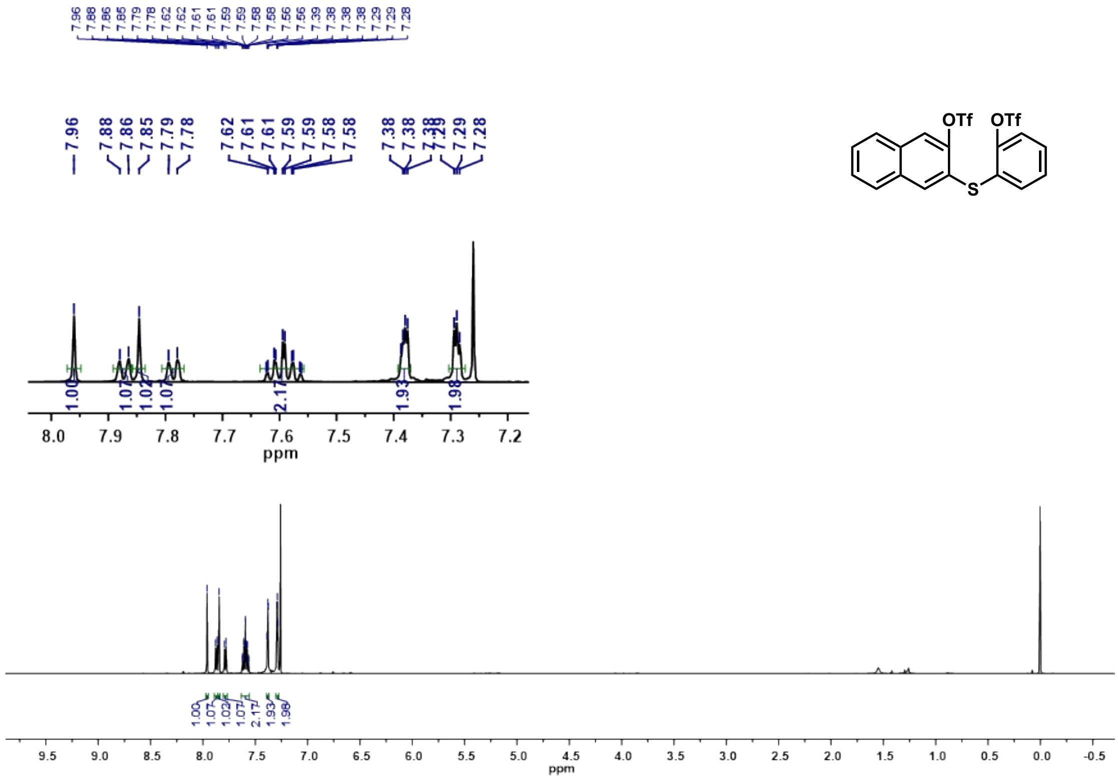

Figure S30. The ^{1}H NMR (500 MHz, 298 K) spectrum of compound **a-OTf** in $CDCl_3$.

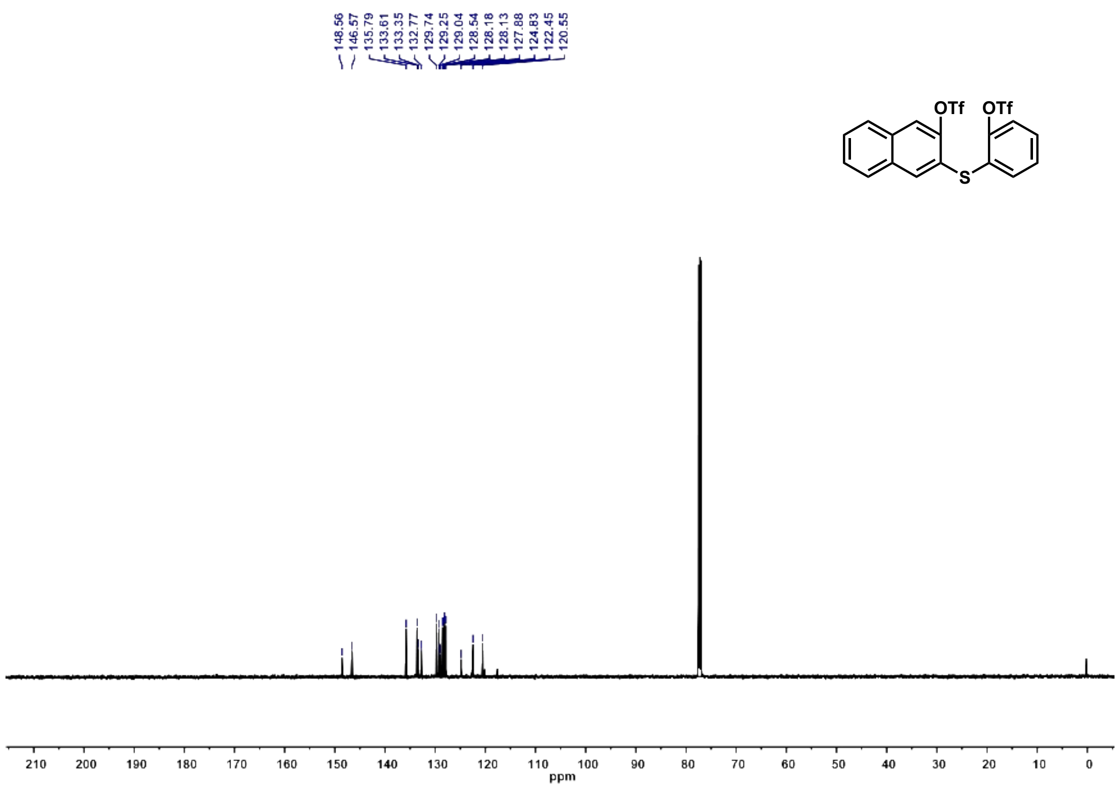

Figure S31. The ^{13}C NMR (125 MHz, 298 K) spectrum of compound **a-OTf** in $CDCl_3$.

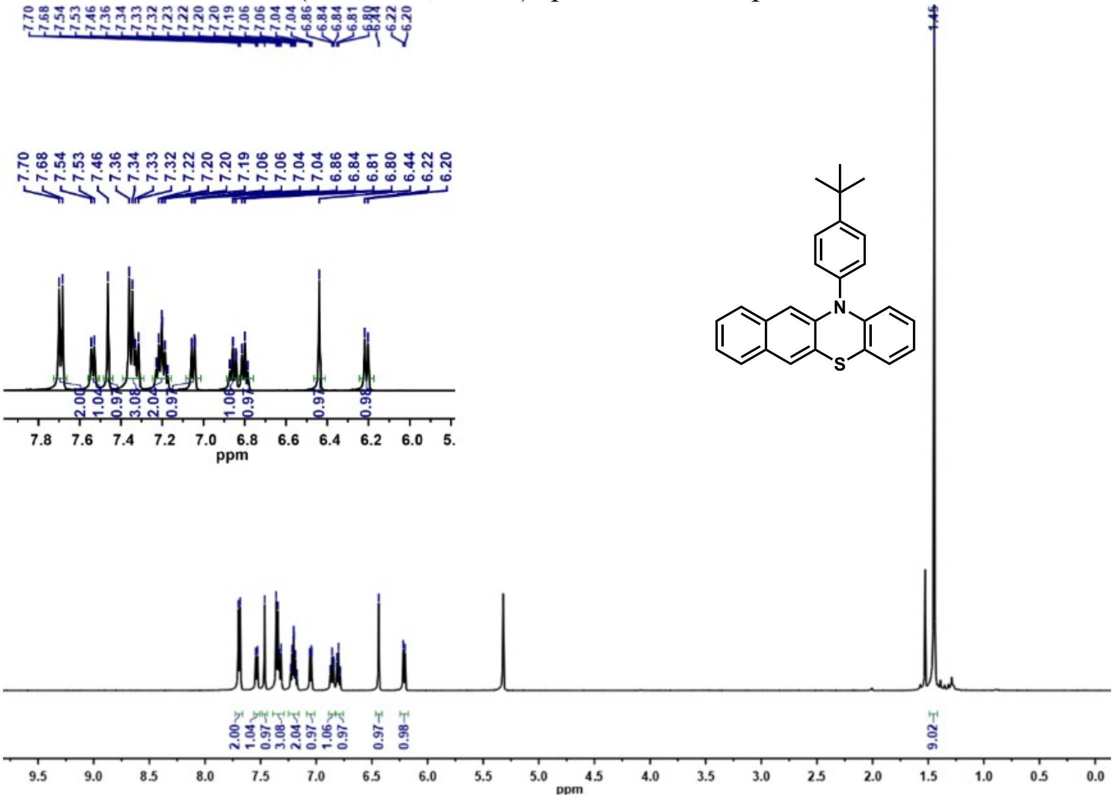

Figure S32. The ^{1}H NMR (500 MHz, 298 K) spectrum of compound **1** in CD_2Cl_2.

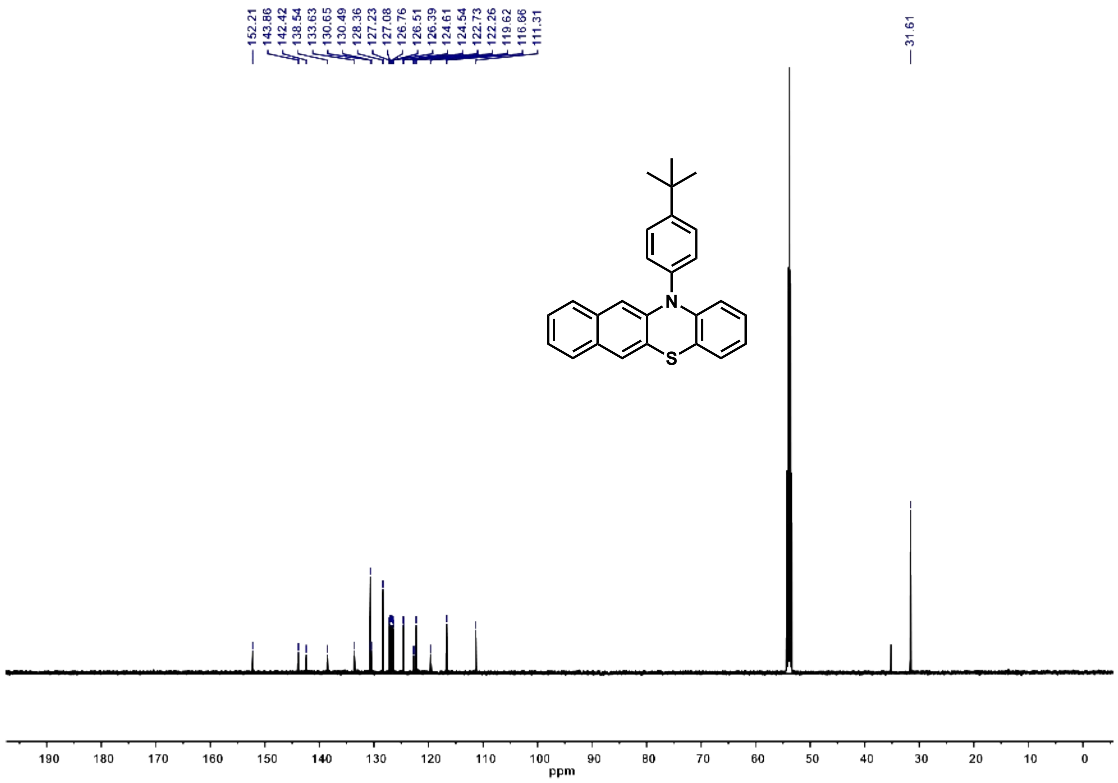

Figure S33. The ^{13}C NMR (125 MHz, 298 K) spectrum of compound **1** in CD_2Cl_2.

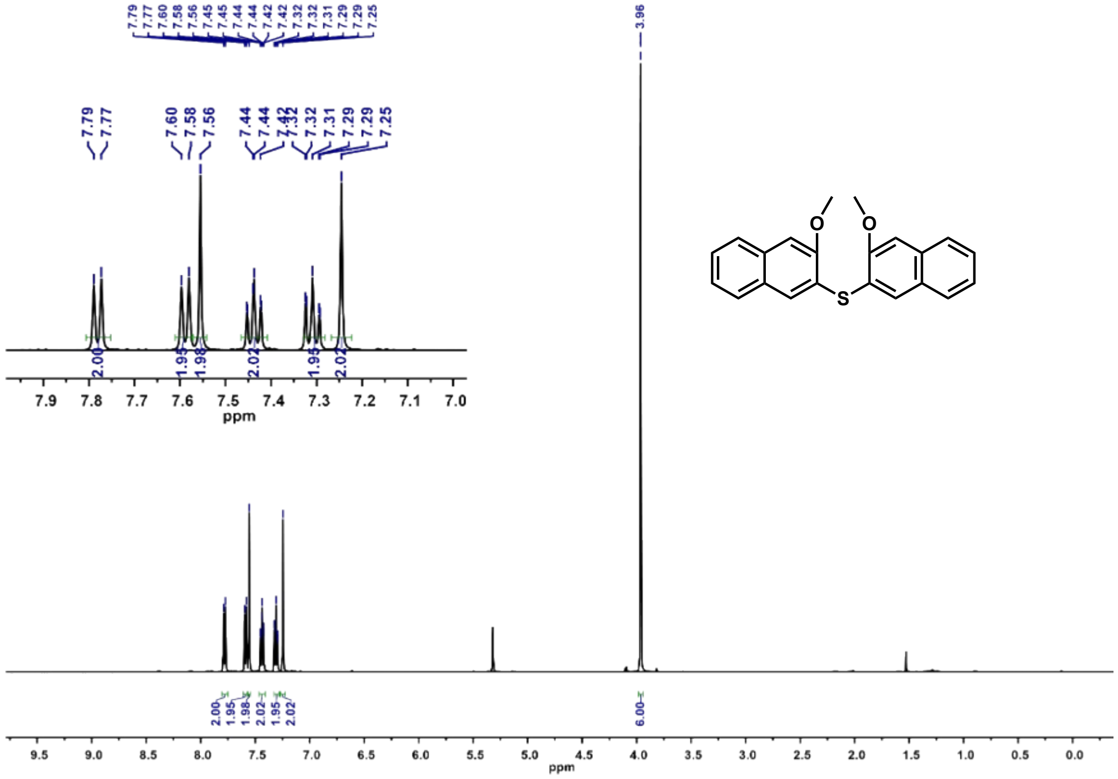

Figure S34. The ^{1}H NMR (500 MHz, 298 K) spectrum of compound **b-OMe** in CD_2Cl_2.

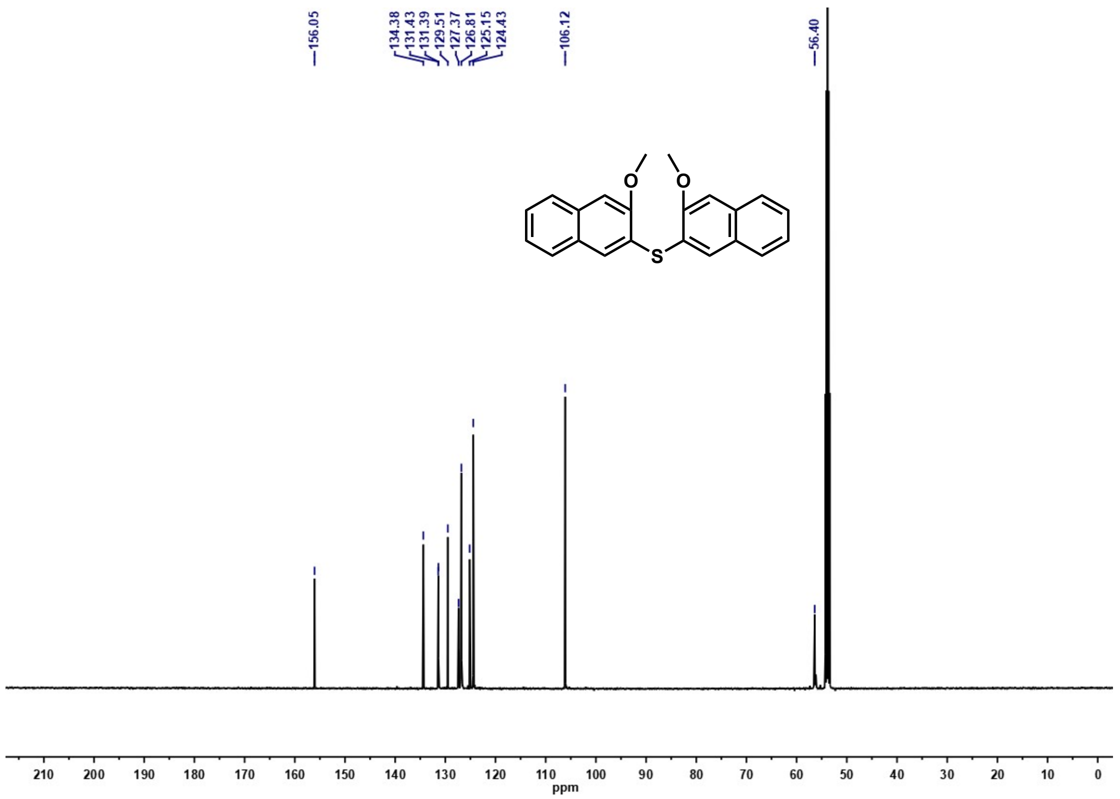

Figure S35. The ^{13}C NMR (125 MHz, 298 K) spectrum of compound **b-OMe** in CD_2Cl_2.

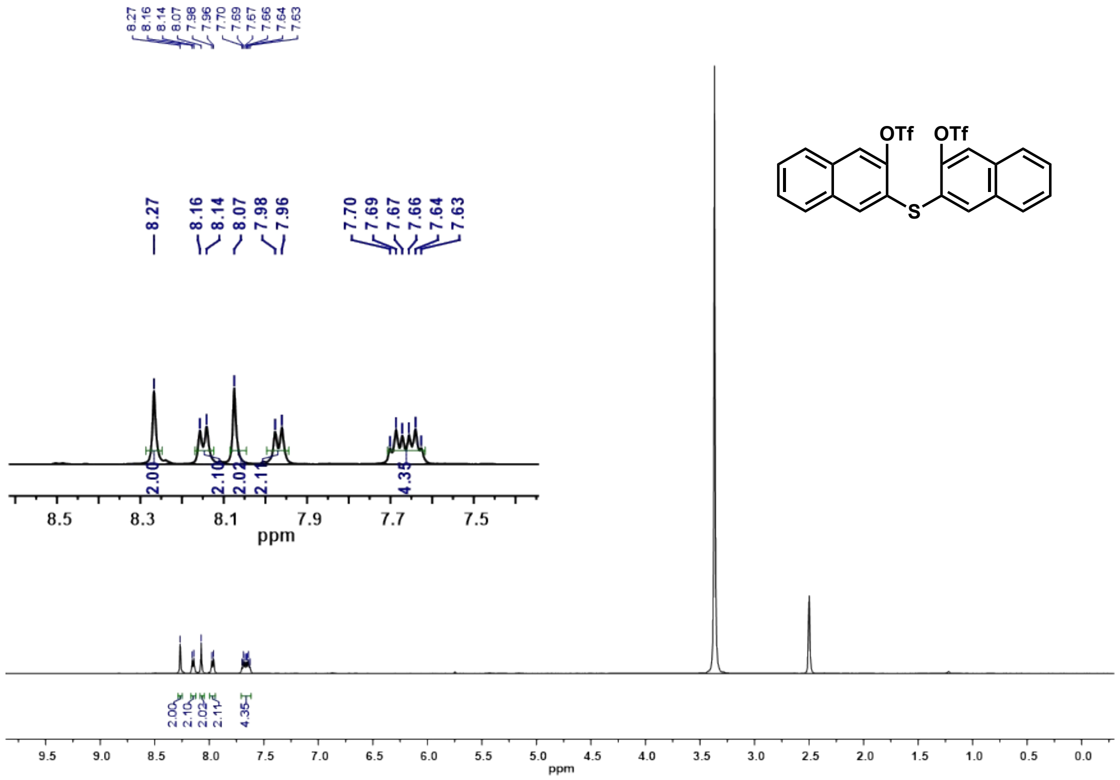

Figure S36. The ^{1}H NMR (500 MHz, 298 K) spectrum of compound **b-OTf** in DMSO-d_6.

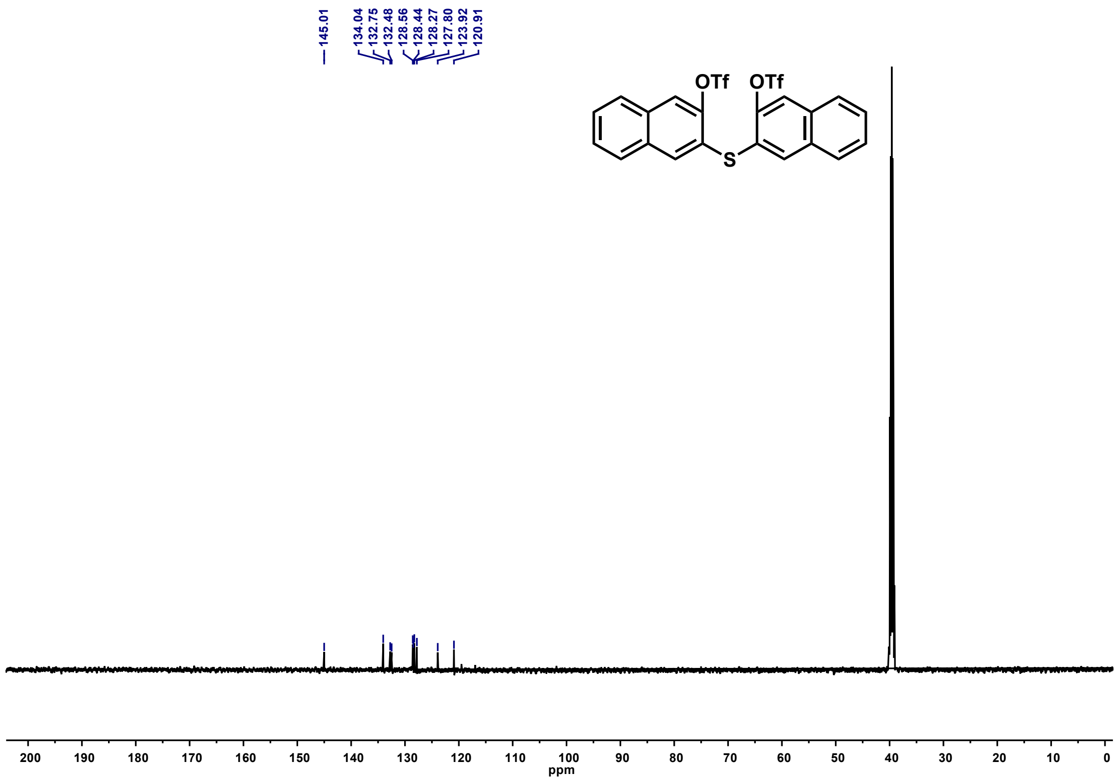

Figure S37. The ^{13}C NMR (125 MHz, 298 K) spectrum of compound **b-OTf** in DMSO-d_6.

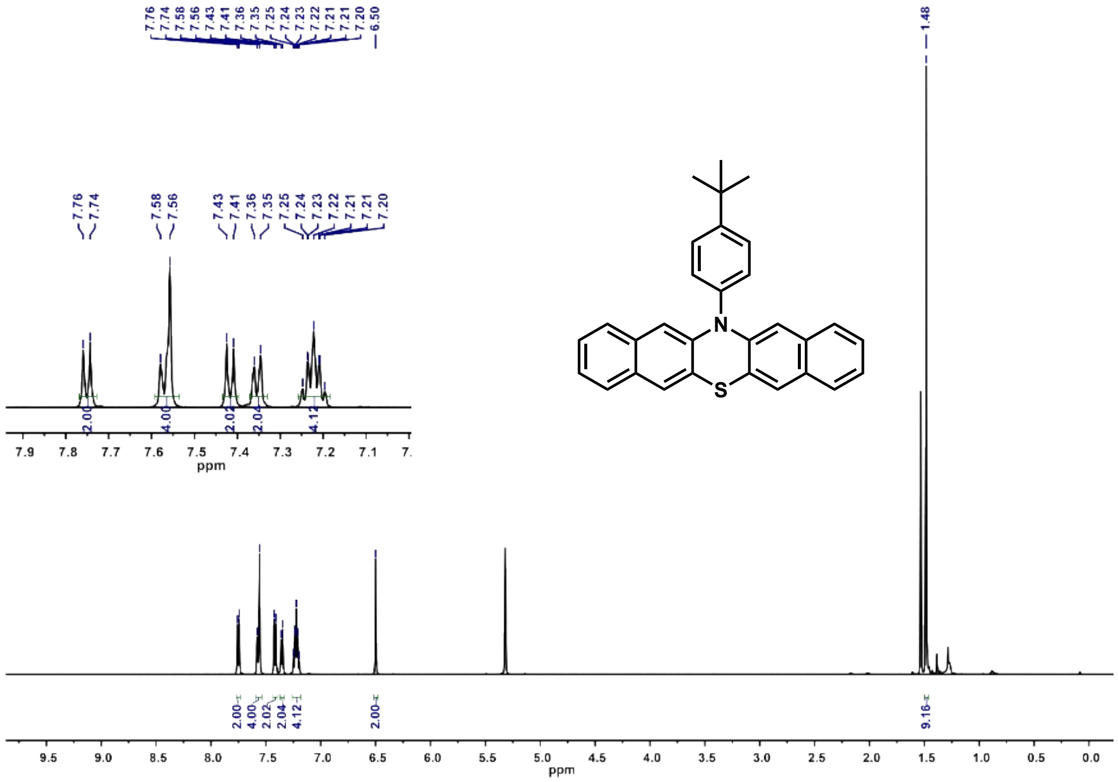

Figure S38. The ^{1}H NMR (500 MHz, 298 K) spectrum of compound **2** in CD_2Cl_2.

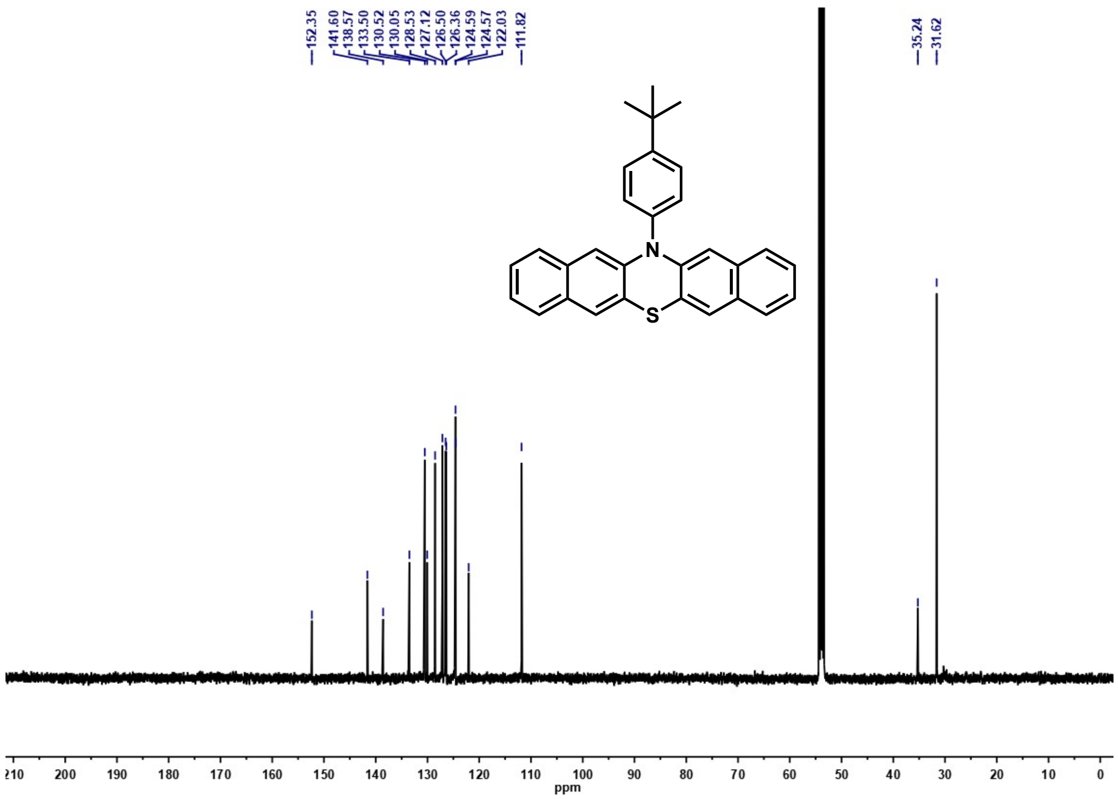

Figure S39. The ^{13}C NMR (125 MHz, 298 K) spectrum of compound **2** in CD_2Cl_2.

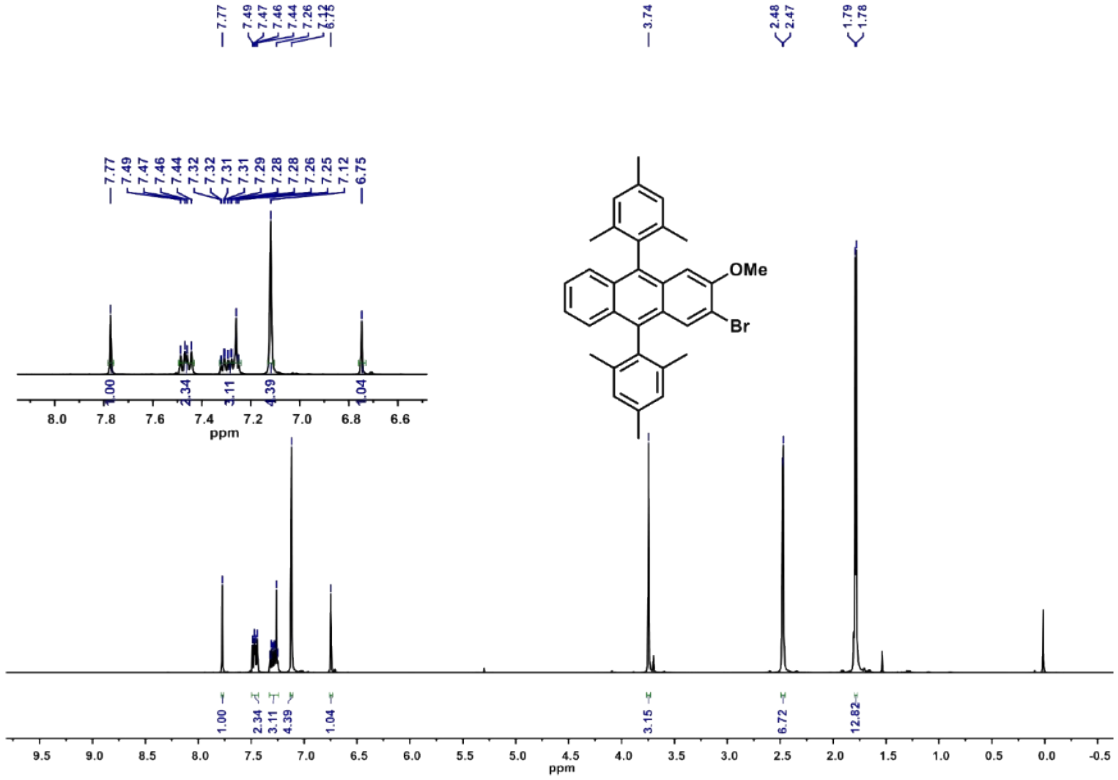

Figure S40. The ^{1}H NMR (500 MHz, 298 K) spectrum of compound **2-Br** in $CDCl_3$.

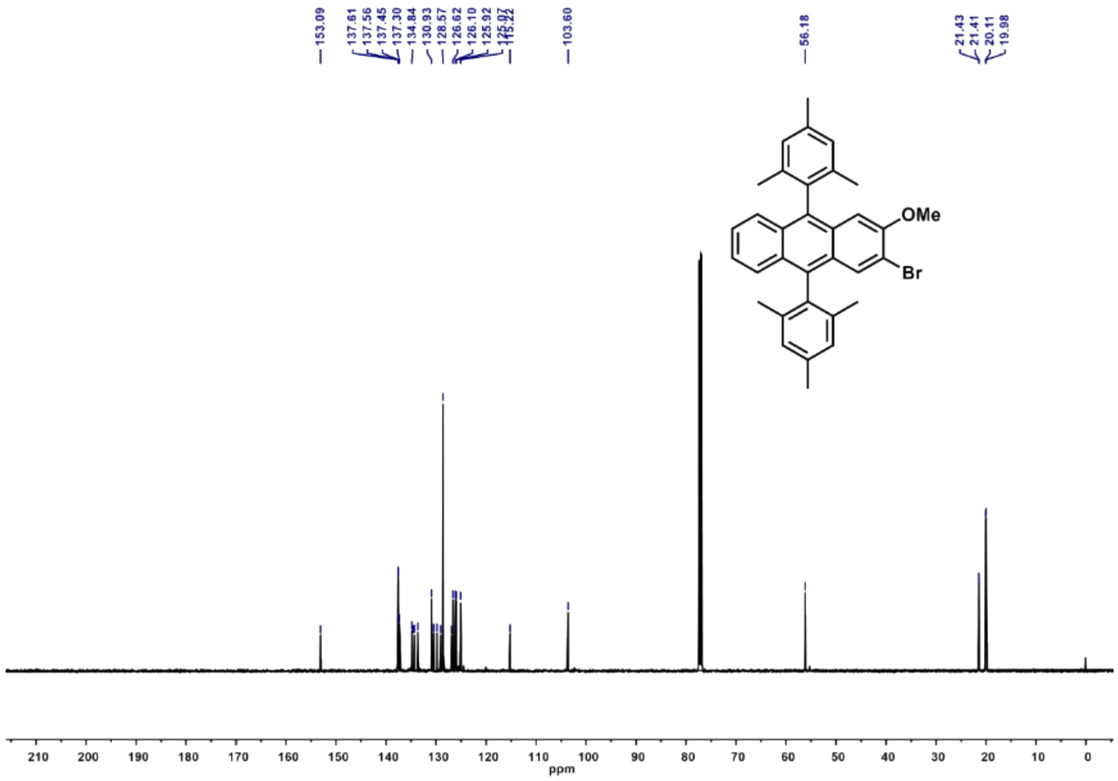

Figure S41. The ^{13}C NMR (125 MHz, 298 K) spectrum of compound **2-Br** in $CDCl_3$.

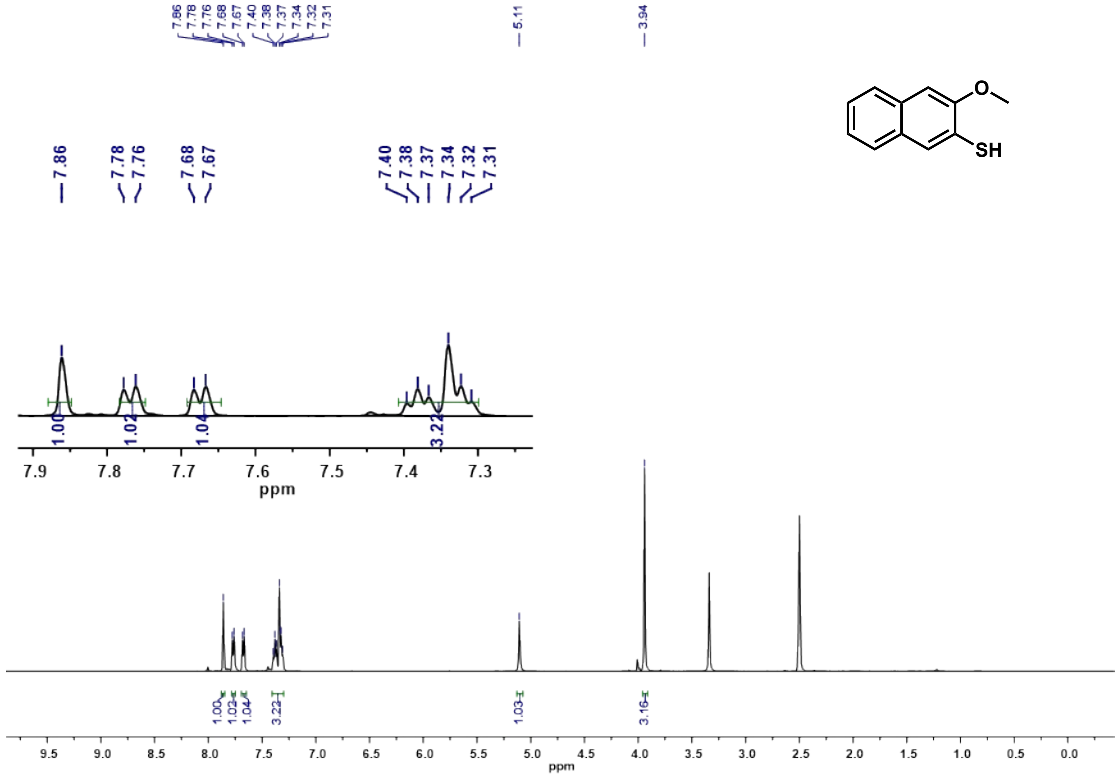

Figure S42. The ^{1}H NMR (500 MHz, 298 K) spectrum of compound **3-methoxynaphthalene-2-thiol** in DMSO-d_6.

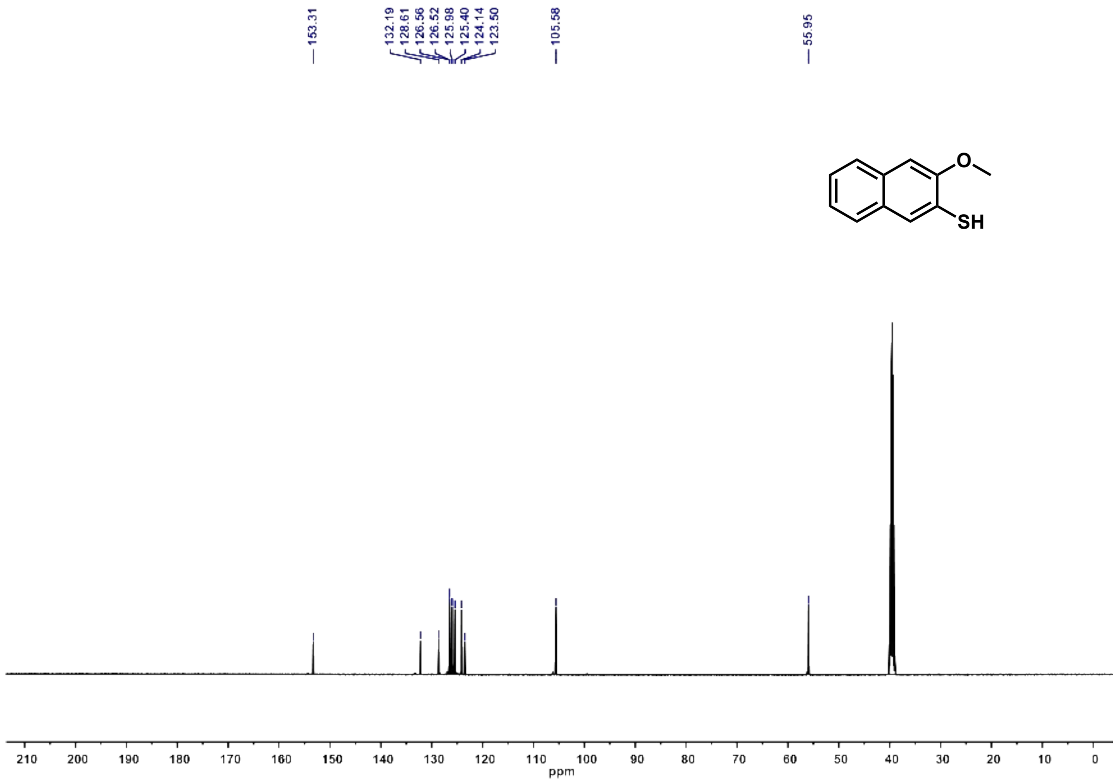

Figure S43. The ^{13}C NMR (125 MHz, 298 K) spectrum of compound **3-methoxynaphthalene-2-thiol** in DMSO-d_6.

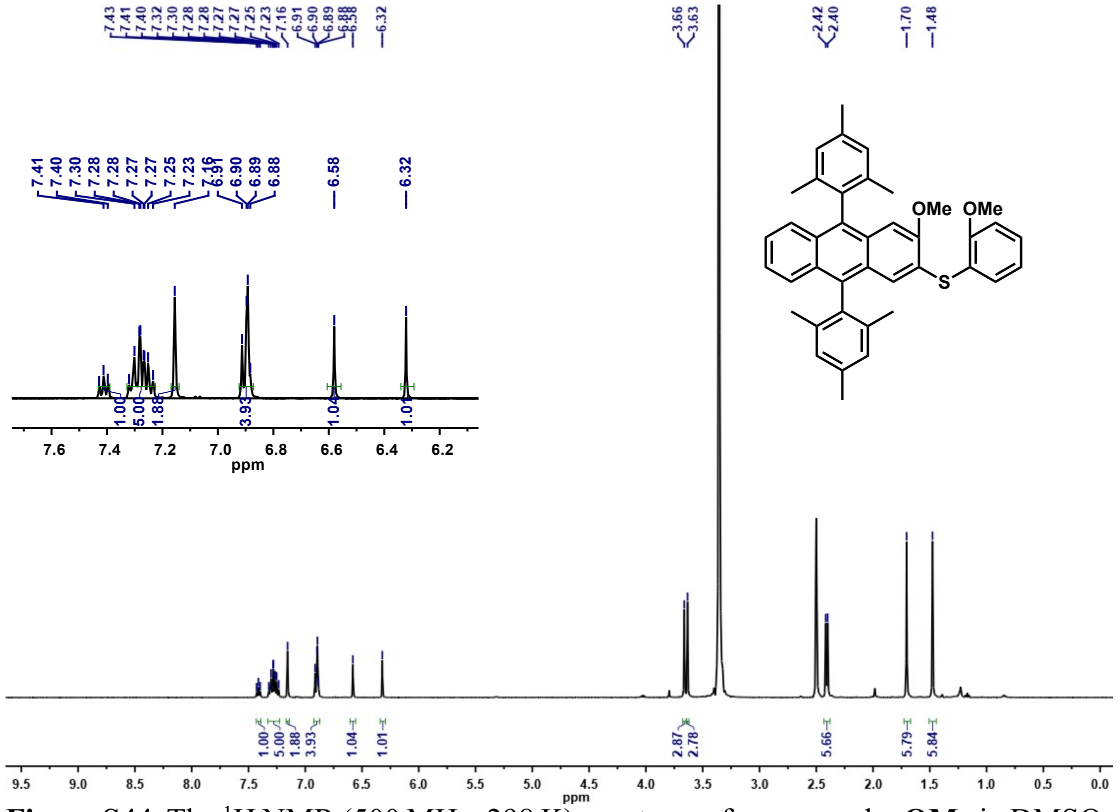

Figure S44. The ^{1}H NMR (500 MHz, 298 K) spectrum of compound **c-OMe** in DMSO-d_6.

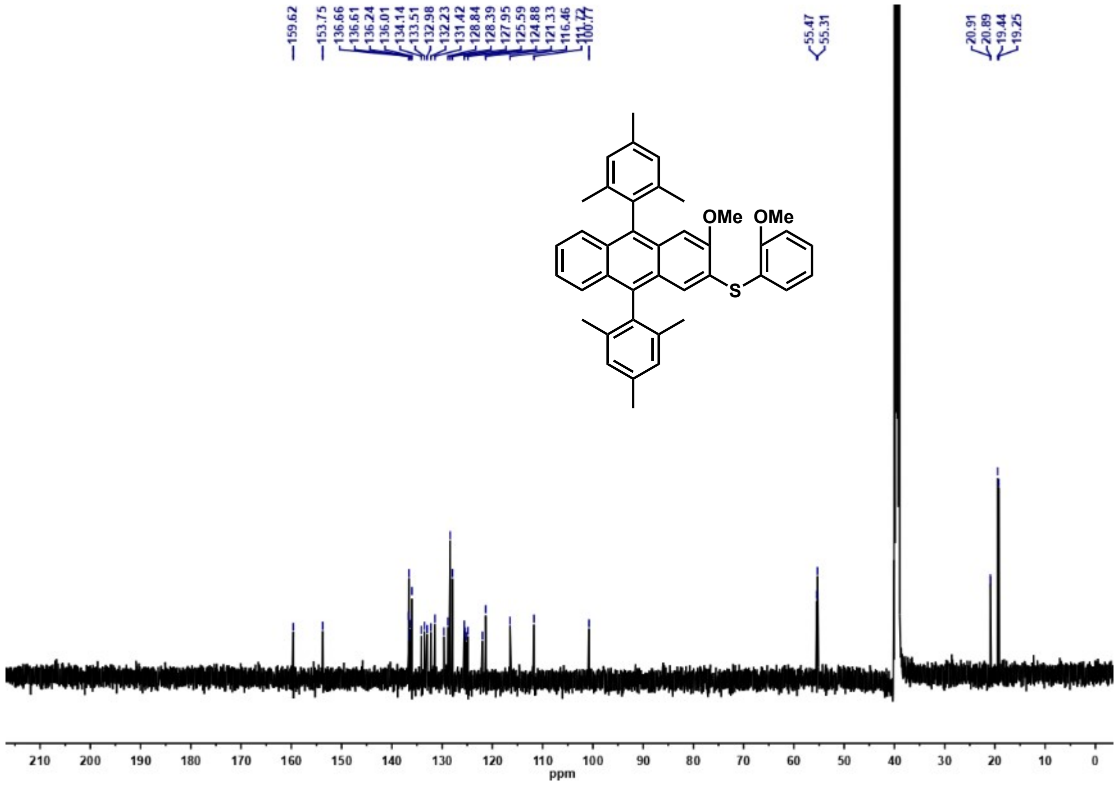

Figure S45. The ^{13}C NMR (125 MHz, 298 K) spectrum of compound **c-OMe** in DMSO-d_6.

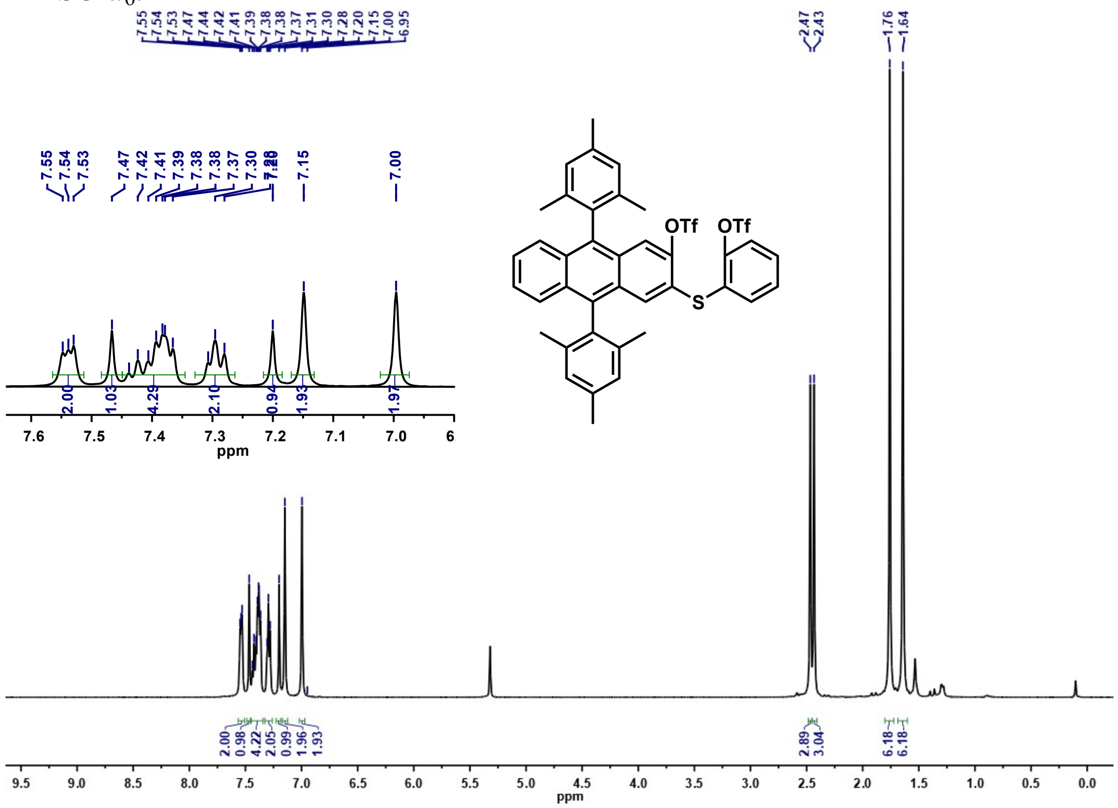

Figure S46. The ^{1}H NMR (500 MHz, 298 K) spectrum of compound **c-OTf** in CD_2Cl_2.

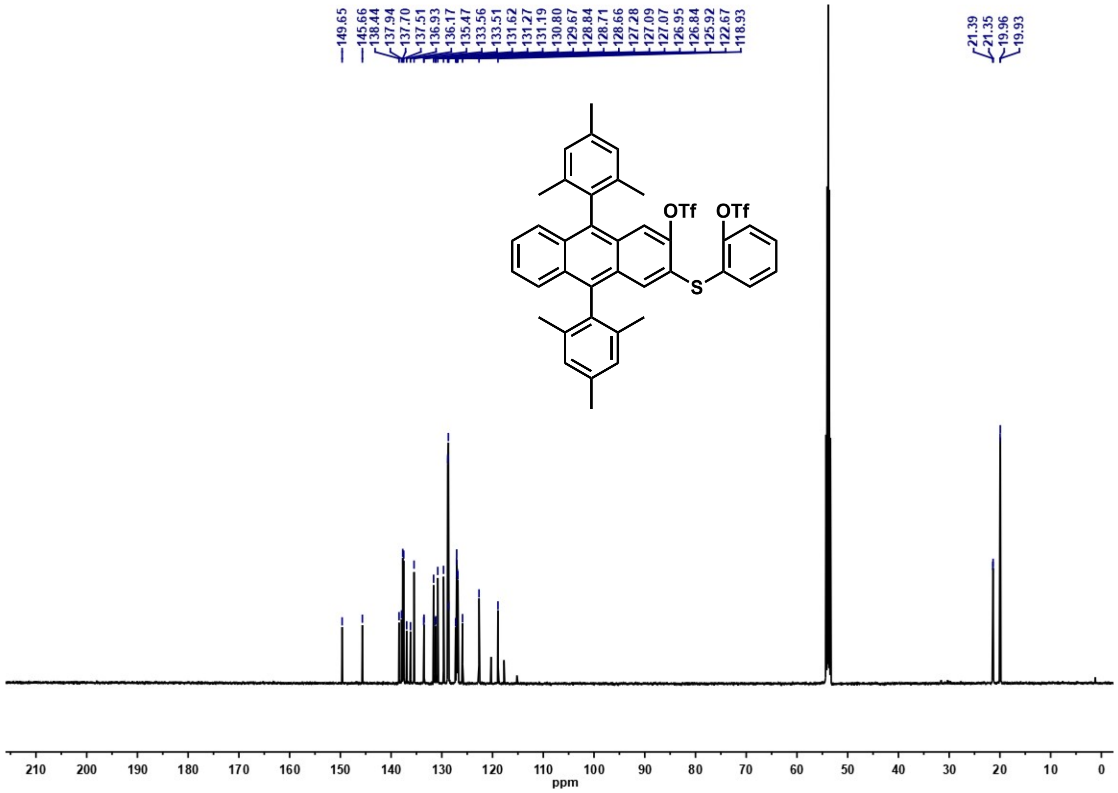

Figure S47. The ^{13}C NMR (125 MHz, 298 K) spectrum of compound **c-OTf** in CD_2Cl_2.

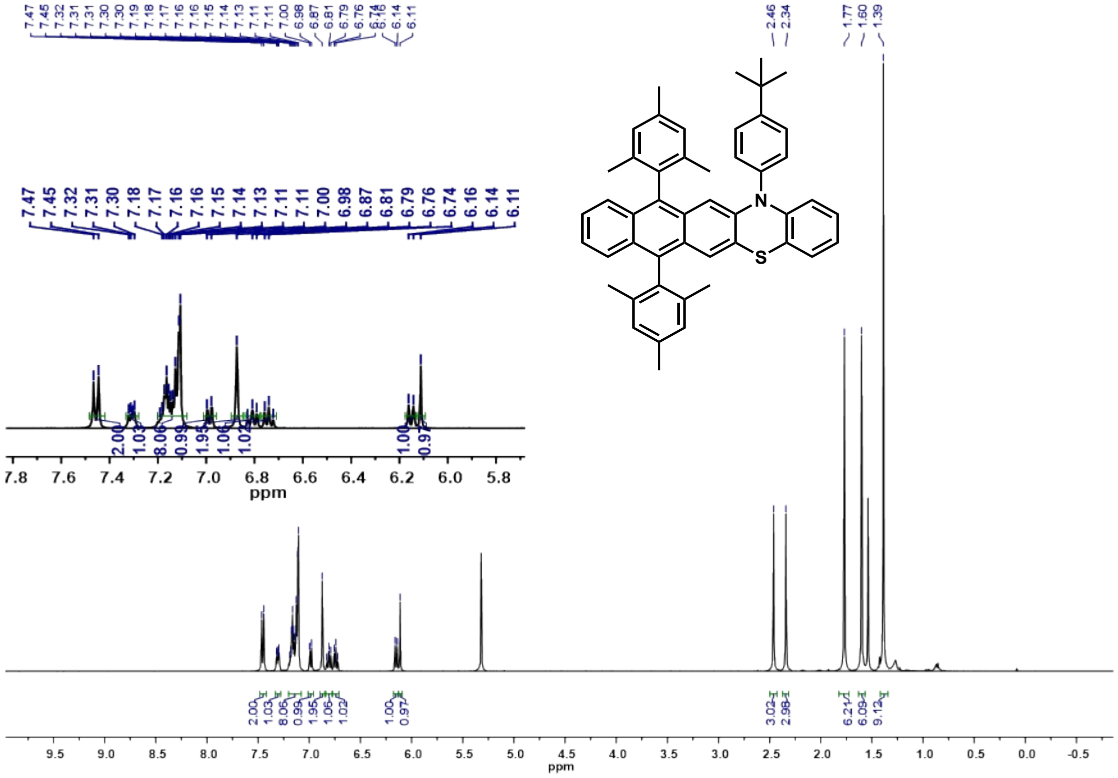

Figure S48. The ^{1}H NMR (400 MHz, 298 K) spectrum of compound **3** in CD_2Cl_2.

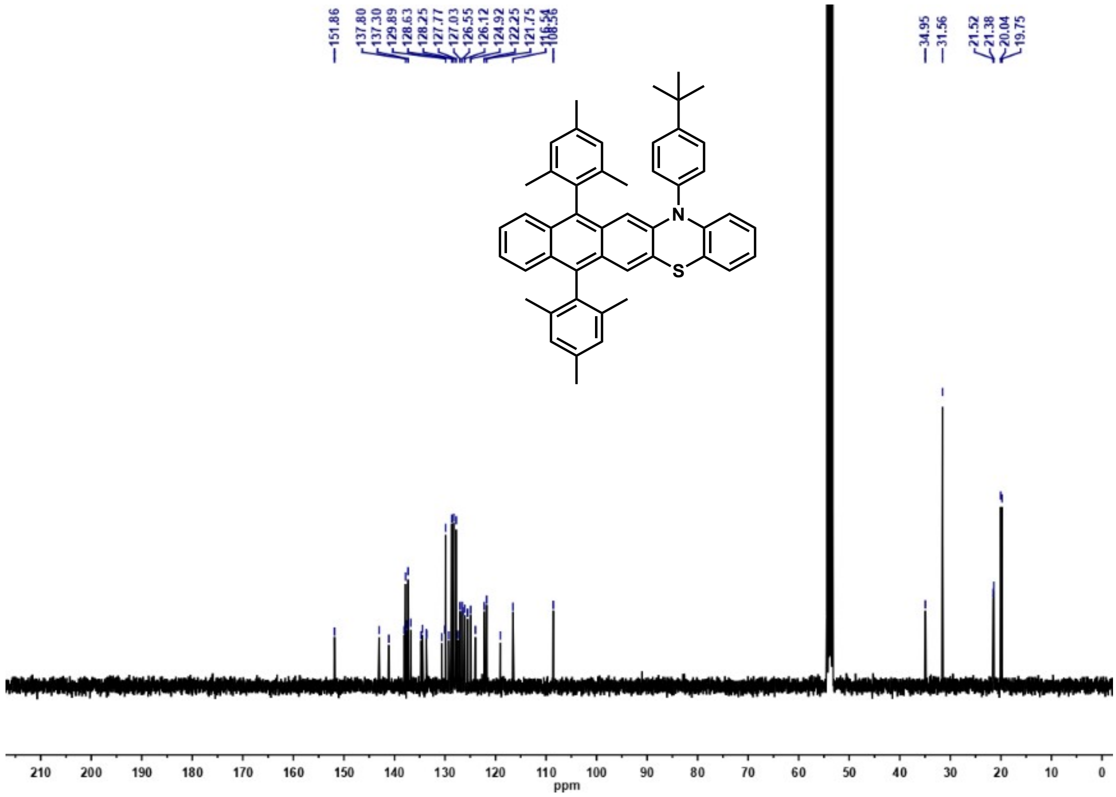

Figure S49. The ^{13}C NMR (100 MHz, 298 K) spectrum of compound **3** in CD_2Cl_2.

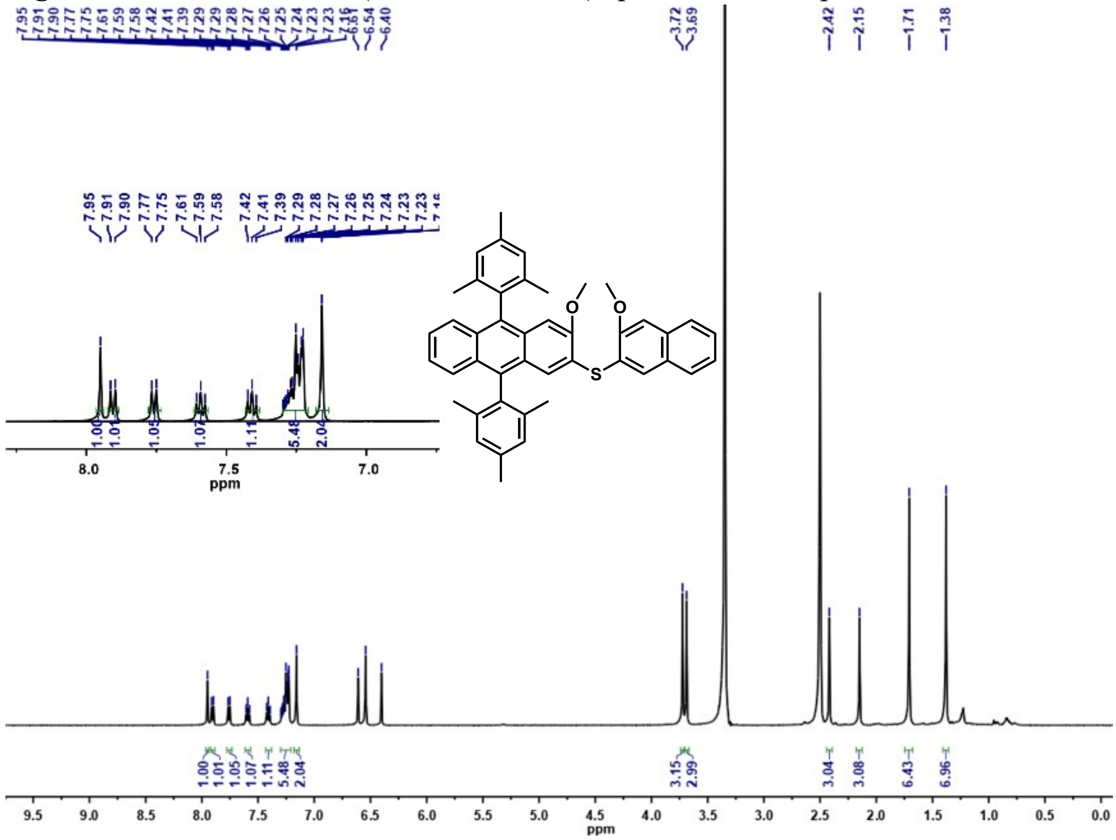

Figure S50. The ^{1}H NMR (500 MHz, 298 K) spectrum of compound **d-OMe** in DMSO-d_6.

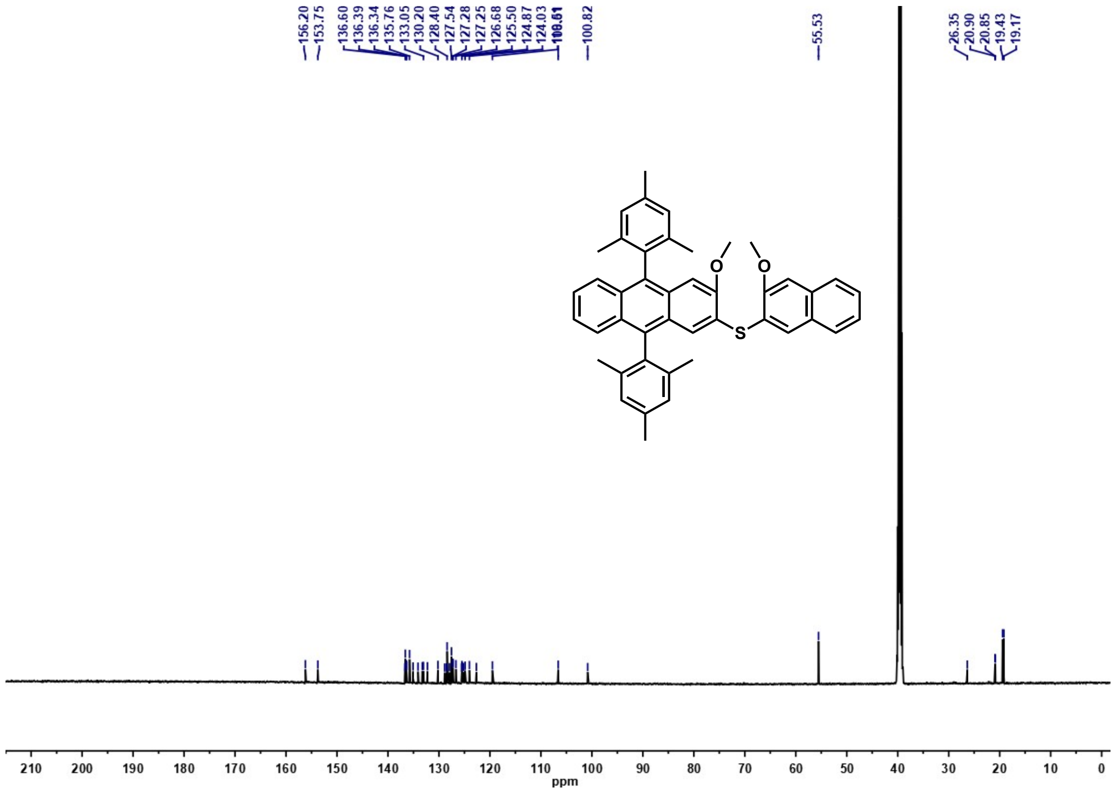

Figure S51. The ^{13}C NMR (500 MHz, 298 K) spectrum of compound **d-OMe** in DMSO-d_6.

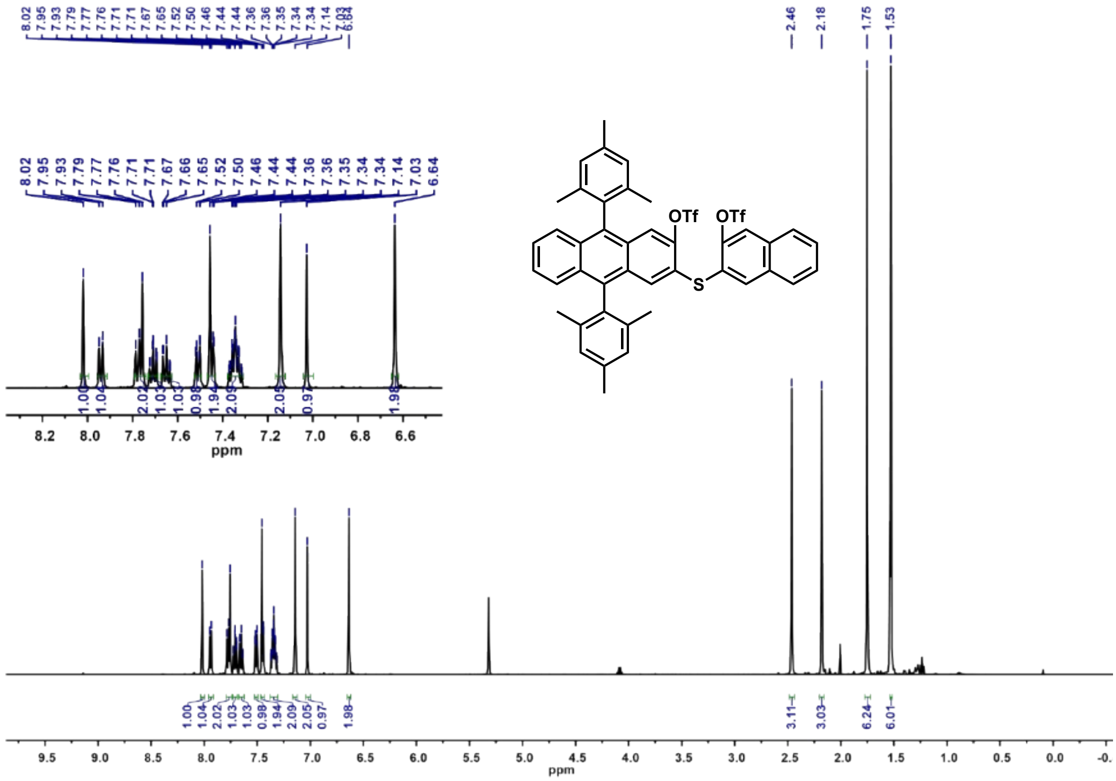

Figure S52. The ^{1}H NMR (500 MHz, 298 K) spectrum of compound **d-OTf** in CD_2Cl_2.

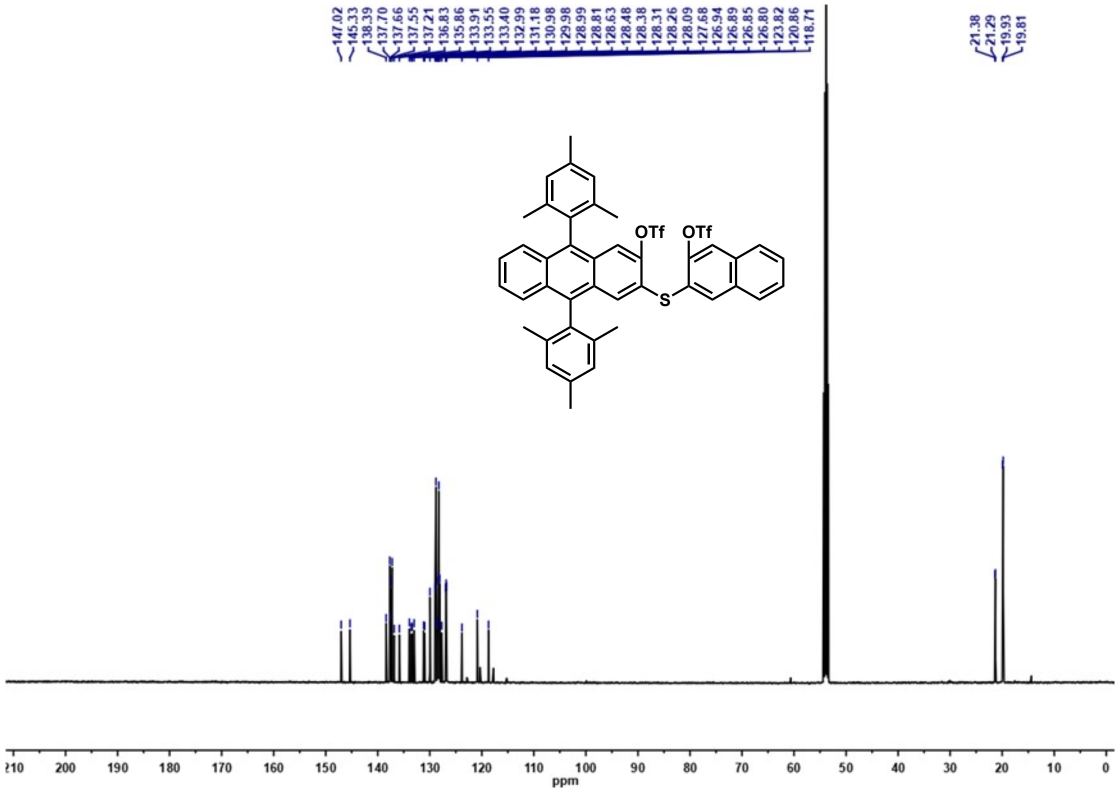

Figure S53. The ^{13}C NMR (125 MHz, 298 K) spectrum of compound **d-OTf** in CD_2Cl_2.

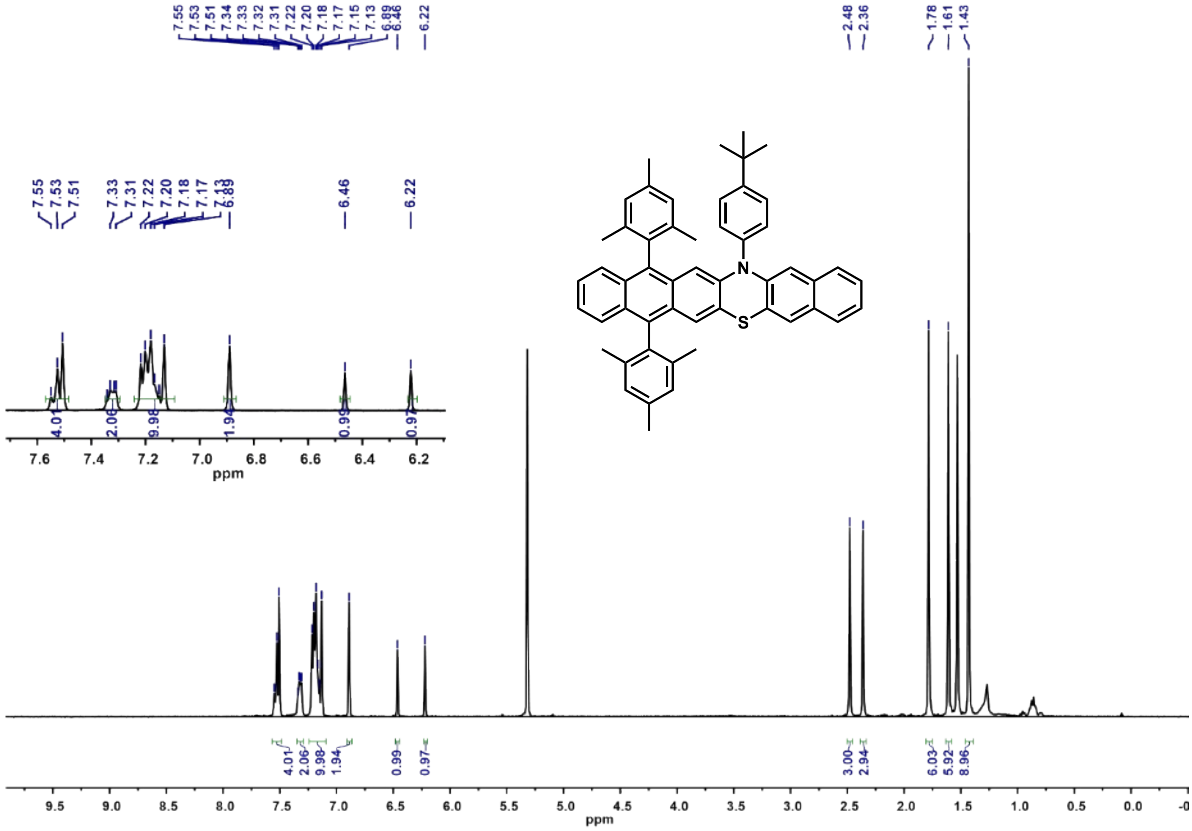

Figure S54. The ^{1}H NMR (400 MHz, 298 K) spectrum of compound **4** in CD_2Cl_2.

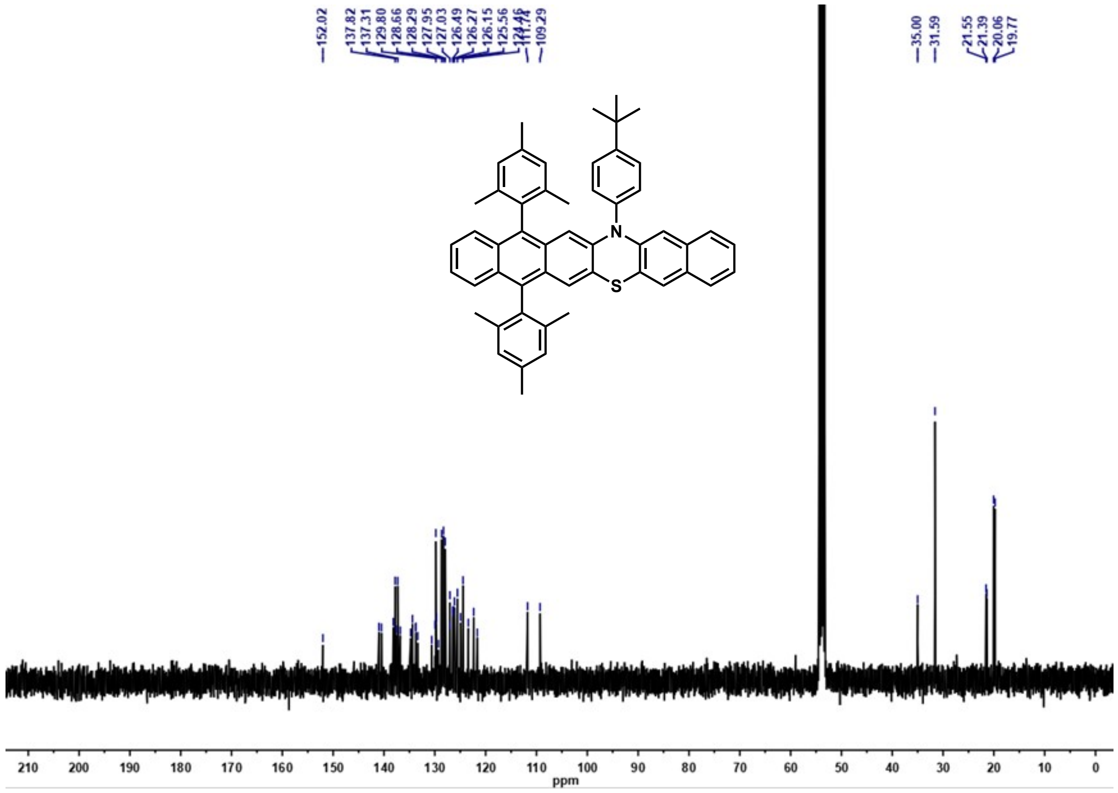

Figure S55. The ^{13}C NMR (100 MHz, 298 K) spectrum of compound **4** in CD_2Cl_2.

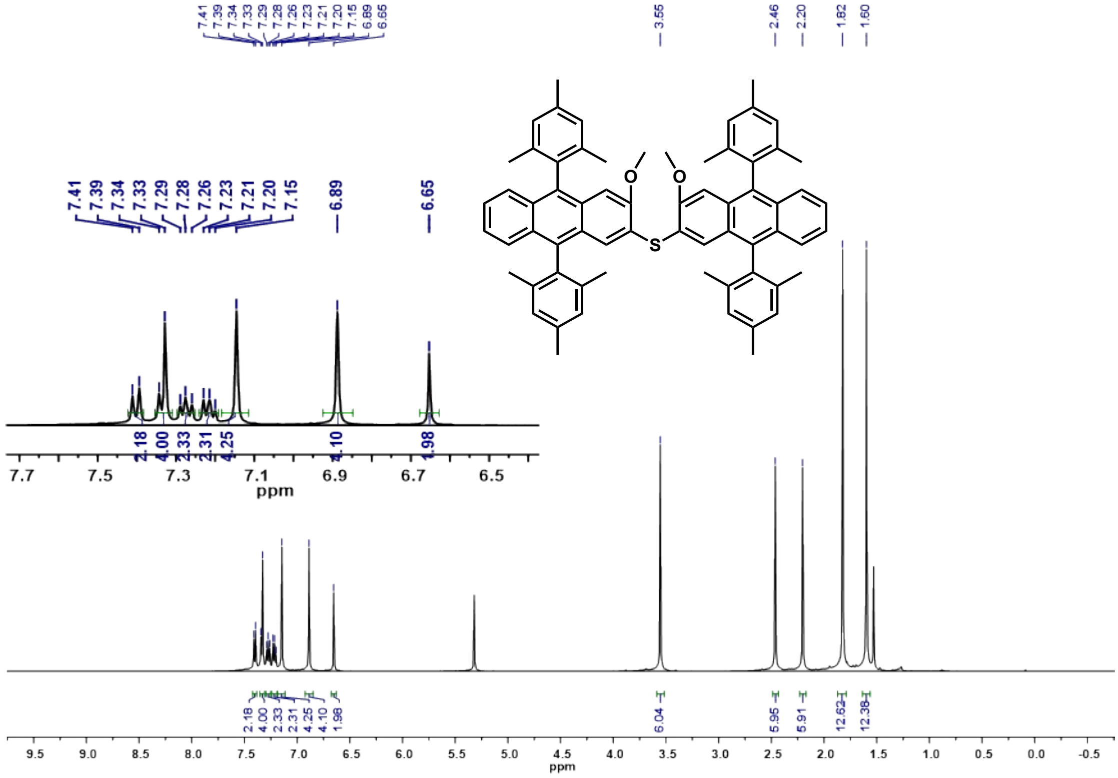

Figure S56. The ^{1}H NMR (500 MHz, 298 K) spectrum of compound **e-OMe** in CD_2Cl_2.

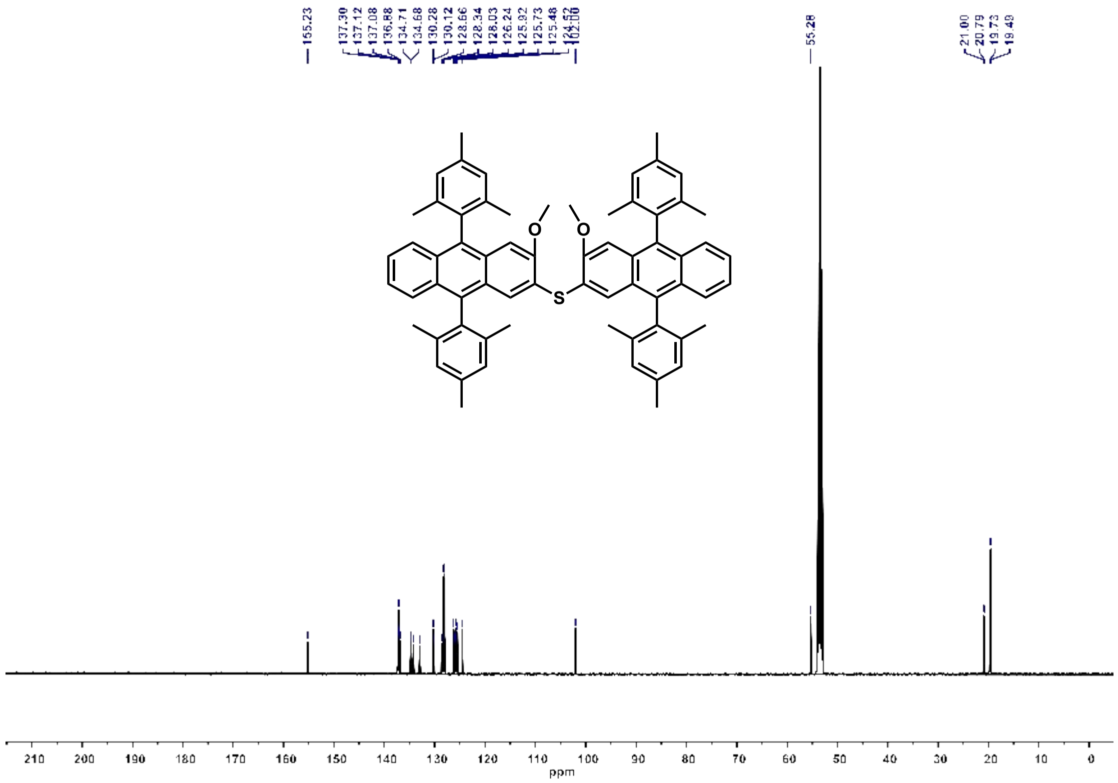

Figure S57. The ^{13}C NMR (125 MHz, 298 K) spectrum of compound **e-OMe** in CD_2Cl_2.

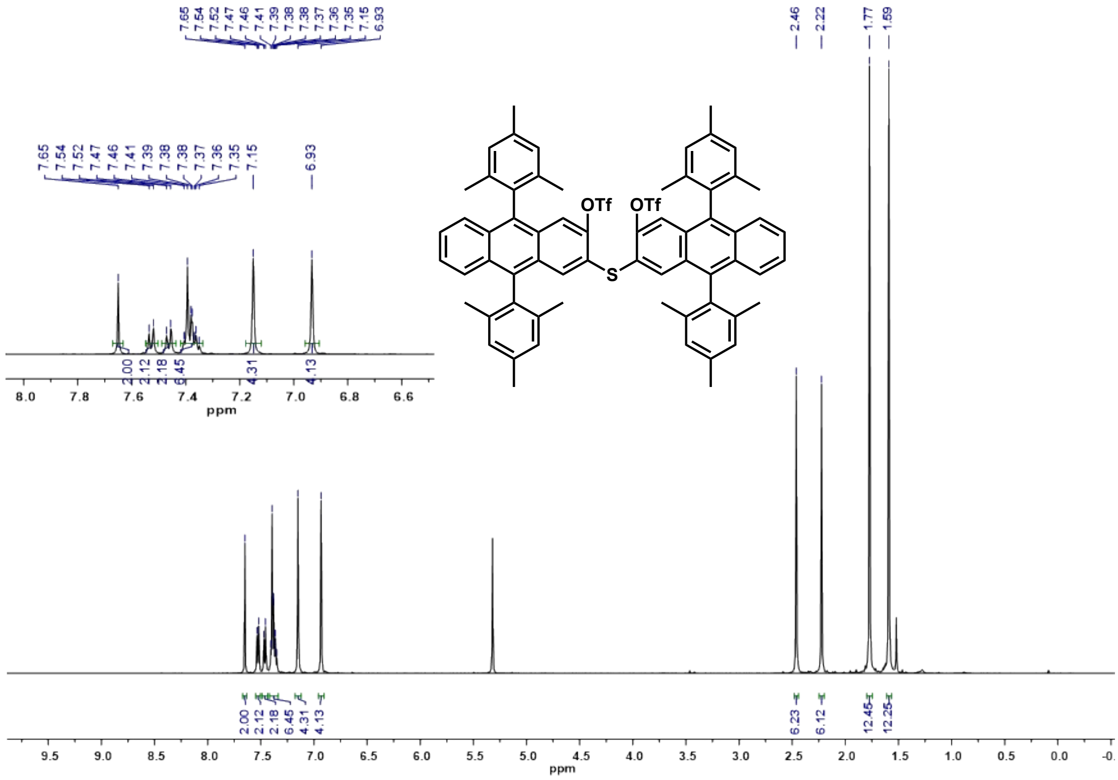

Figure S58. The ^{1}H NMR (500 MHz, 298 K) spectrum of compound **e-OTf** in CD_2Cl_2.

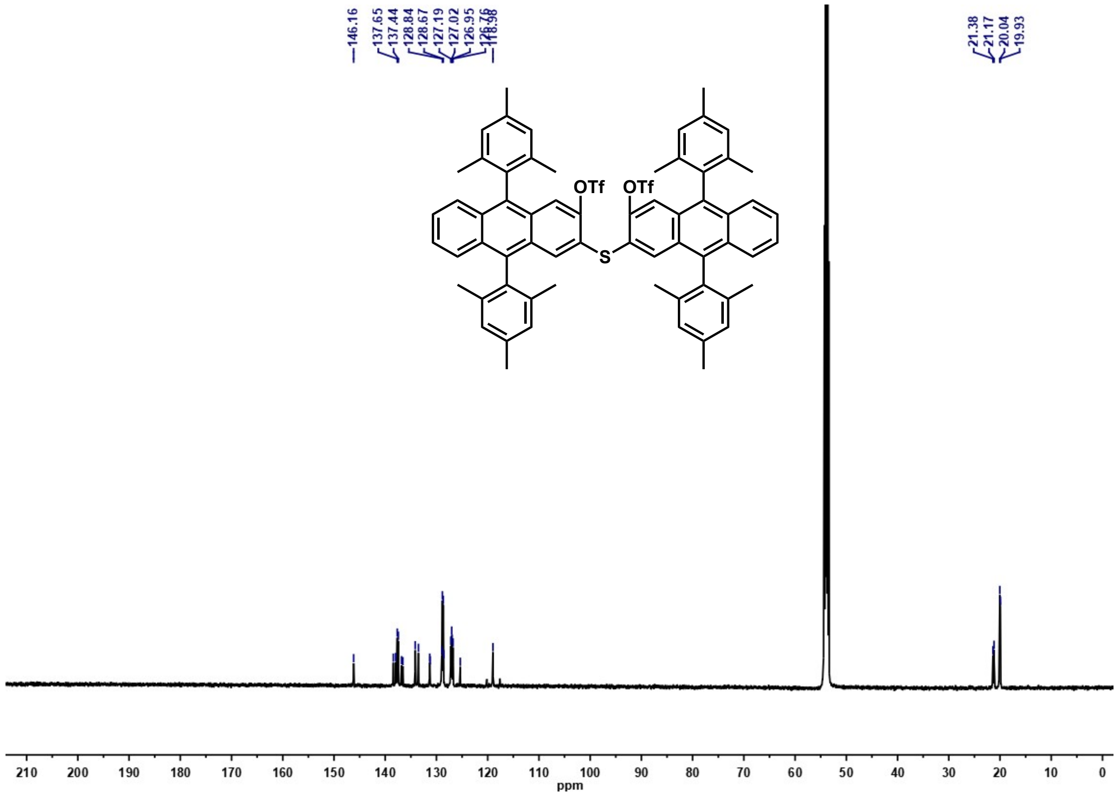

Figure S59. The ^{13}C NMR (125 MHz, 298 K) spectrum of compound **e-OTf** in CD_2Cl_2.

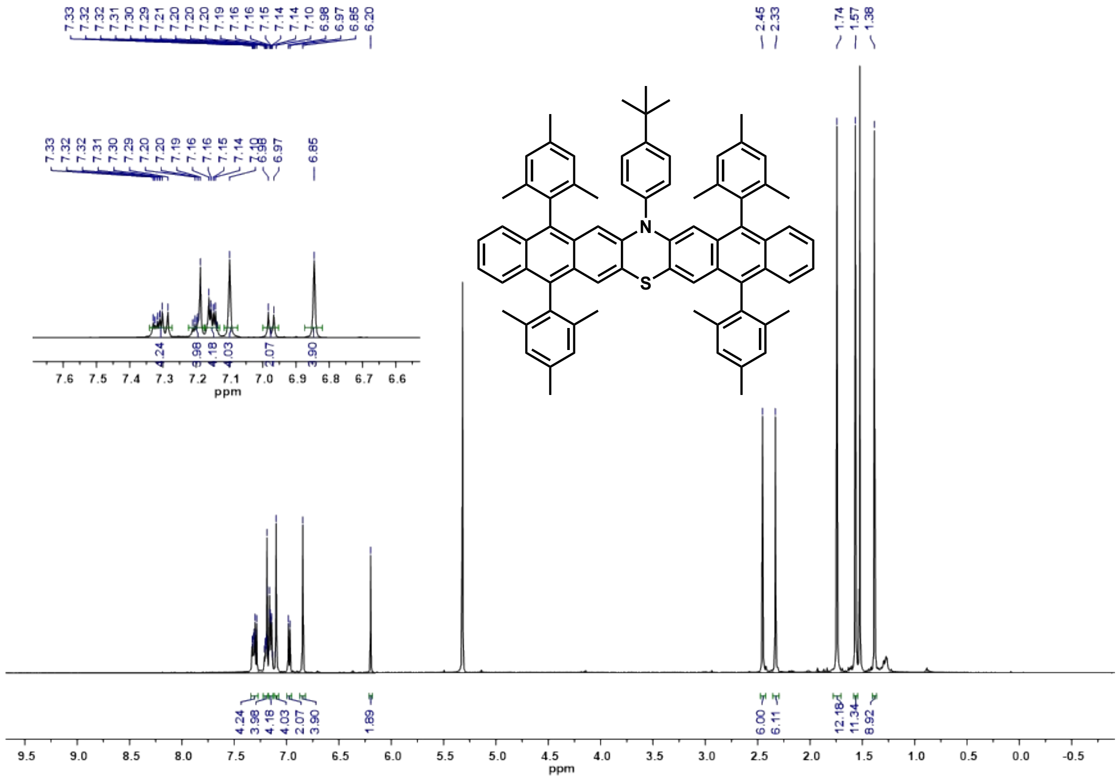

Figure S60. The ^{1}H NMR (500 MHz, 298 K) spectrum of compound **5** in CD_2Cl_2.

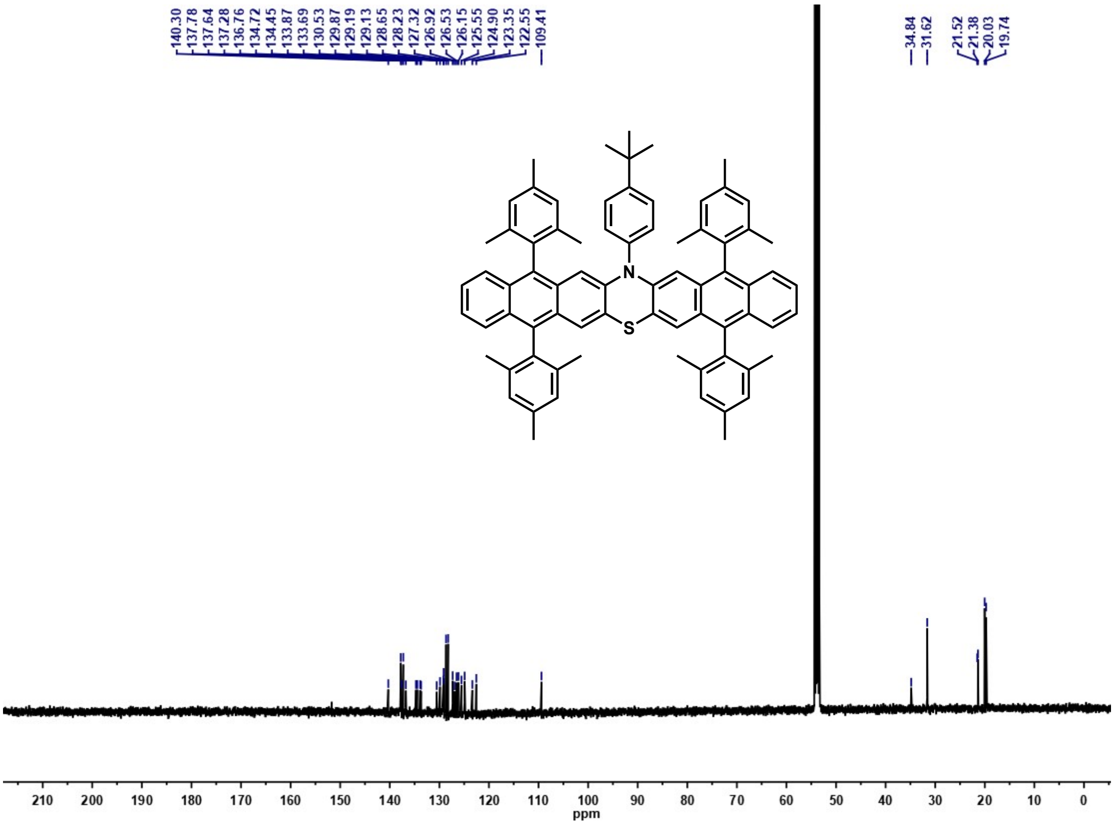

Figure S61. The ^{13}C NMR (125 MHz, 298 K) spectrum of compound **5** in CD_2Cl_2.

10.2 2D NMR spectra of new compounds

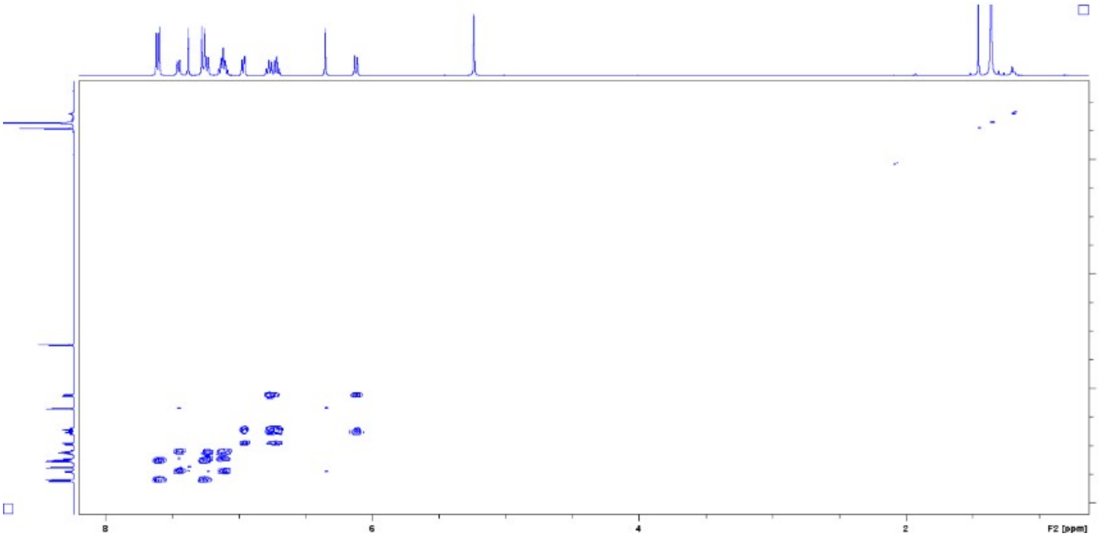

Figure S62. The 2D COSY NMR (400 MHz, CD_2Cl_2, 298 K) spectrum of **1**.

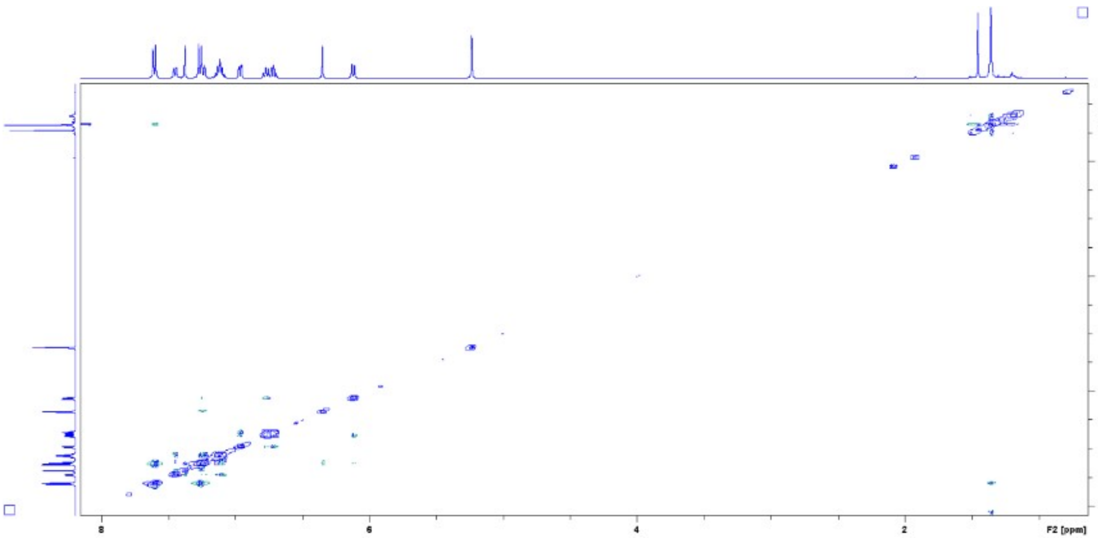

Figure S63. The 2D NOESY NMR (400 MHz, CD_2Cl_2, 298 K) spectrum of **1**.

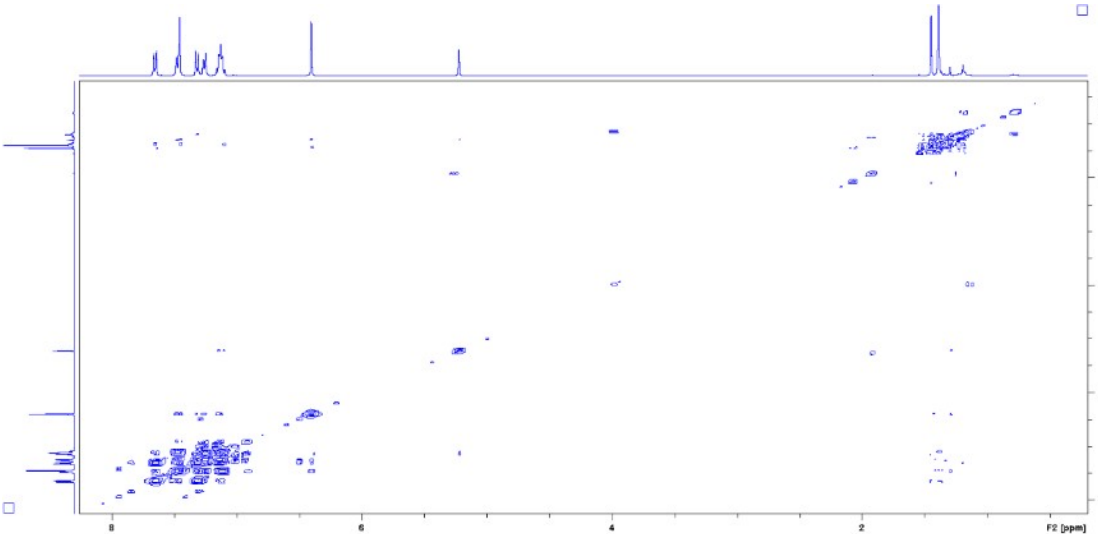

Figure S64. The 2D COSY NMR (400 MHz, CD_2Cl_2, 298 K) spectrum of **2**.

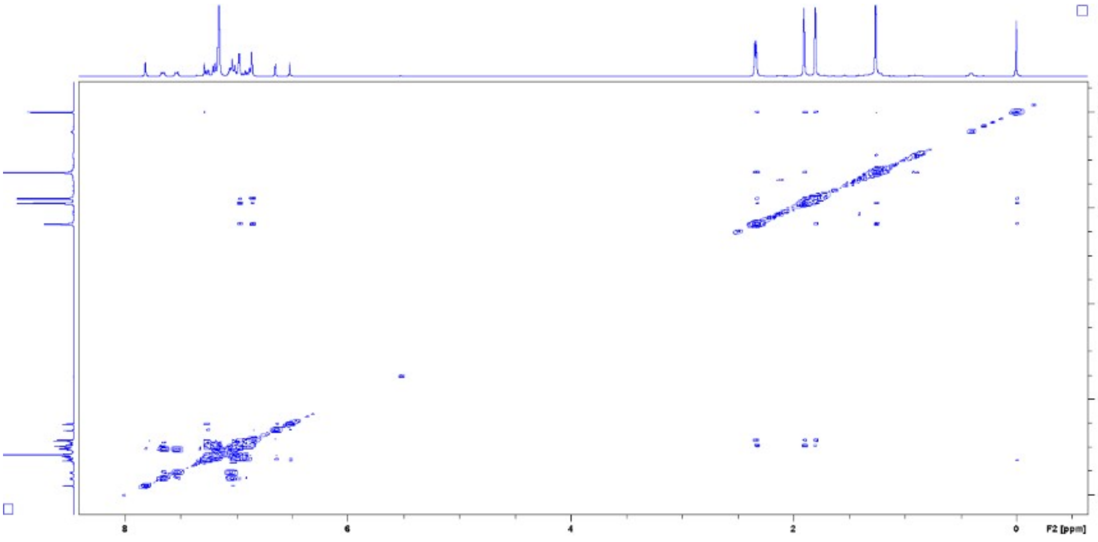

Figure S65. The 2D COSY NMR (400 MHz, C_6D_6, 298 K) spectrum of **3**.

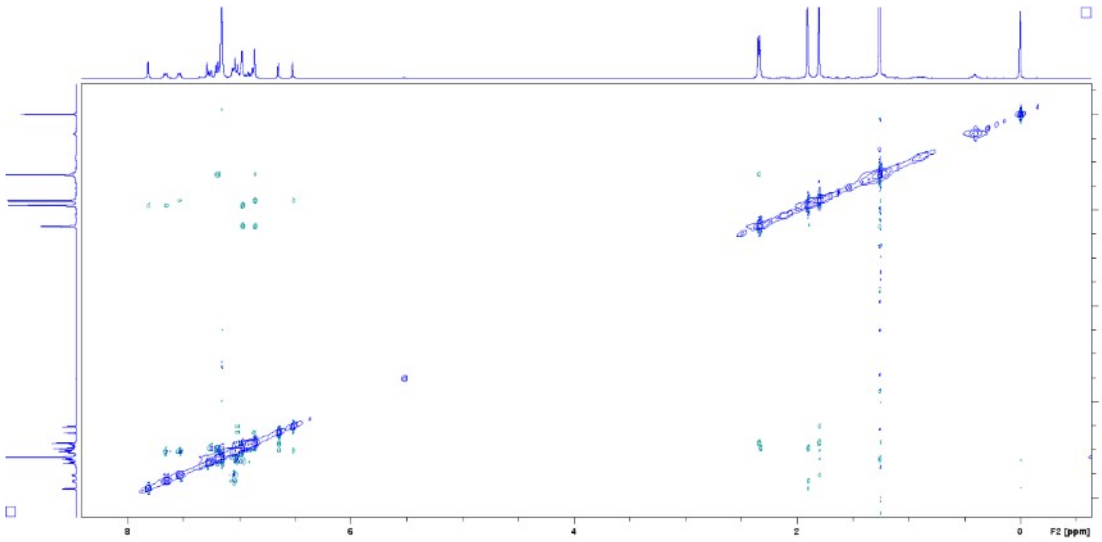

Figure S66. The 2D NOESY NMR (400 MHz, C_6D_6, 298 K) spectrum of **3**.

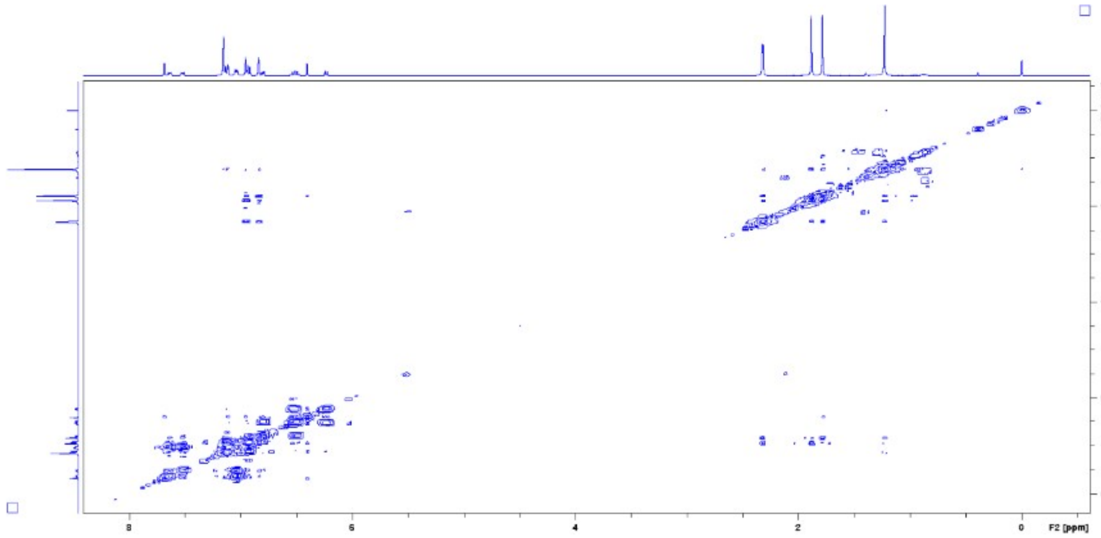

Figure S67. The 2D COSY NMR (400 MHz, C_6D_6, 298 K) spectrum of **4**.

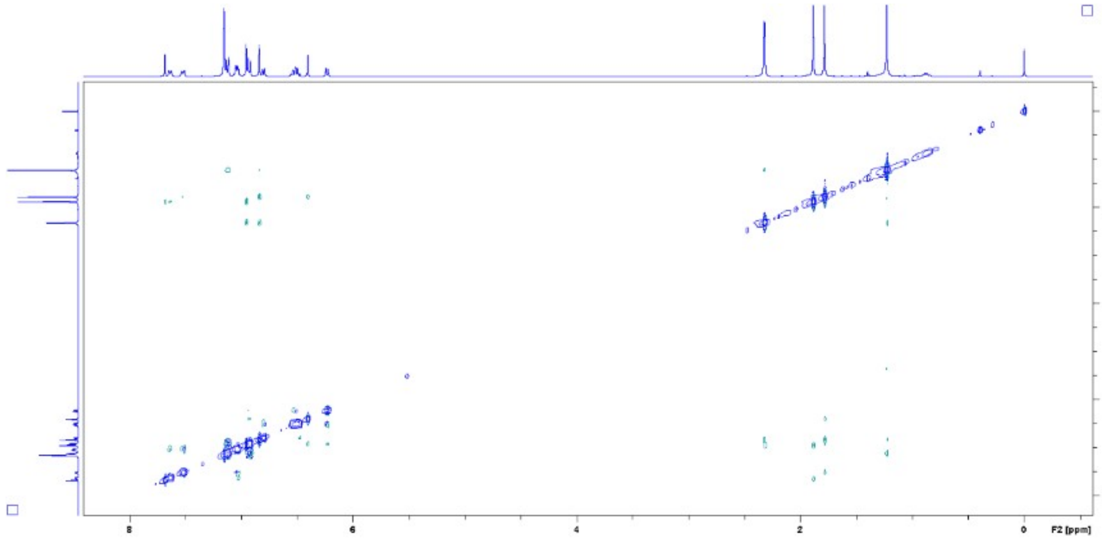

Figure S68. The 2D NOESY NMR (400 MHz, C_6D_6, 298 K) spectrum of **4**.

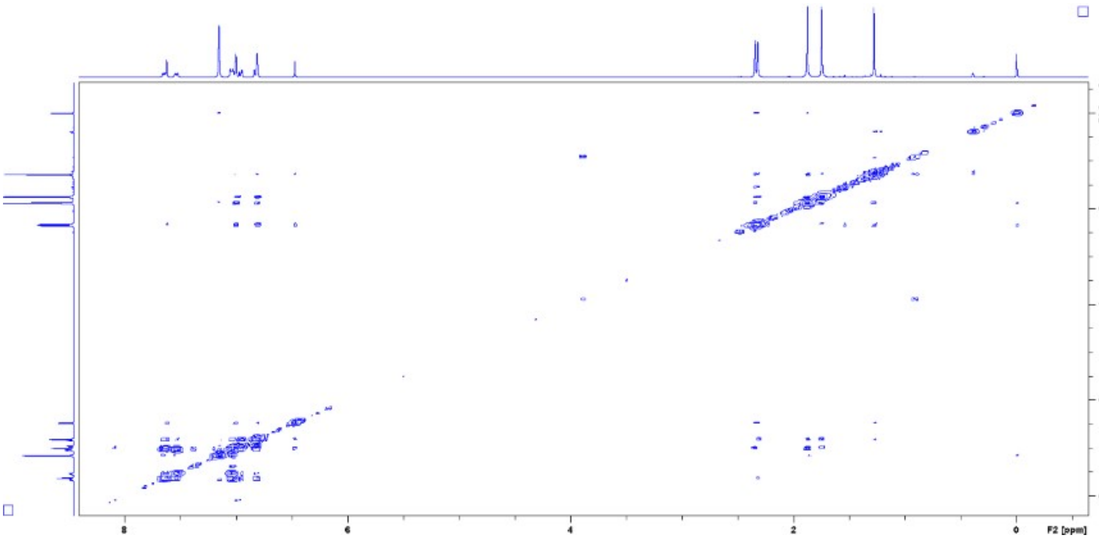

Figure S69. The 2D COSY NMR (400 MHz, C_6D_6, 298 K) spectrum of **5**.

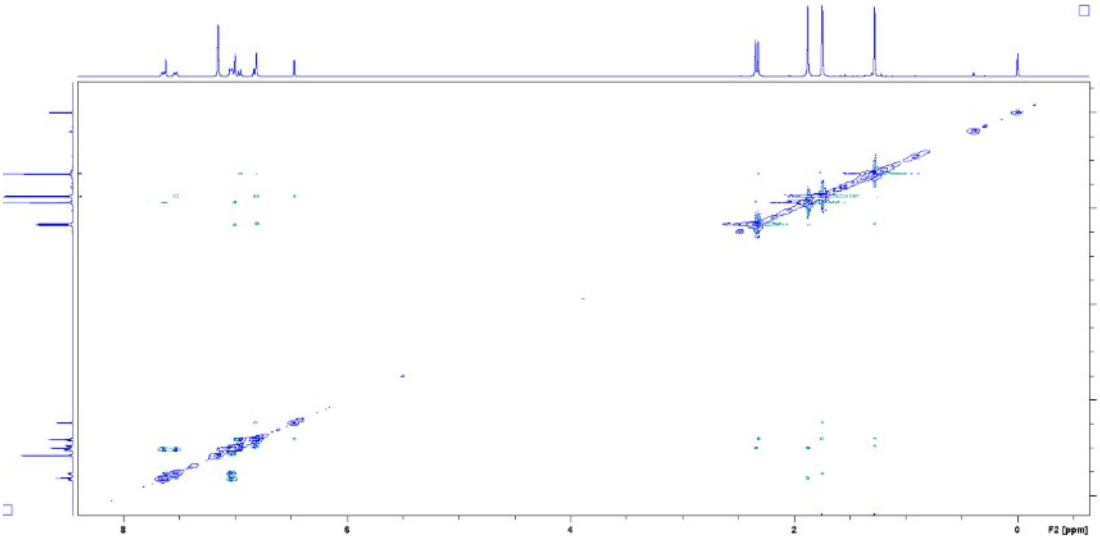

Figure S70. The 2D NOESY NMR (400 MHz, C_6D_6, 298 K) spectrum of **5**.

10.3 In situ ^{1}H NMR spectra of imines

10.3.1 In situ ^{1}H NMR spectra in Table 2.

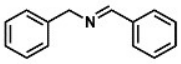

(**1a**) ^{1}H NMR (500 MHz, C_6D_6) δ (ppm) 7.99 (s, 1H), 7.75 (d, *J* = 6.9 Hz, 2H), 7.31 (d, *J* = 7.5 Hz, 2H), 7.20 (t, *J* = 7.5 Hz, 2H), 7.15-7.05 (m, 4H), 4.60 (s, 2H).

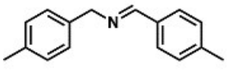 (**1b**) ^{1}H NMR (500 MHz, C_6D_6) δ (ppm) 8.07 (s, 1H), 7.72 (d, *J* = 7.2 Hz, 2H), 7.28 (d, *J* = 6.9 Hz, 2H), 7.04 (d, *J* = 6.8 Hz, 2H), 6.96 (d, *J* = 6.9 Hz, 2H), 2.14 (s, 3H), 2.04 (s, 3H).

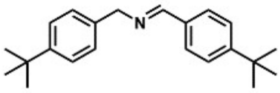 (**1c**) ^{1}H NMR (400 MHz, C_6D_6) δ (ppm) 8.20 (s, 1H), 7.75 (d, *J* = 8.3 Hz, 2H), 7.337.22 (m, 6H), 4.66 (s, 2H), 1.18 (s, 9H), 1.13 (s, 9H).

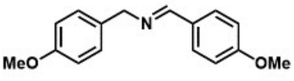

(**1d**)^{1}H NMR (500 MHz, C_6D_6) δ (ppm) 8.06 (s, 1H), 7.75 (d, *J* = 6.6 Hz, 2H), 7.27 (d, *J* = 6.3 Hz, 2H), 6.84 (d, *J* = 6.4 Hz, 2H), 6.75 (d, *J* = 6.6 Hz, 2H), 4.65 (s, 2H), 3.32 (s, 3H), 3.22 (s, 3H). Some product peaks overlap with solvent peak.

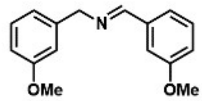

(**1e**)^{1}H NMR (500 MHz, C_6D_6) δ (ppm) 8.19 (s, 1H), 7.42 (s, 1H), 7.28 (d, *J* = 7.5 Hz, 1H), 7.18-7.09 (m, 2H), 6.93 (s, 1H), 6.89-6.84 (m, 2H), 6.70 (dd, *J* = 8.2, 2.3 Hz, 1H), 4.62 (s, 2H), 3.46 (s, 3H), 3.45 (s, 3H).

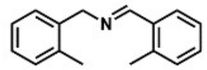 (**1f**) ^{1}H NMR (500 MHz, C_6D_6) δ (ppm) 8.52 (s, 1H), 7.91 (d, *J* = 7.5 Hz, 1H), 7.28-7.25 (m, 1H), 7.13-7.02 (m, 5H), 6.99 (d, *J* = 7.3 Hz, 1H), 4.62 (s, 2H). Some product peaks overlap with solvent peak.

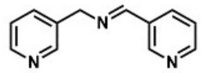 (**1g**) ^{1}H NMR (500 MHz, C_6D_6) δ (ppm) 8.81 (d, *J* = 1.7 Hz, 1H), 8.67 (s, 1H), 8.50 (dd, *J* = 4.7, 1.2 Hz, 1H), 8.47 (dd, *J* = 4.7, 1.6 Hz, 1H), 7.84 (dt, *J* = 7.9, 1.8 Hz, 1H), 7.64 (s, 1H), 7.28 (d, *J* = 7.8 Hz, 1H), 6.78 (dd, *J* = 7.8, 4.8 Hz, 1H), 6.69 (dd, *J* = 7.8, 4.7 Hz, 1H), 4.26 (s, 2H).

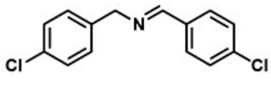

(**1h**) ^{1}H NMR (500 MHz, C_6D_6) δ (ppm) 8.17 (s, 1H), 7.63 (d, *J* = 8.2 Hz, 2H), 7.27 (d, *J* = 8.1 Hz, 2H), 7.21-7.14 (m, 4H), 4.53 (s, 2H).

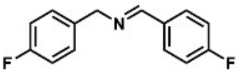

(**1i**) ^{1}H NMR (500 MHz, C_6D_6) δ (ppm) 7.78 (s, 1H), 7.54-7.46 (m, 2H), 7.07-7.02 (m, 2H), 6.89-6.83 (m, 2H), 6.78-6.73 (m, 2H), 4.41 (s, 2H).

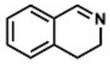

(**1j**) ^{1}H NMR (500 MHz, C_6D_6) δ (ppm) 8.26 (s, 1H), 7.13-7.09 (m, 1H), 7.08-7.01 (m, 2H), 6.88 (d, J = 7.3 Hz, 1H), 3.57-3.49 (m, 2H), 2.41-2.35 (m, 2H).

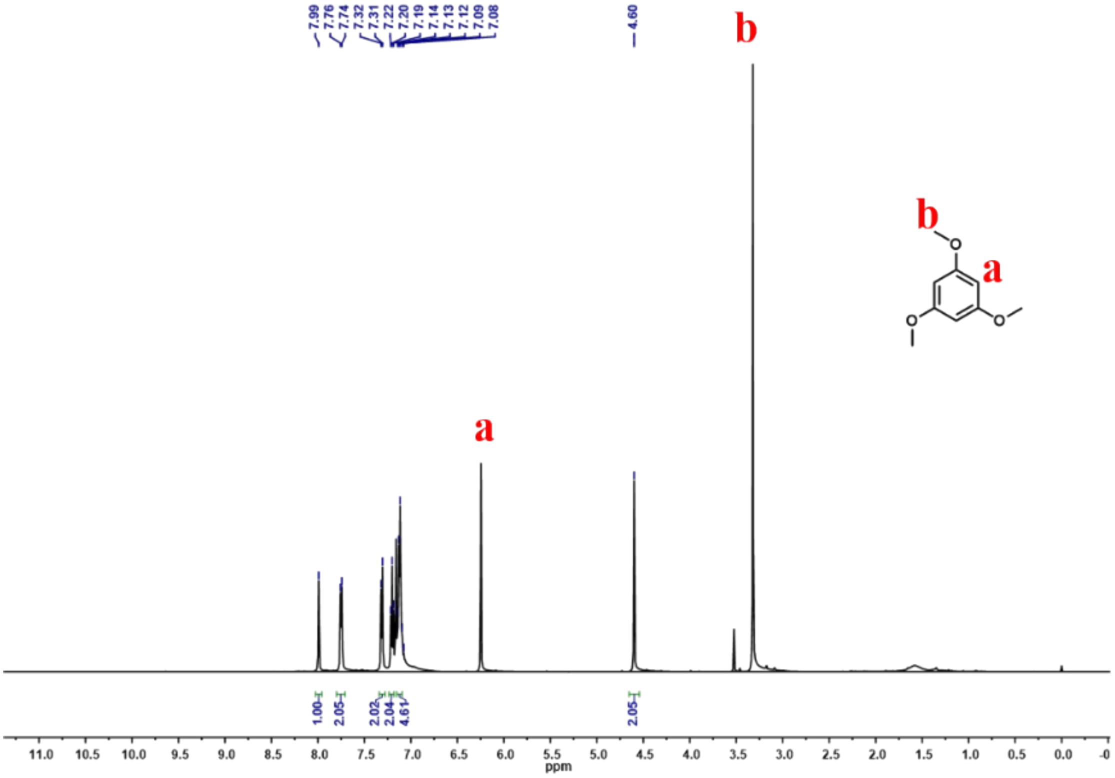

Figure S71. The ^{1}H NMR (500 MHz, 298 K) spectrum of compound **1a** in C_6D_6.

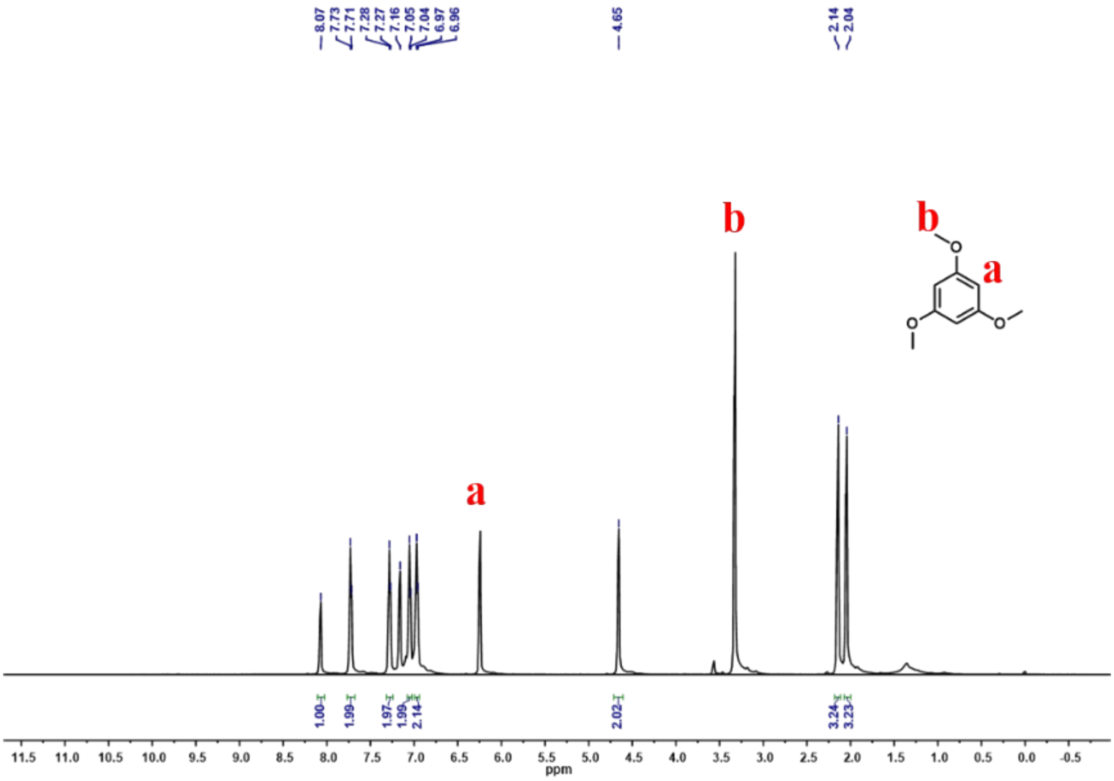

Figure S72. The ^{1}H NMR (500 MHz, 298 K) spectrum of compound **1b** in C_6D_6.

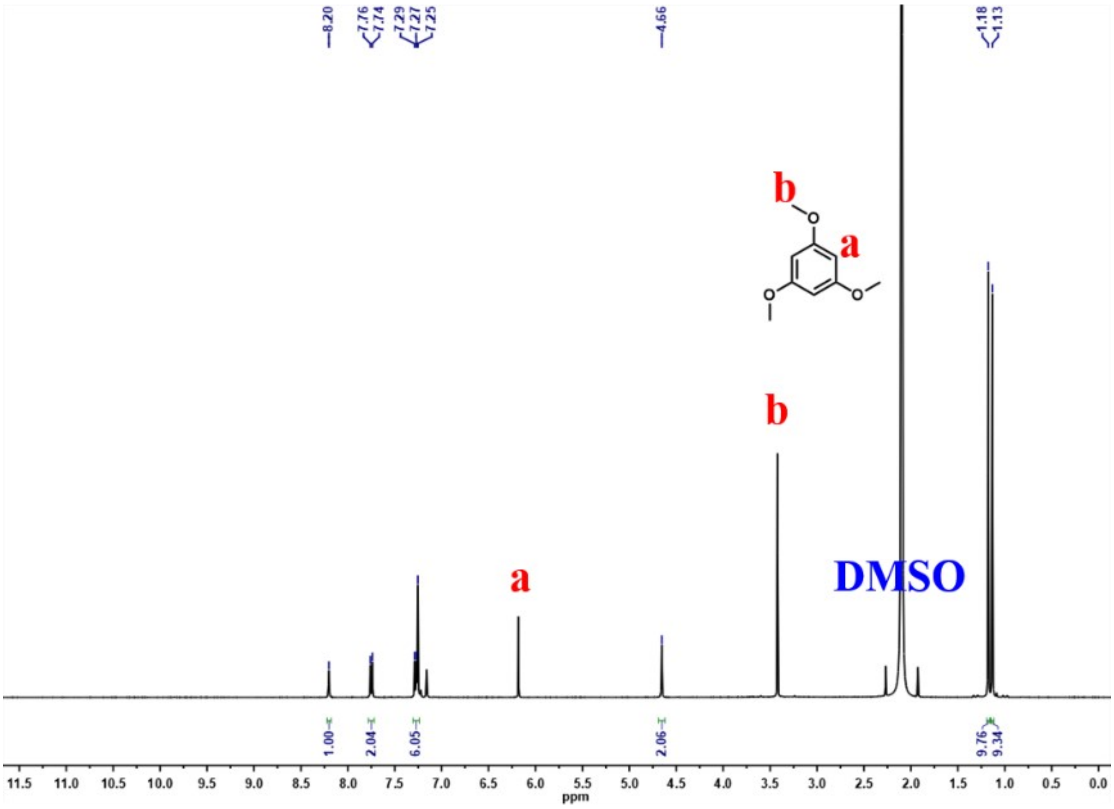

Figure S73. The ^{1}H NMR (400 MHz, 298 K) spectrum of compound **1c** in C_6D_6.

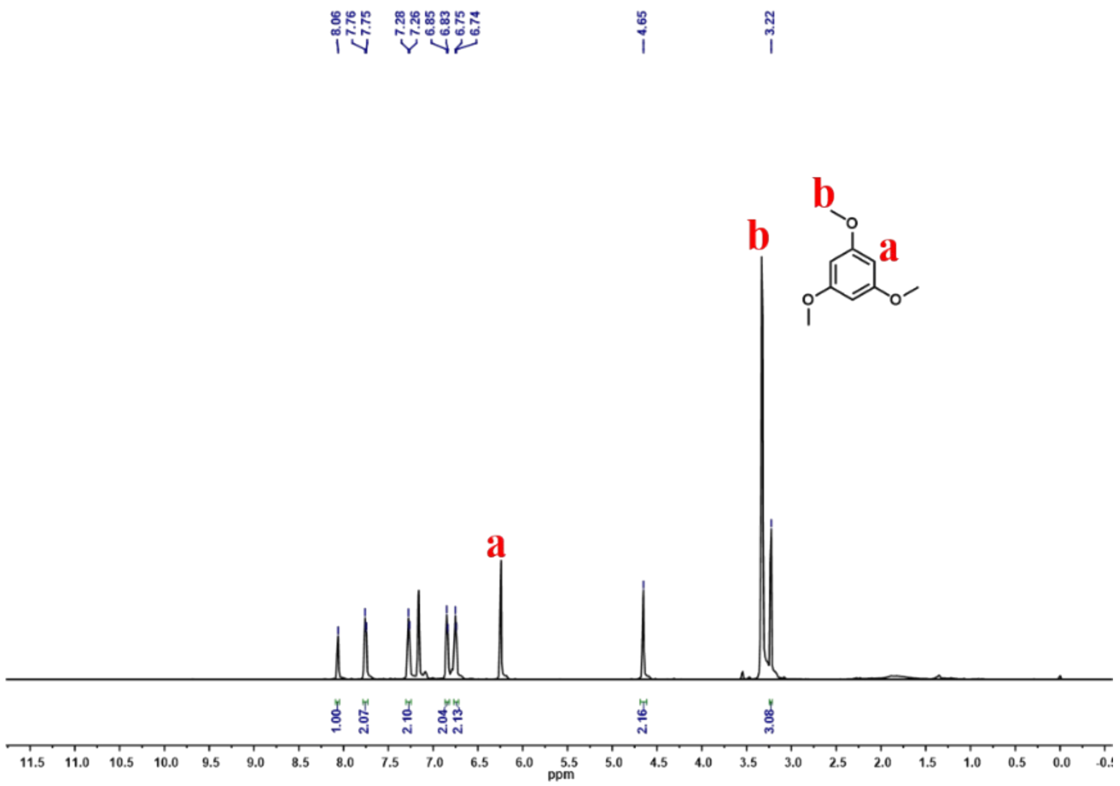

Figure S74. The ^{1}H NMR (500 MHz, 298 K) spectrum of compound **1d** in C_6D_6.

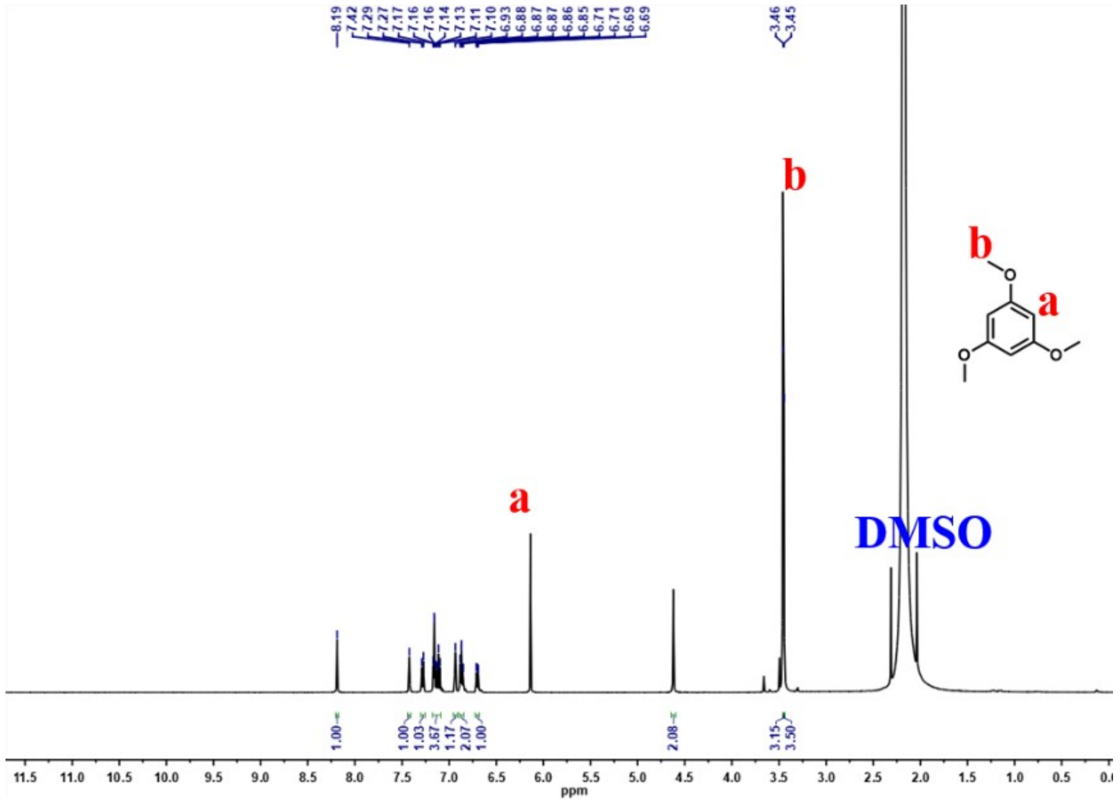

Figure S75. The ^{1}H NMR (500 MHz, 298 K) spectrum of compound **1e** in C_6D_6.

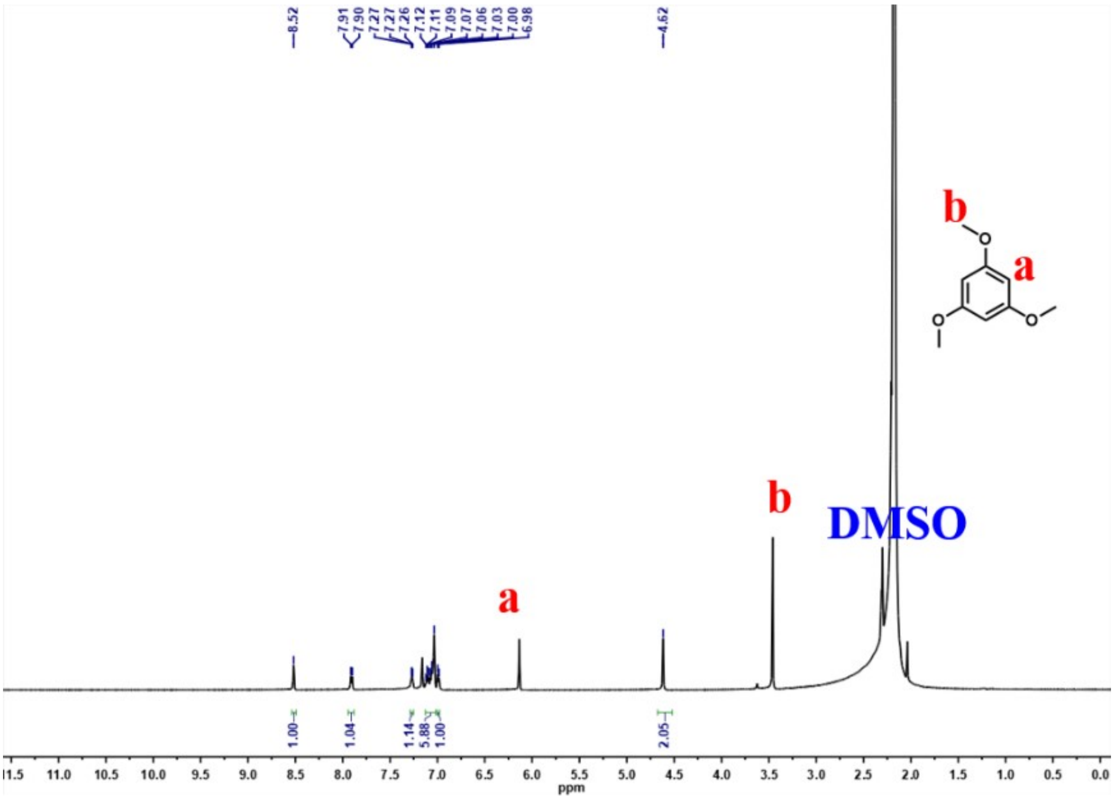

Figure S76. The ^{1}H NMR (500 MHz, 298 K) spectrum of compound **1f** in C_6D_6.

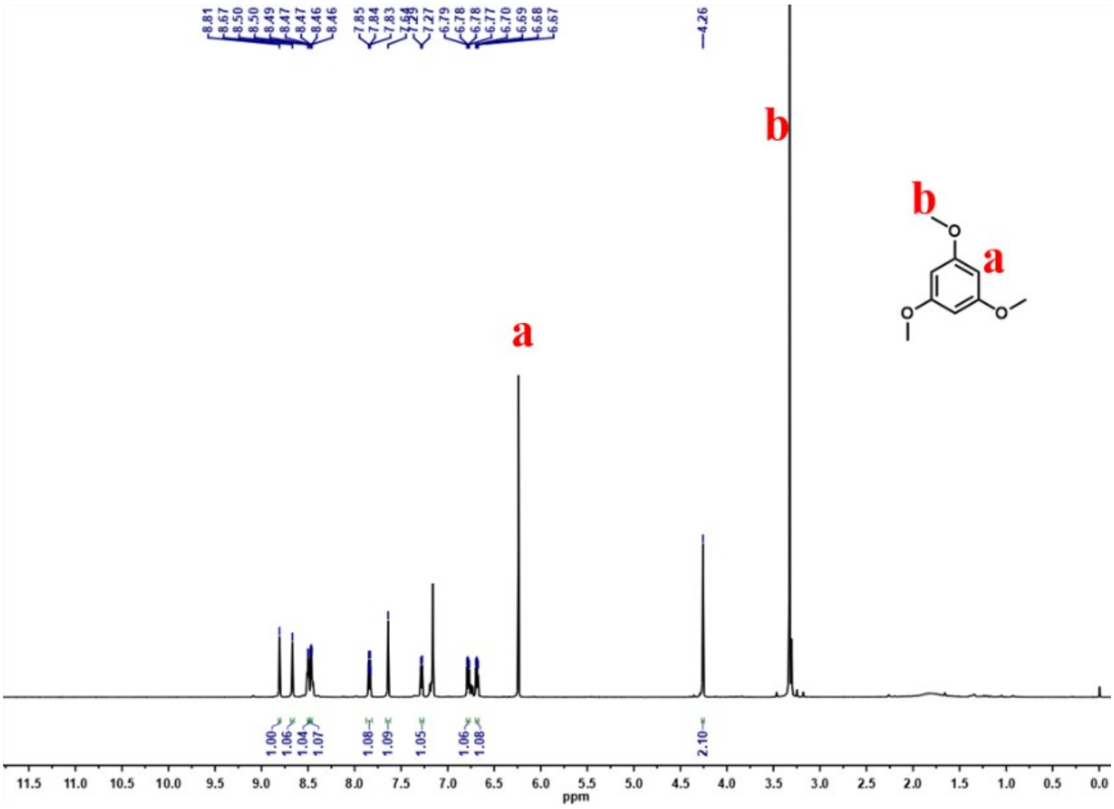

Figure S77. The ^{1}H NMR (500 MHz, 298 K) spectrum of compound **1g** in C_6D_6.

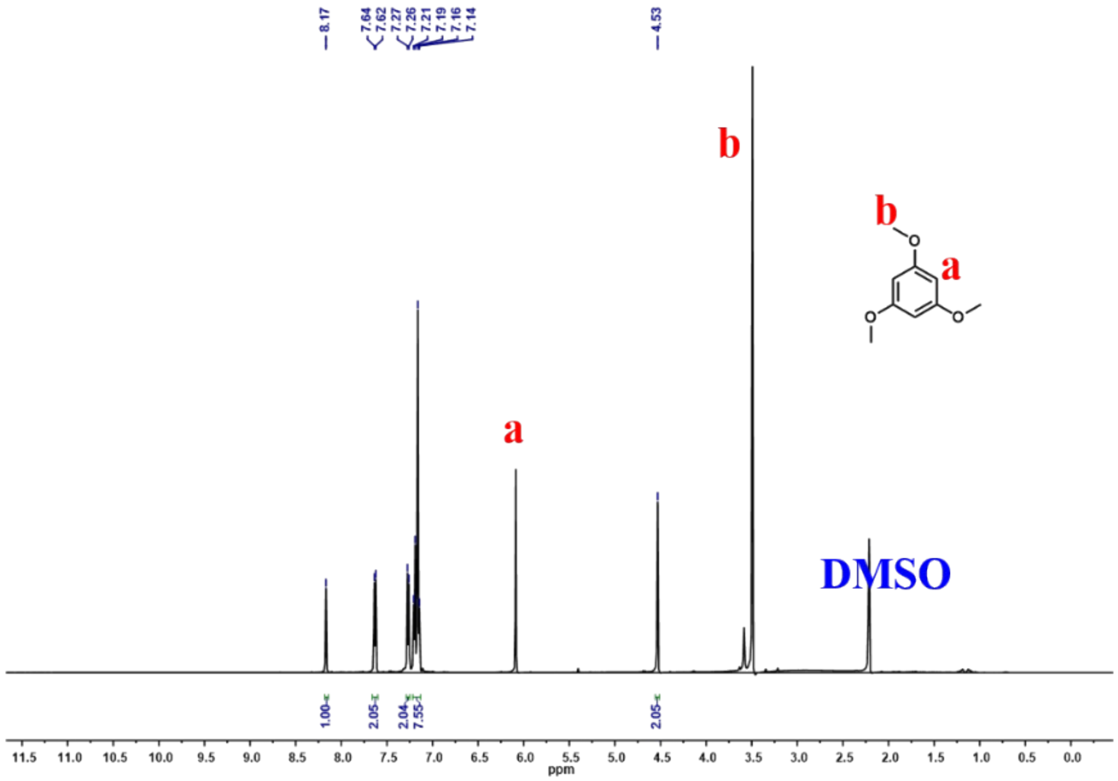

Figure S78. The ^{1}H NMR (500 MHz, 298 K) spectrum of compound **1h** in C_6D_6.

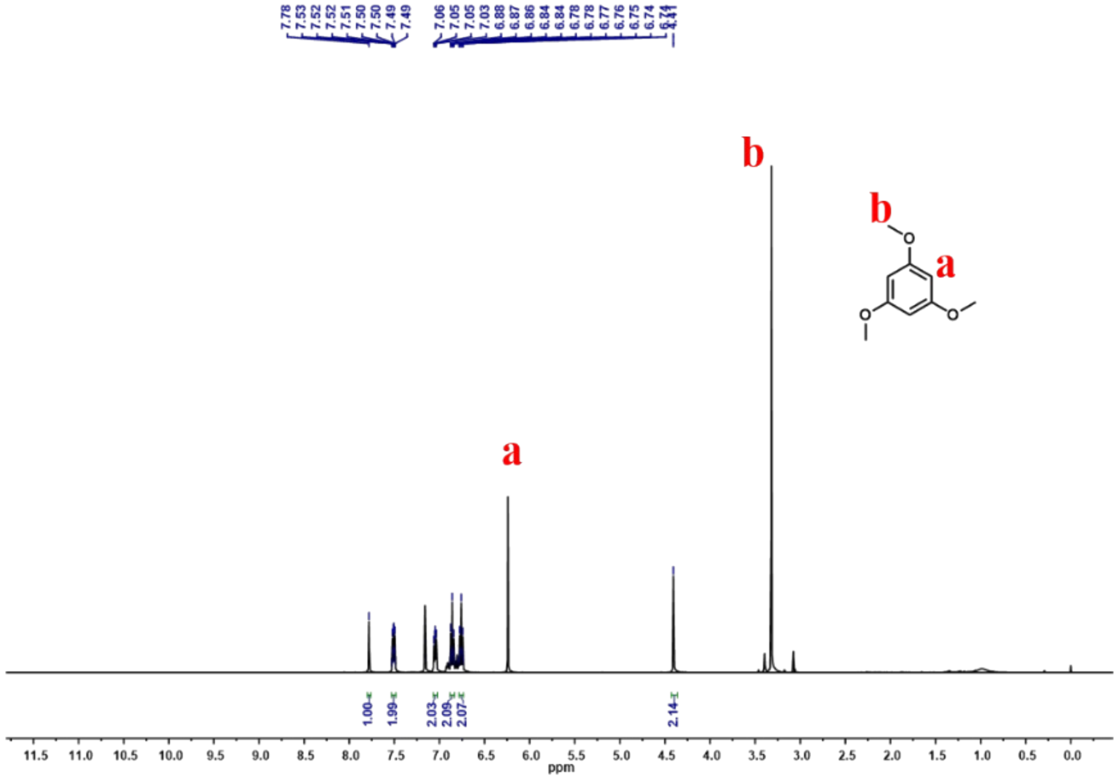

Figure S79. The ^{1}H NMR (500 MHz, 298 K) spectrum of compound **1i** in C_6D_6.

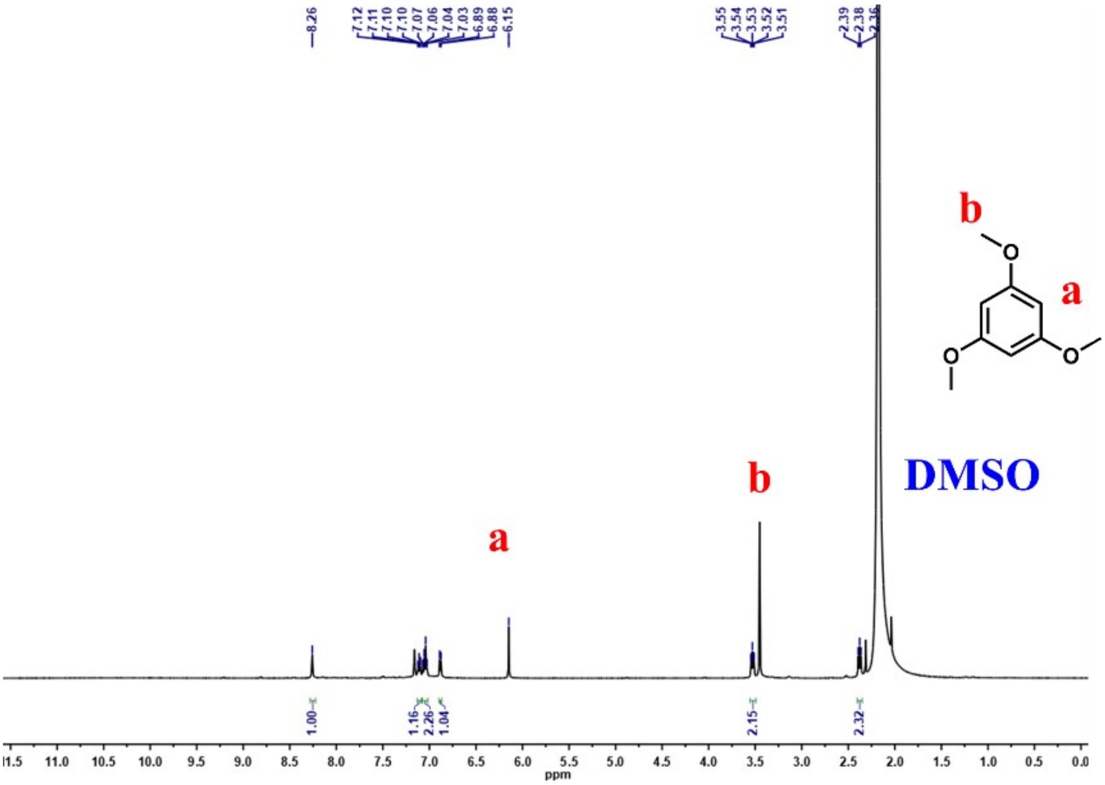

Figure S80. The ^{1}H NMR (500 MHz, 298 K) spectrum of compound **1j** in C_6D_6

10.3.2 In situ ^{1}H NMR spectra in Table 3.

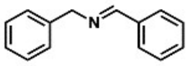

(**1a**) ^{1}H NMR (300 MHz, toluene-d_8) δ (ppm) 7.96 (s, 1H), 7.72-7.64 (m, 2H), 7.26 (d, J = 7.2 Hz, 2H), 7.20-7.14 (m, 2H), 7.12-7.08 (m, 4H), 4.57 (s, 2H).

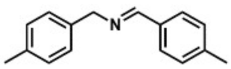

(**1b**) ^{1}H NMR (400 MHz, toluene-d_8) δ (ppm) 8.12 (s, 1H), 7.63 (d, J = 7.9 Hz, 2H), 7.17 (d, J = 7.7 Hz, 2H), 7.02-6.96 (m, 4H), 4.60 (s, 2H), 2.10 (s, 3H). Some product peaks overlap with solvent peak.

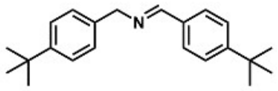

(**1c**) ^{1}H NMR (400 MHz, toluene-d_8) δ (ppm) 8.19 (s, 1H), 7.70 (d, J = 8.3 Hz, 2H), 7.27 (d, J = 8.3 Hz, 2H), 7.23 (s, 4H), 4.64 (s, 2H), 1.19 (s, 9H), 1.16 (s, 9H).

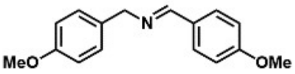

(**1d**) ^{1}H NMR (400 MHz, toluene-d_8) δ (ppm) 8.12 (s, 1H), 7.69 (d, J = 8.7 Hz, 2H), 7.19 (d, J = 8.5 Hz, 2H), 6.79 (t, J = 8.3 Hz, 4H), 4.59 (s, 2H), 3.45 (s, 3H). Some product peaks overlap with solvent peak.

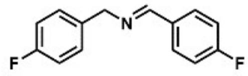

(**1i**) ^{1}H NMR (400 MHz, toluene-d_8) δ (ppm) 8.14 (s, 1H), 7.69 (dd, J = 8.2, 5.9 Hz, 2H), 7.19 (dd, J = 7.8, 6.0 Hz, 2H), 7.02-6.90 (m, 4H), 4.55 (s, 2H).

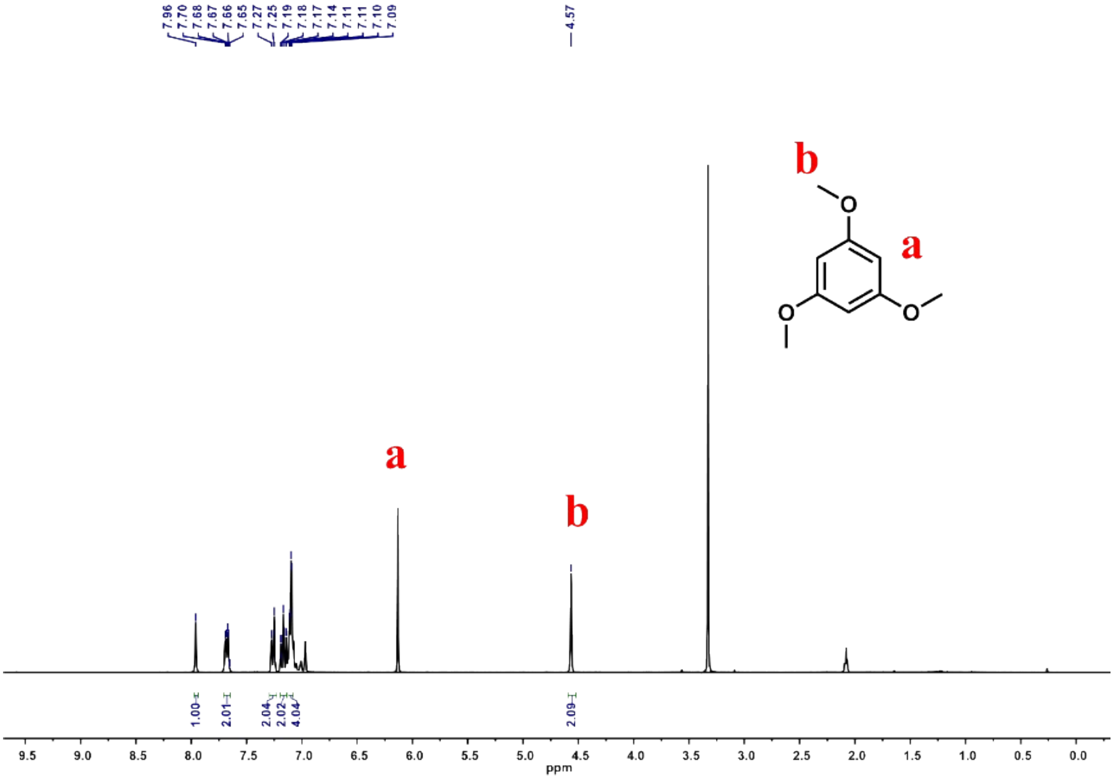

Figure S81. The ^{1}H NMR (300 MHz, 298 K) spectrum of compound **1a** in toluene-d_8.

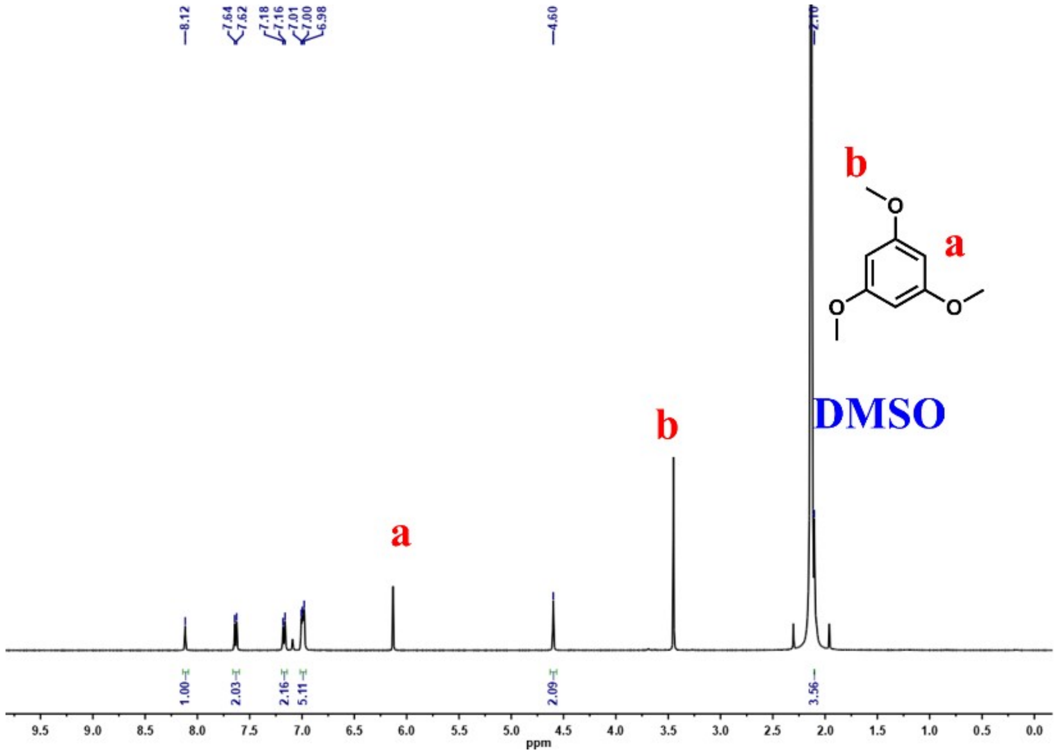

Figure S82. The ^1H NMR (400 MHz, 298 K) spectrum of compound **1b** in toluene-d_8.

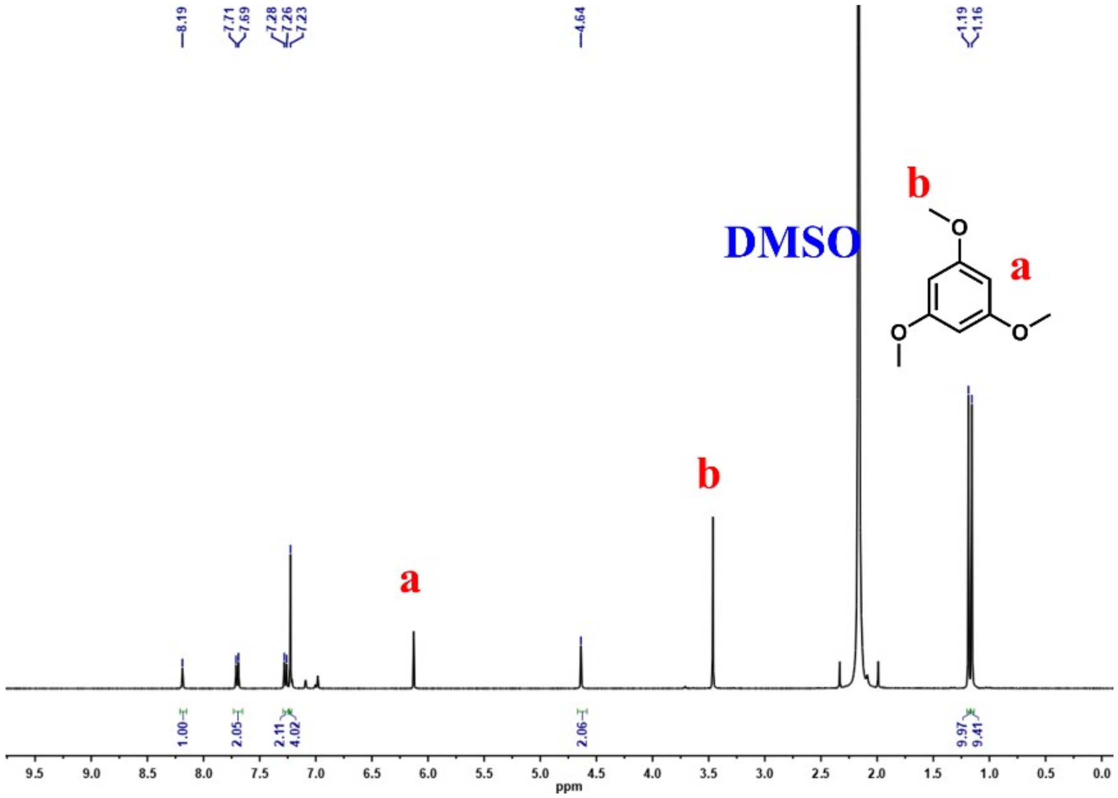

Figure S83. The ^1H NMR (400 MHz, 298 K) spectrum of compound **1c** in toluene-d_8.

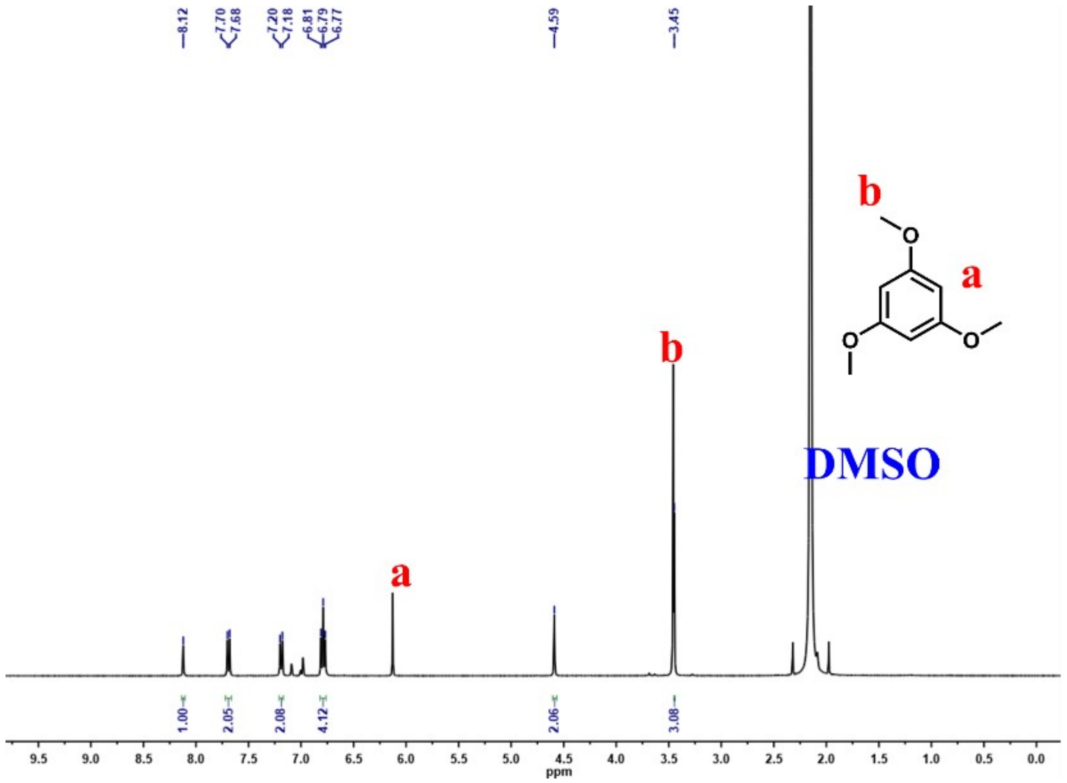

Figure S84. The ^{1}H NMR (400 MHz, 298 K) spectrum of compound **1d** in toluene-d_8.

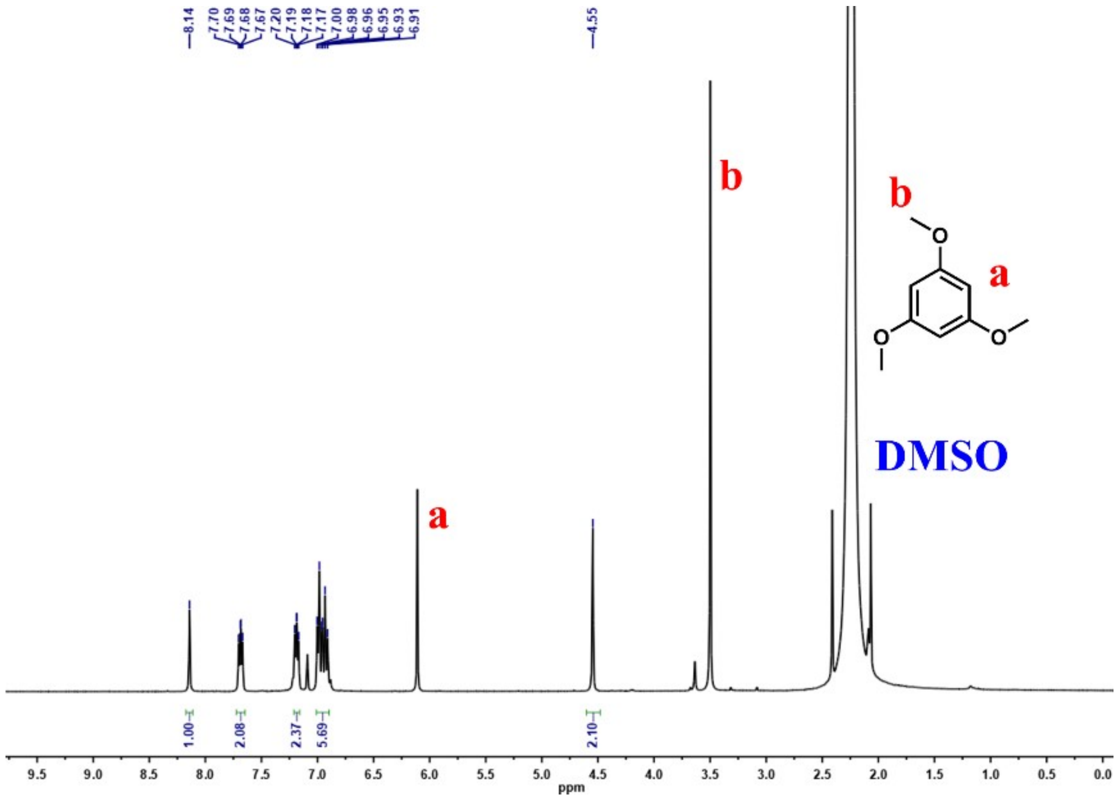

Figure S85. The ^{1}H NMR (400 MHz, 298 K) spectrum of compound **1i** in toluene-d_8.

11 GC-MS data of imines

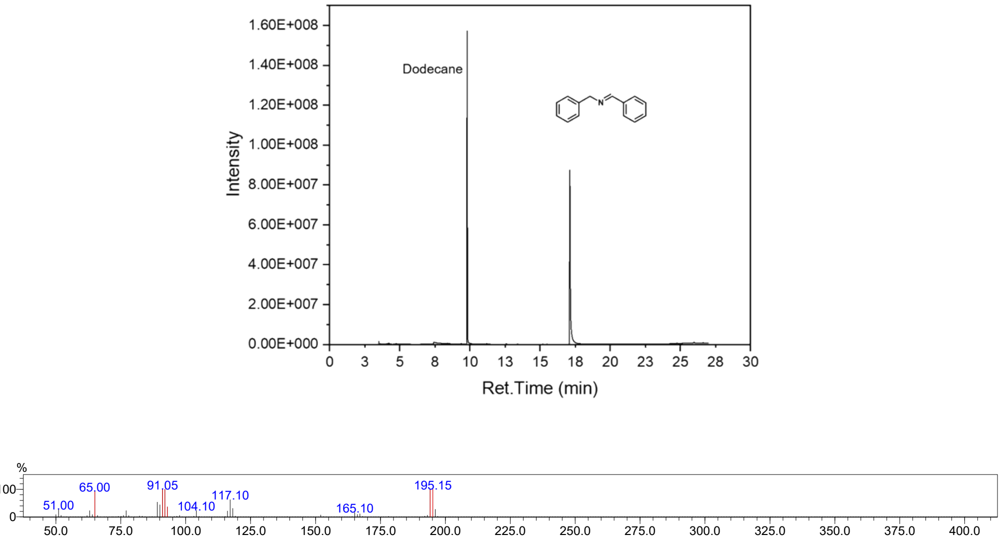

Figure S86. GC-MS result of product **1a**.

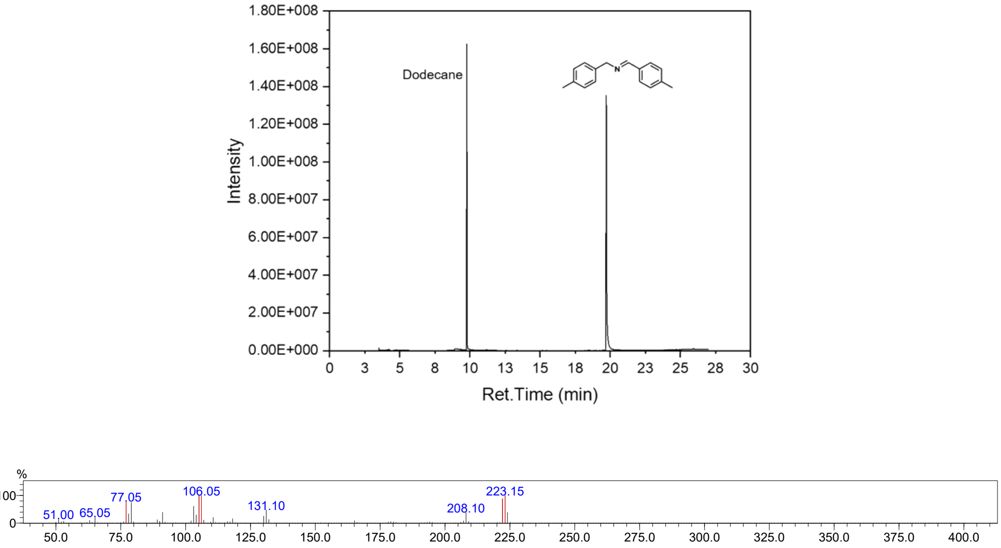

Figure S87. GC-MS result of product **1b**.

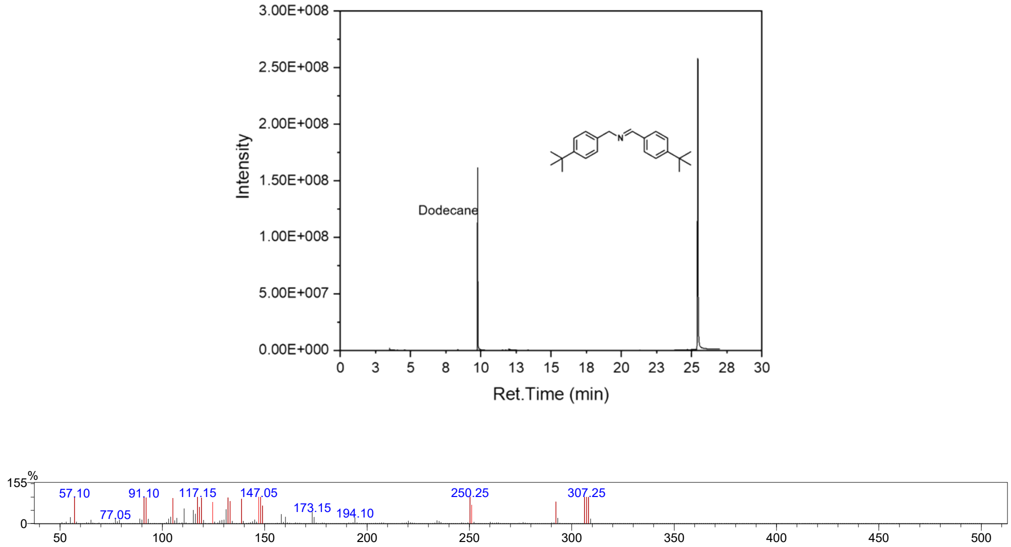

Figure S88. GC-MS result of product **1c**.

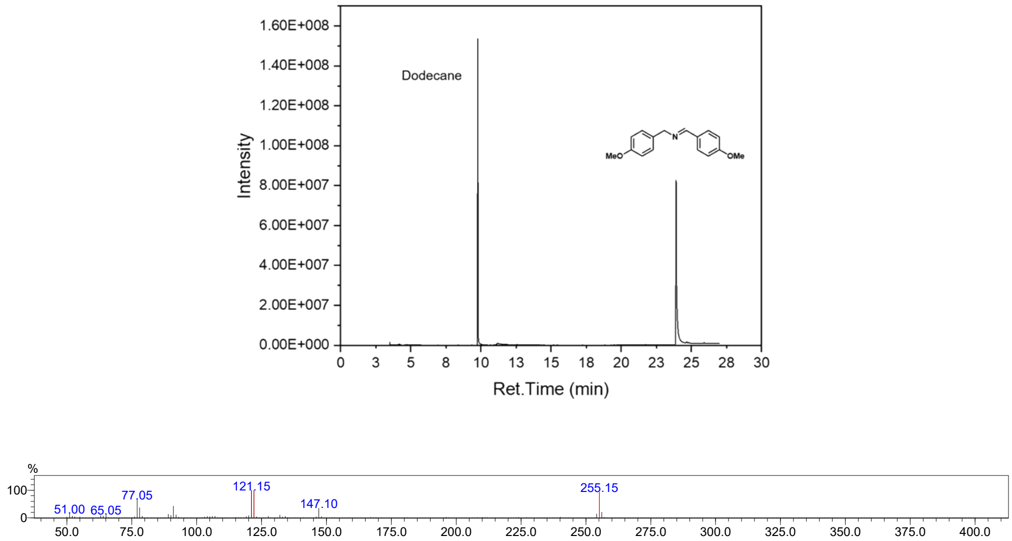

Figure S89. GC-MS result of product **1d**.

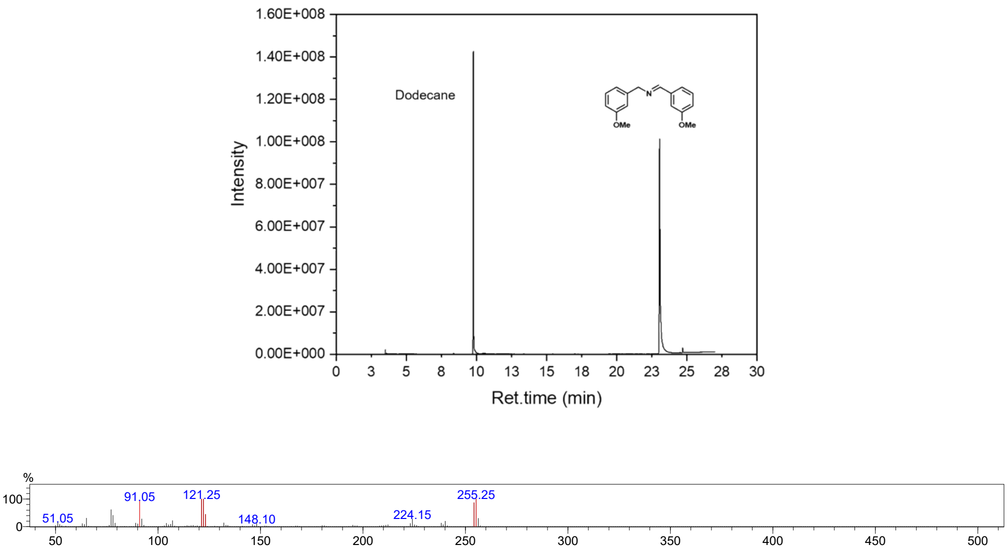

Figure S90. GC-MS result of product **1e**.

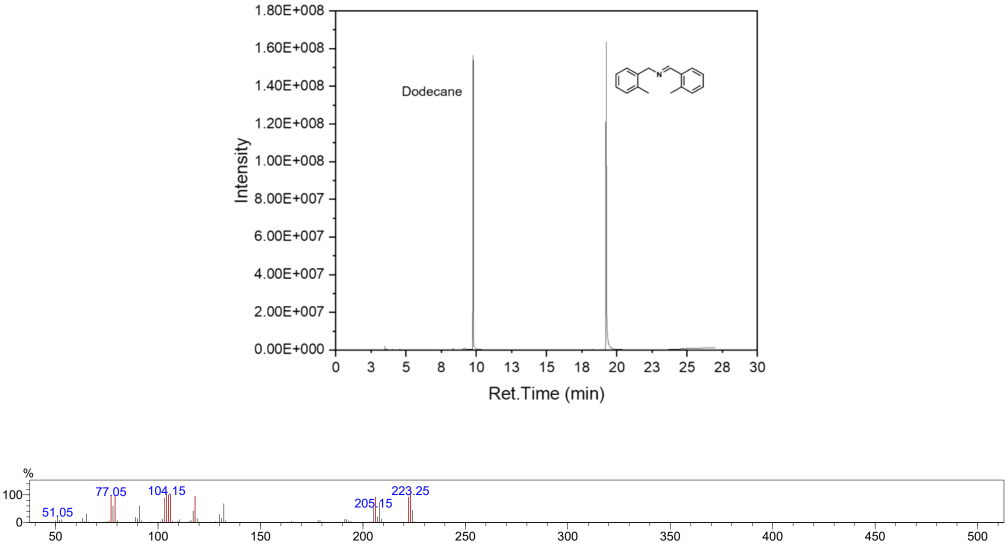

Figure S91. GC-MS result of product **1f**.

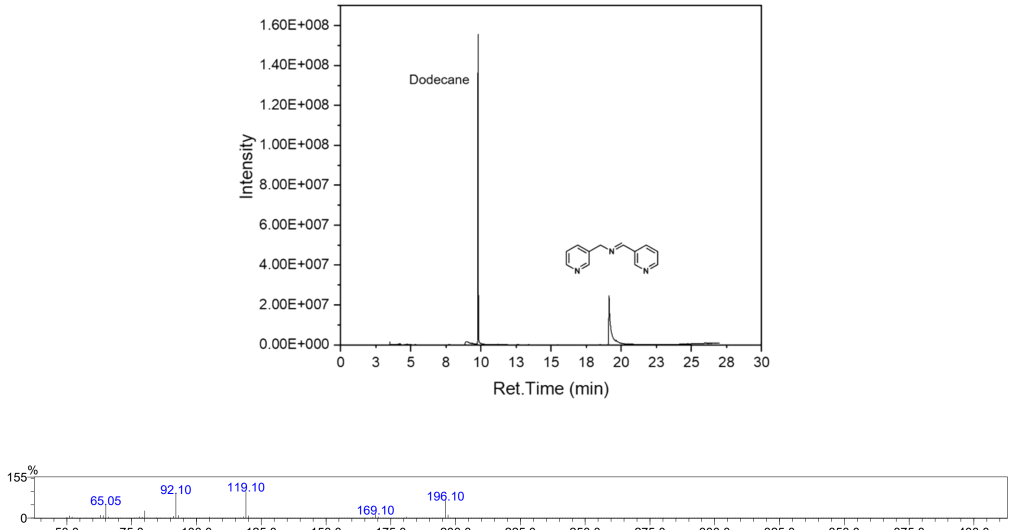

Figure S92. GC-MS result of product **1g**.

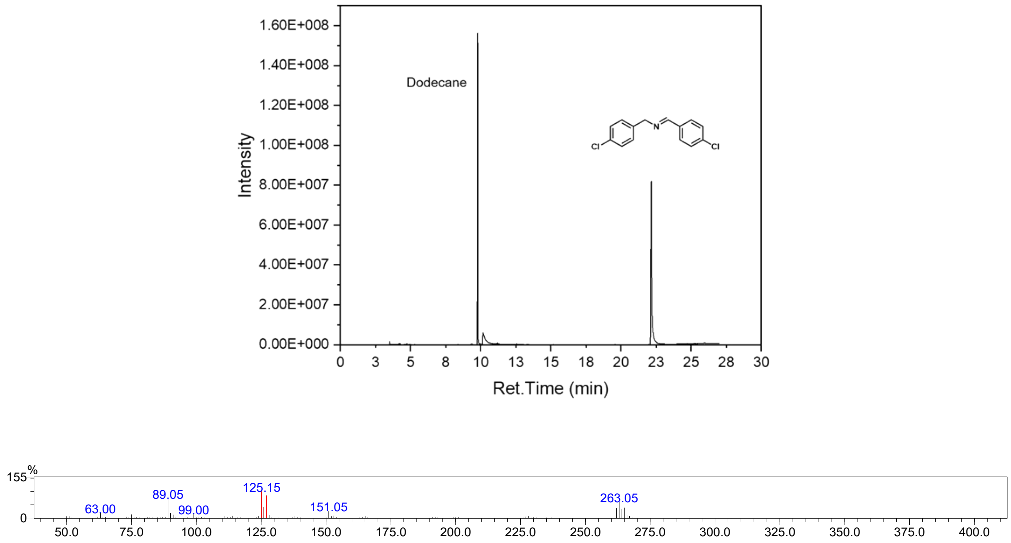

Figure S93. GC-MS result of product **1h**.

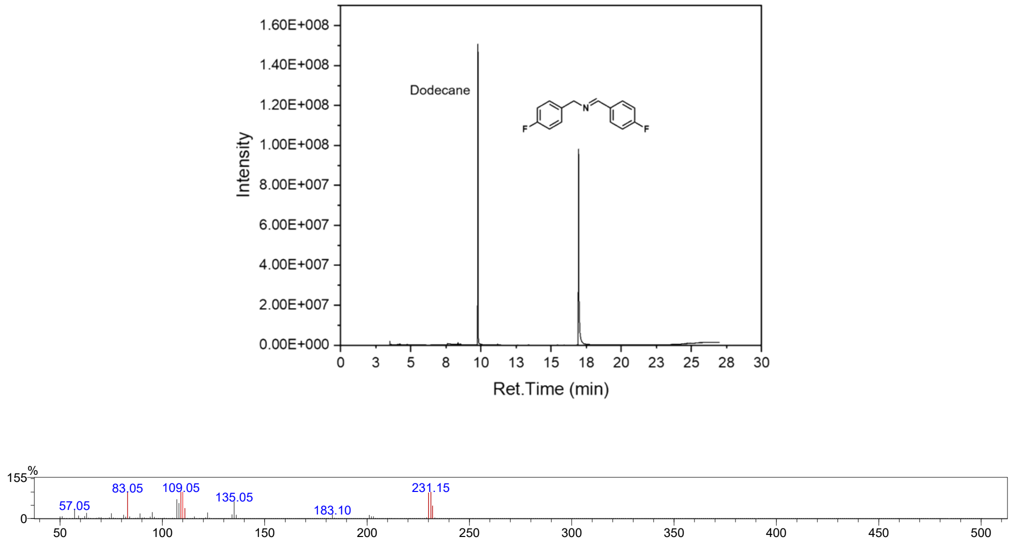

Figure S94. GC-MS result of product **1i**.

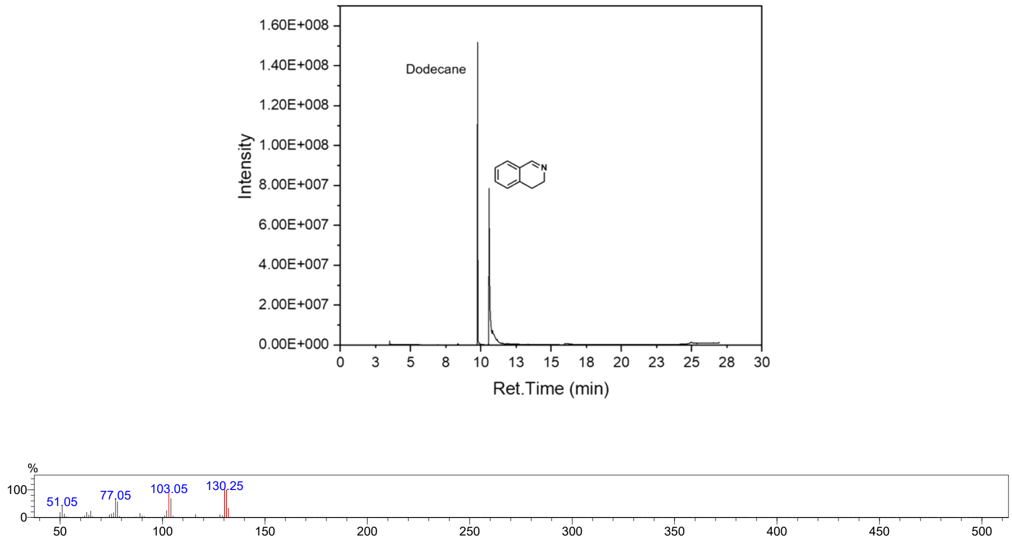

Figure S95. GC-MS result of product **1j**.

12 Mass spectra of new compounds

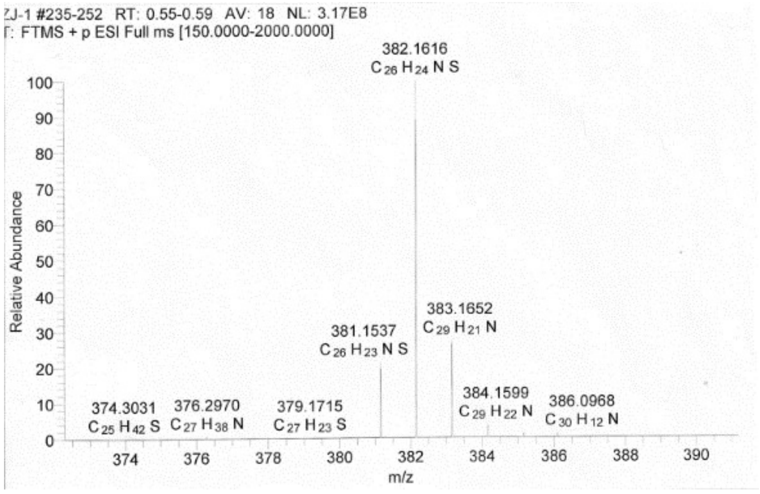

Figure S96. The mass spectrum of compound **1**.

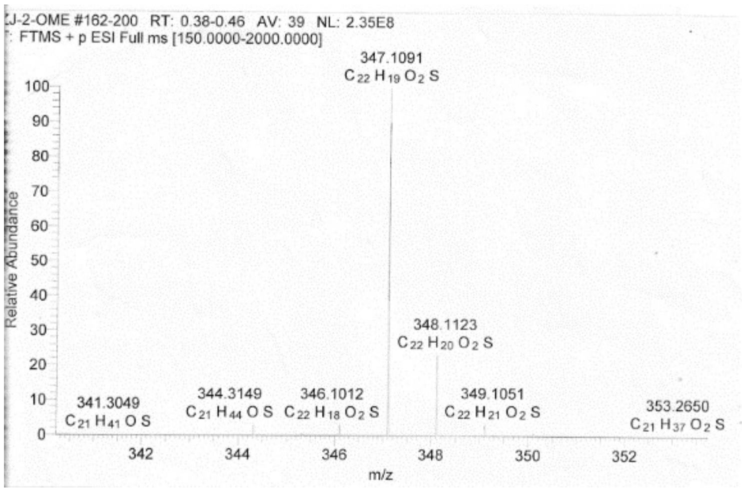

Figure S97. The mass spectrum of compound **b-OMe**.

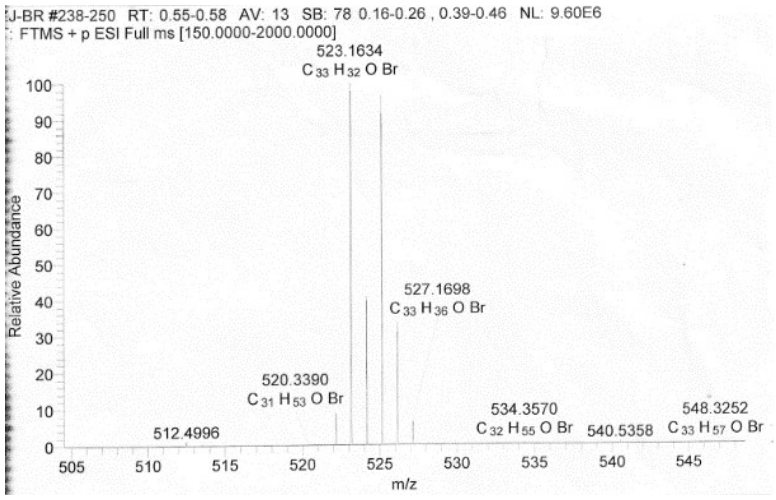

Figure S98. The mass spectrum of compound **2-Br**.

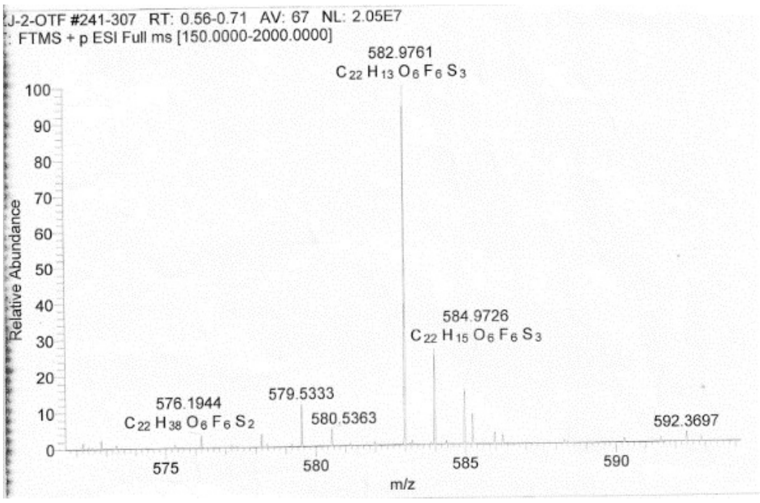

Figure S99. The mass spectrum of compound **b-OTf**.

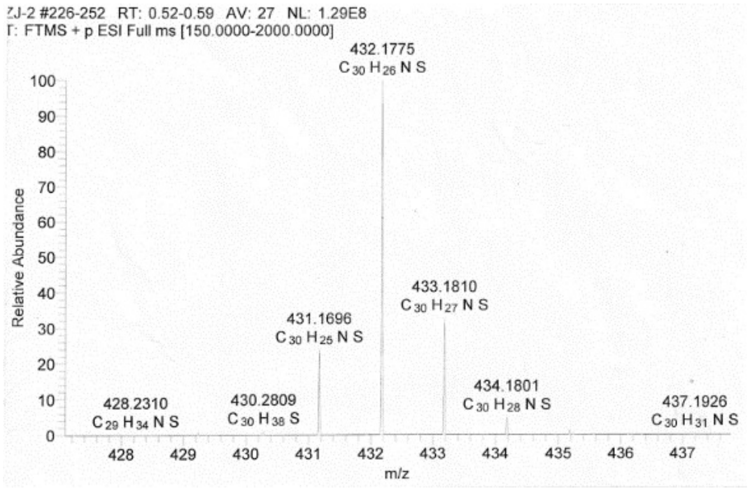

Figure S100. The mass spectrum of compound **2**.

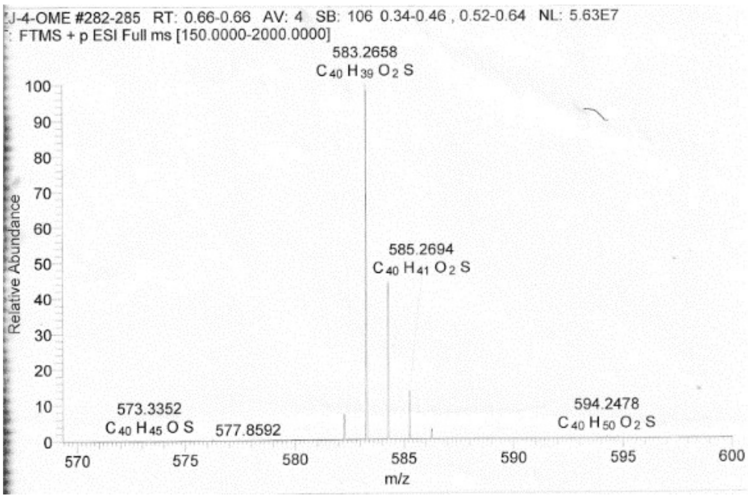

Figure S101. The mass spectrum of compound **c-OMe**.

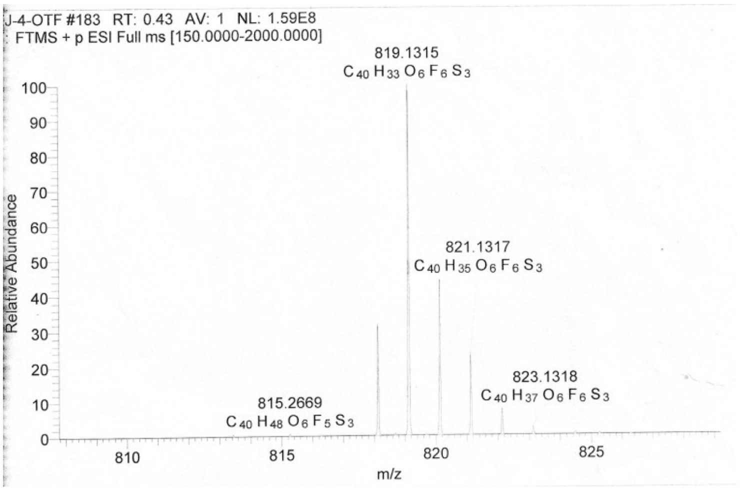

Figure S102. The mass spectrum of compound **c-OTf**.

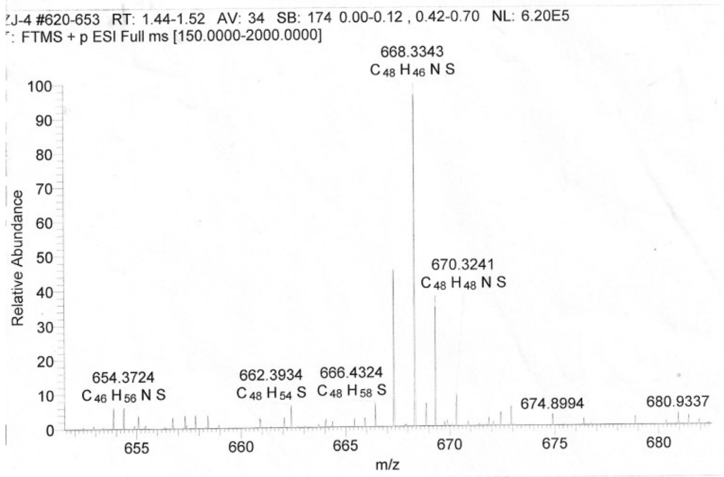

Figure S103. The mass spectrum of compound **3**.

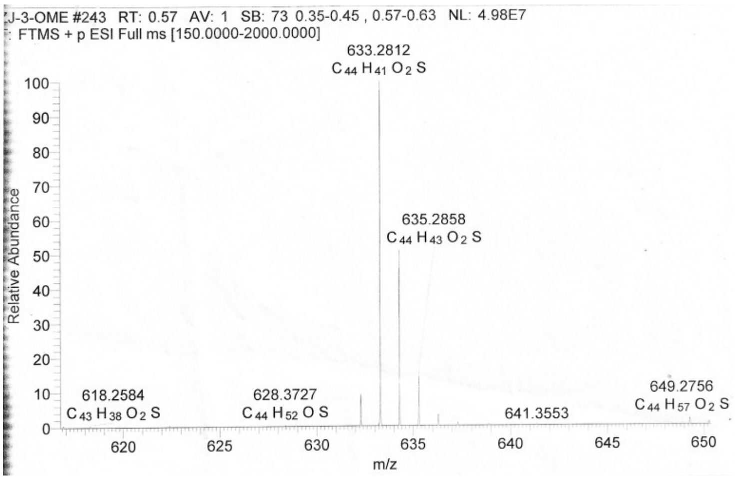

Figure S104. The mass spectrum of compound **d-OMe**.

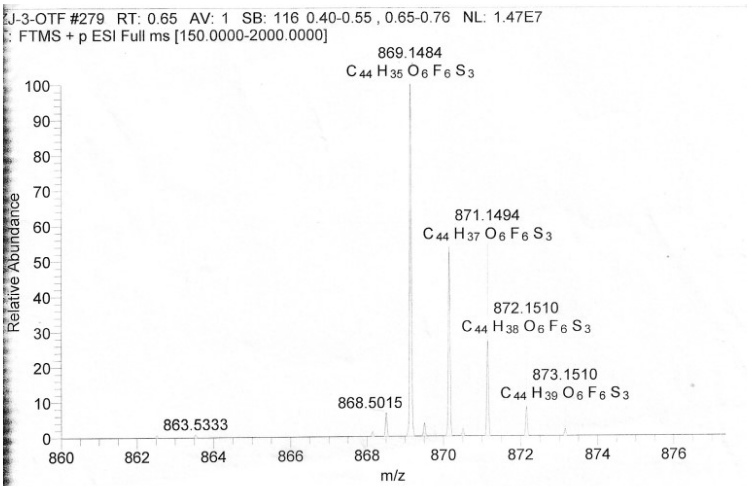

Figure S105. The mass spectrum of compound **d-OTf**.

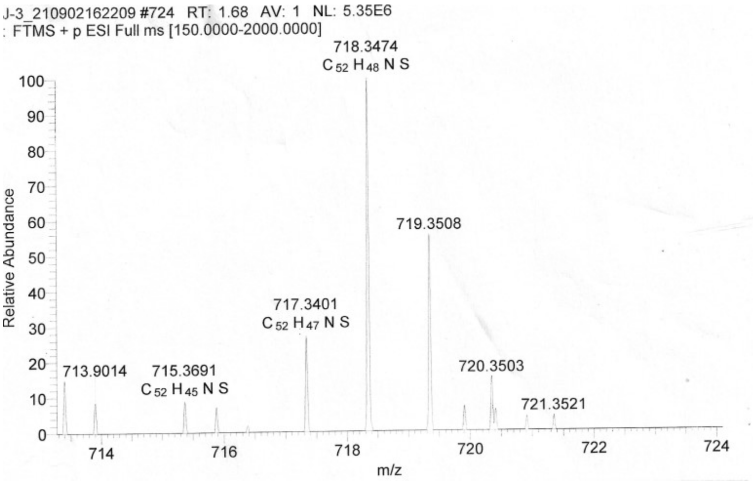

Figure S106. The mass spectrum of compound **4**.

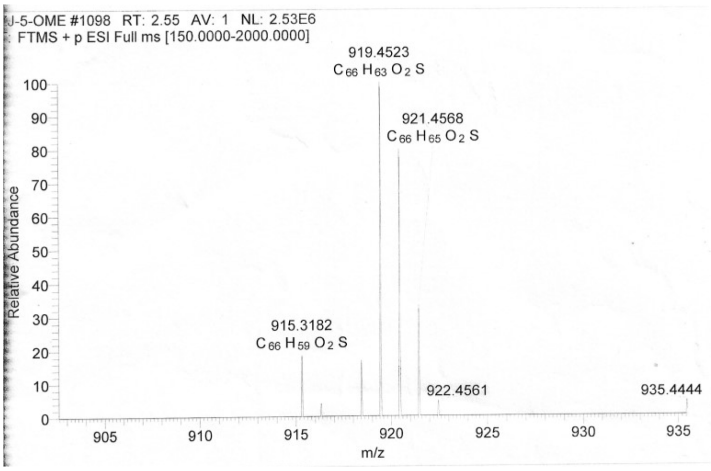

Figure S107. The mass spectrum of compound **e-OMe**.

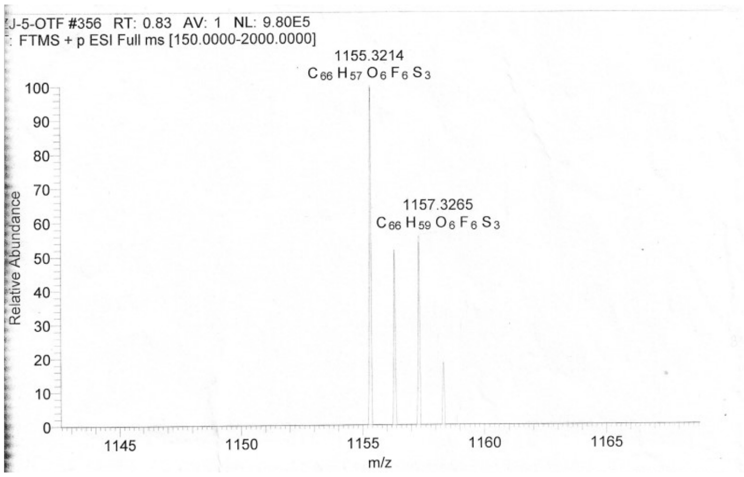

Figure S108. The mass spectrum of compound **e-OTf**.

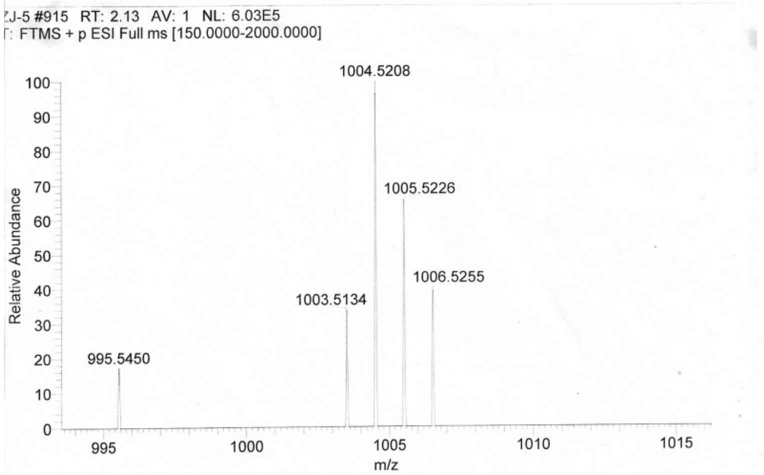

Figure S109. The mass spectrum of compound **5**.

13 Reference

[1] M. J. Frisch, G. W. Trucks, H. B. Schlegel, G. E. Scuseria, M. A. Robb, J. R. Cheeseman, G. Scalmani, V. Barone, G. A. Petersson, H. Nakatsuji, X. Li, M. Caricato, A. V. Marenich, J. Bloino, B. G. Janesko, R. Gomperts, B. Mennucci, H. P. Hratchian, J. V. Ortiz, A. F. Izmaylov, J. L. Sonnenberg, D. Williams-Young, F. Ding, F. Lipparini, F. Egidi, J. Goings, B. Peng, A. Petrone, T. Henderson, D. Ranasinghe, V. G. Zakrzewski, J. Gao, N. Rega, G. Zheng, W. Liang, M. Hada, M. Ehara, K. Toyota, R. Fukuda, J. Hasegawa, M. Ishida, T. Nakajima, Y. Honda, O. Kitao, H. Nakai, T. Vreven, K. Throssell, J. A. Montgomery Jr, J. E. Peralta, F. Ogliaro, M. J. Bearpark, J. J. Heyd, E. N. Brothers, K. N. Kudin, V. N. Staroverov, T. A. Keith, R. Kobayashi, J. Normand, K. Raghavachari, A. P. Rendell, J. C. Burant, S. S. Iyengar, J. Tomasi, M. Cossi, J. M. Millam, M. Klene, C. Adamo, R. Cammi, J. W. Ochterski, R. L. Martin, K. Morokuma, O. Farkas, J. B. Foresman and D. J. Fox, *Gaussian 16*, *Revision A.03*, Gaussian, Inc., Wallingford CT, 2016.

[2] T. Lu and F. Chen, *J. Comput. Chem.*, 2012, **33**, 580-592.
[3] W. Humphrey, A. Dalke and K. Schulten, *J. Mol. Graph*., 1996, **14**, 33-38.
[4] Z. Liu, T. Lu and Q. Chen, *Carbon*, 2020, **165**, 461-467.
[5] S. Dong, T. Y. Gopalakrishna, Y. Han, H. Phan, T. Tao, Y. Ni, G. Liu and C. Chi, *J. Am. Chem. Soc.*, 2019, **141**, 62-66.
[6] H. Xu, X. Li, H. Hao, X. Dong, W. Sheng and X. Lang, *Appl. Catal. B: Environ.*, 2021, **285**, 119796.
[7] F. Su, H. Peng, H. Yin, C. Luo, L. Zhu, W. Zhong and L. Mao, D. Yi, *J. Catal.*, 2021, **404**, 149-162.
[8] J.-J. Zhang, J.-M. Ge, H.-H. Wang, X. Wei, X.-H. Li and J.-S. Chen, *ChemCatChem*, 2016, **8**, 3441-3445.
[9] F. Raza, J. H. Park, H.-R. Lee, H.-I. Kim, S.-J. Jeon and J.-H. Kim, *ACS Catal.*, 2016, **6**, 2754-2759.
[10] Q. Shen, T. Xu, G. Zhuang, Y. Zhuang, L. Sun, X. Han, X. Wang and W. Zhan, *ACS Catal.*, 2021, **11**, 12931-12939.
[11] S. Boochakiat, D. Tantraviwat, O. Thongsook, S. Pornsuwan, A. Nattestad, J. Chen, D. Channei and B. Inceesungvorn, *Colloid Interface Sci.*, 2021, **602**, 168-176.
[12] Z. Wu, X. Huang, X. Li, G. Hai, B. Li and G. Wang*, Sci. China Chem.,* 2021, **64,** 2169-2179.
[13] X. Li, K. Zhang, X. Huang, Z. Wu, D. Zhao and G. Wang, *Nanoscale*, 2021, **13**, 19671-19681.
[14] F. Su, S. C. Mathew, L. Möhlmann, M. Antonietti, X. Wang, and S. Blechert, *Angew. Chem., Int. Ed.*, 2011, **50**, 657-660.
[15] K. Zhang, G. Lu, F. Chu and X. Huang, *Catal. Sci. Technol.*, 2021, **11**, 7060-7071.
[16] S. Juntrapirom, S. Anuchai, O. Thongsook, S. Pornsuwan, P. Meepowpan, P. Thavornyutikarn, S. Phanichphant, D. Tantraviwat and B. Inceesungvorn, *Chem. Eng. J.*, 2020, **394**, 124934.
[17] K. Zhang, Z. Xi, Z. Wu, G. Lu and X. Huang, *ACS Sustainable Chem. Eng.*, 2021, **9**, 12623-12633.
[18] X. Li, Z. Li, K. Zhang, D. Zhao and X. Huang, *Asian J. Org. Chem.,* 2021, **10**, 2895-2901.
[19] H. He, X. Fang, D. Zhai, W. Zhou, Y. Li, W. Zhao, C. Liu, Z. Li and W. Deng, *Chem. Eur. J.*, 2021, **27**, 14390-14395.
[20] X. Yang, T. Huang, S. Gao and R. Cao, *J. Catal.*, 2019, **378**, 248-255.
[21] Y. Xiao, G. Tian, W. Li, Y. Xie, B. Jiang, C. Tian, D. Zhao and H. Fu, *J. Am. Chem. Soc.*, 2019, **141**, 2508-2515.
[22] C. Xing, G. Yu, T. Chen, S. Liu, Q. Sun, Q. Liu, Y. Hu, H. Liu and X. Li, *Appl. Catal. B: Environ.,* 2021, **298**, 120534.
[23] X. Ye, Y. Li, P. Luo, B. He, X. Cao and T. Lu, *Nano Res.*, 2022, **15**, 1509-1516.
[24] J. Jiang, X. Liu and R. Luo, *Catal. Lett.*, 2021, **151,** 3145-3153.
[25] Y. Xu, Y. Chen and W.-F. Fu, *Appl. Catal. B: Environ.*, 2018, **236**, 176-183.
[26] K. Gao, H. Li, Q. Meng, J. Wu and H. Hou, *ACS Appl. Mater. Interfaces*, 2021,

13, 2779-2787.

[27] Y. Liu, Y. Chen, W. Zhou, B. Jiang, X. Zhang and G. Tian, *Catal. Sci. Technol.*, 2018, **8**, 5535-5543.

[28] L.-H. Meng, X.-P. Tan, L. Chen, S. Shen, J.-K. Guo, C.-T. Au and S.-F. Yin, *Appl. Surf. Sci.*, 2021, **541**, 148476.
